# Maize heat shock proteins—prospection, validation, categorization and in silico analysis of the different ZmHSP families

**DOI:** 10.1007/s44154-023-00104-2

**Published:** 2023-09-06

**Authors:** Rubens Diogo-Jr., Edila Vilela de Resende Von Pinho, Renan Terassi Pinto, Lingrui Zhang, Jorge Alberto Condori-Apfata, Paula Andrade Pereira, Danielle Rezende Vilela

**Affiliations:** 1 Department of Horticulture and Landscape Architecture, Purdue University, West Lafayette, IN (47907) USA; 2 Department of Agriculture, Federal University of Lavras (UFLA), Lavras, MG (37200-900) Brazil; 3 Faculty of Philosophy and Sciences at Ribeirao Preto, University of Sao Paulo (USP), Ribeirao Preto, SP (14040-901) Brazil; 4 Faculty of Engineering and Agricultural Sciences, Universidad Nacional Toribio Rodriguez de Mendoza de Amazonas (UNTRM), Chachapoyas, AM (01001) Peru

**Keywords:** Biotechnology, Genomics, Corn, Stress, Drought, Compendium

## Abstract

Among the plant molecular mechanisms capable of effectively mitigating the effects of adverse weather conditions, the heat shock proteins (HSPs), a group of chaperones with multiple functions, stand out. At a time of full progress on the omic sciences, they look very promising in the genetic engineering field, especially in order to conceive superior genotypes, potentially tolerant to abiotic stresses (AbSts). Recently, some works concerning certain families of maize HSPs (ZmHSPs) were published. However, there was still a lack of a study that, with a high degree of criteria, would fully conglomerate them. Using distinct but complementary strategies, we have prospected as many ZmHSPs candidates as possible, gathering more than a thousand accessions. After detailed data mining, we accounted for 182 validated ones, belonging to seven families, which were subcategorized into classes with potential for functional parity. In them, we identified dozens of motifs with some degree of similarity with proteins from different kingdoms, which may help explain some of their still poorly understood means of action. Through in silico and in vitro approaches, we compared their expression levels after controlled exposure to several AbSts' sources, applied at diverse tissues, on varied phenological stages. Based on gene ontology concepts, we still analyzed them from different perspectives of term enrichment. We have also searched, in model plants and close species, for potentially orthologous genes. With all these new insights, which culminated in a plentiful supplementary material, rich in tables, we aim to constitute a fertile consultation source for those maize researchers attracted by these interesting stress proteins.

**Supplementary Information:**

The online version contains supplementary material available at 10.1007/s44154-023-00104-2.

## Introduction

Plants, organisms restricted by the place where they germinate / sprout, are repeatedly exposed, throughout their entire life cycle, to innumerable adverse weather conditions, over which they have no control. A common element resulting from exposure to most AbSts is precisely cellular dehydration. This, in turn, induces the abscisic acid (ABA) biosynthesis and, thereafter, to significant changes in the concentration of osmoprotective solutes (accumulated in the cytosol and important for the membranes composition and viscosity), which makes the cells unable to withstand high osmotic exchanges (Masouleh et al. 2019). Soon after, irreversible damage to membranes and cell walls accumulates. Concomitantly, several imbalances are installed in the cellular environment: damages in the photosynthetic machinery, interruption of the electron transport system on the inner mitochondrial membrane, deficits in the ATP-form energy generation, ROS production, etc. This chain of events causes numerous losses to the stressed plants, may leading to cell collapse and apoptosis, generally due to unbalanced activity of the phytaspases and metacaspases enzymes (Huh 2022). On the other hand, due to their stiff nature, it was imperative that plants have evolved, over the generations, mechanisms that alleviate these penalties, allowing their survival in once inhospitable places. Thus, they generally respond to such stressful conditions through physiological, morphological and metabolic processes, correlated as for the action effectiveness (Zandalinas et al. 2020). Among them, it is worth highlighting the HSPs contribution, above all on maize crop.

Conceptually, HSPs constitute a category among the so-called “stress proteins”, being highly conserved and present in all cells of all organisms. A special group among chaperones, they play a crucial role structuring some proteins, folding them, maintaining their functional conformations, assembling multiprotein complexes and preventing against non-native proteins aggregation. They are also involved in the protein translocation process, controlling the cell signaling and the transport to the suitable subcellular compartment, and in the degradation of other proteins, avoiding the eventual formation of non-functional ones. In addition, they increase membrane stability and help during the ROS detoxifying process by positively regulating the antioxidant enzyme system. In plants, they act greatly in AbSts' situations (extreme temperatures, drought, flooding, high salinity or exposure to various chemical compounds) and even biotic stresses, due to accumulation / stabilization of proteins related to pathogenesis (Ul et al. 2019), supporting the homeostasis maintenance, increasing cell survival and, finally, preventing against necrosis and/or apoptosis resulting from this process.

Recently, it has been found that their expression were induced by silicon (Khan et al. 2020), ascorbic acid exogenous application (Elkelish et al. 2020), excessive light and UV-rays (Kim et al. [Bibr CR45][Bibr CR46]), as well as by the humic acid levels raise on soil (Cha et al. 2020). Furthermore, they are regulated by a series of transcription factors (TFs) (Barua et al. 2022). In some cereals, the expression was also increased in thermo-conditioning processes (Kushawaha et al. 2021) and after exposure to thermal, water or saline stress, beyond the heavy metals accumulation (Chaudhary et al. 2019). Focusing particularly on maize, a crop so dependent from biotechnology-assisted breeding, but also in which adverse environmental situations have been causing significant yield losses (El-Sappah et al. 2022), it becomes indispensable to understand more about such molecules.

HSPs can be classified in different ways: regarding their amino acid (AA) sequences; based on the presence of certain conserved domains (CD); and mainly according to their molecular weight (MW) in kDa. Thus, they are routinely organized into six large families: HSP100s/CLPs, active during protein aggregates solubilization; HSP90s, HSP70s and chaperonins, the three groups that mediate protein folding; HSP40s/DNAJs, regulators of the HSP70 complex; and SmallHSPs, which have low MW and protect against protein aggregation.

HSP100s are fundamental for disaggregation of proteins and other high MW complexes inactivated by heat, also for thiamine biosynthesis (Rapala-Kozik et al. 2012), for chloroplast development and protein turnover (Mishra & Grover 2016). In addition to thermal stress, the plant phenological stage regulates their expression as well. Besides that, they play an important role in sugar maintenance and starch synthesis, as well as in the root growth and architecture (Lázaro-Mixteco et al. 2012). Plant cells, in particular, have multiple forms of these proteins, which are located in several organelles. They harbor, in the 5'UTR region, an internal ribosome entry site (IRES), which allows interaction with other molecules (including other HSP100s) and the translation initiation independently of the 5'Cap, considerably increasing protein synthesis (Jiménez-González et al. 2014). Relevant to the repair mechanism in eventual double-stranded DNA breaks (DSBs), they also induce thermotolerance, even during microsporogenesis, which results in microspores better adapted to high temperatures (Li et al. [Bibr CR54][Bibr CR55]). In maize seeds, they are accumulated during embryo maturation and desiccation, persisting at high levels on the first 24 h following imbibition; so, they remain active in the balance mechanisms during germination, participating in the de novo synthesis of some proteins, as well as in the metabolic pathways of actin and cytokinin (Lázaro-Mixteco et al. 2012). Seeds treated with cold plasma jets at atmospheric pressure (APPJ) (Holubová et al. 2021), besides plants exposed to intense light and thermal shock, especially in the procambial region (where the nodal roots formation occurs), exhibit a significant increment of the ZmHSP100s expression levels (López-Frías et al. 2011).

HSP90s are decisive in drought (Zhang et al. 2021) and heat (Guo et al. [Bibr CR29][Bibr CR30]; Li et al. 2020a) tolerance mechanisms, acting in two evolutionarily conserved systems to protect plants against such stresses: the “unfolded protein response” (UPR) and the “thermal shock response” (HSR) (Li et al. [Bibr CR51][Bibr CR52]). Actively participating in both signal transduction pathways and disease resistance mechanisms (Wei et al. 2021), they are made up of three basic domains: one for ATP binding, at N-terminal; one medial; and another for dimerization, at C-terminal, which allows the interaction with different substrates (including other chaperones) and the coupling to ATPases, which implies some conformational changes and, therefore, the enforcement of different biological functions (Genest et al. 2019). They participate in the MAPK cascade, playing a fundamental role during apoptosis and the hypersensitivity response, preventing the infection spread (Chen et al. 2022). Moreover, HSP90s are crucial for maintaining the genome integrity against transposons (Specchia & Bozzetti 2021) and also during energy generation, as they carry out the “quality control” of the protein folding process in mitochondria, supporting nitrogen metabolism and starch synthesis in plants subjected to heat stress (Altieri 2013). Furthermore, they are up-regulated after hormonal signaling (Wang et al. 2020a), after pre-treatment with cAMP (Zhao et al. 2021), during virus infection (Vargas & Rodriguez 2022) and ectopic expression of heterologous glutaredoxins, culminating in antioxidant system optimization and yield increases (Sprague et al. 2022).

HSP70s are indispensable to cellular machinery involved in proteostasis (Genest et al. 2019), constituting the main group responsible for protein folding and remodeling, being also essential for chloroplast development, peptide degradation, transmembrane transport and protein–protein interactions (De Los Rios & Goloubinoff 2016). Composed of two functional domains, one for ATPases binding and another for the proteins to be assisted, they are closely related to the chaperones BIP and DNAK (Bonomo et al. 2010). Universally distributed, but structurally conserved on different species, they participate in the response mechanisms to several kinds of AbSts (Amare & Kebede 2021), mainly those triggered by drought (Davoudi et al. 2022; Wu et al. 2021) and thermal shock (Guo et al. 2021a), with a prominent role in maize (Jiang et al. 2021; Li et al. 2020a), wheat and rice. They are also relevant before other adversities, such as excessive salinity and those implying in H_2_O_2_ generation (with subsequent oxidative damage), like severe exposure to UV radiation (Celinna et al. 2022), cold (Sowiński et al. 2020; Xue et al. 2021), flood events, heavy metals accumulation, acidification processes (Yusof et al. 2022) and soil contamination. Crucial in cross-tolerance arising from signal transduction pathways (Jiang et al. 2021), HSP70s integrate mechanisms that affect nutrient uptake by roots (Griffiths et al. 2021), inflorescence architecture on the ear (Li et al. 2022a) and leaf insertion angle in maize (Zhang & Huang 2021). Besides, they regulate the HSP's transcriptional factors (HSFs) activity (Tian et al. 2021), and act in the stress-induced activation of some transposons (Ugo et al. 2019). Involved in biotic interactions, they also drive the immunity triggered by effector proteins associated with phytopathogenic insects, bacteria and fungi (Berka et al. 2022). However, even though they also participate in the viral stress response (Ghorbani et al. 2022), the HSP70s accumulation could be virus-friendly under certain circumstances, as they help in the viral RNAs structuring and expression (Nagy et al. 2011). Regulated by different molecules, particularly phytohormones, they are accumulated in different organelles, which may be associated with functional diversification (Jiang et al. 2021).

Chaperonins (CPNs) are cylindrical-shaped “nanomachines” that operate as containers for the restructuring of various domains and/or protein subunits, avoiding or even reversing any inappropriate association between them (Horwich & Fenton 2020). They also act on cell-to-cell traffic and meristematic functions (Xu et al. 2011). Usually, they are classified into two groups: I, found in eubacteria, but also in mitochondria and chloroplasts; and II, in *Archaea* and eukaryotes in general. Those in Group II make the TRiC/CCT complex, a large hetero-oligomeric assembly composed of two “stacked rings”, which form a central cavity (“barrel”) where non-native polypeptides are folded, with ATP expenditure. The asymmetry of the barrels conformational cycles, deriving from the different TRiC/CCT subunits, underlies their unique ability to fold certain proteins (about 10% of eukaryotic proteomes) that could not be structured by other chaperones (Gestaut et al. 2019). Meanwhile, those in Group I are divided into two subfamilies, which act “in partnership”: the GroEL (hollow and porous cylinder) and the GroES (similar to a “dome”). Under ATP presence, both bond to each other, creating a “chamber” that encapsulates the proteins. In this harmonious microenvironment, a couple intermolecular interactions lead to its correct folding (Hayer-Hartl et al. 2015). This system, which targets specific substrates (like proteins damaged by AbSts), regulates in the transcriptional and post-translational levels; so, it is of paramount importance for the “quality control process" regarding proteins newly synthesized by chloroplast ribosomes (including RuBisCO) (Hotto et al. 2021). That is why all photosynthetic eukaryote genomes encode multiple chaperonin genes (Zhao & Liu 2018).

HSP40s constitute, without a doubt, the most complex group among the ZmHSPs, being composed of many heterogeneous subfamilies. As HSP70's machinery co-chaperones, they are responsible for the target substrate recognition and interaction, besides the ATPase activity stimulation (Musskopf et al. 2018). Soon after substrate selection, the HSP40-substrate complex induces ATP hydrolysis, leading to HSP40 dissociation and consequent formation of the high affinity complex HSP70-substrate. Although sharing a "J main” domain at the N-terminus, through which interaction with the HSP70s occurs, three other domains are fundamental in their structure: the G domain, immediately downstream of the previous one; a “central” domain, which forms a hydrophobic “pocket” for dimerization processes, increasing the HSP40s' affinity with their substrates; and a domain positioned at C-terminus, where the CTDI and CTDII regions are located, both necessary to keep the J domains in their relatively specific positions (Musskopf et al. 2018). Eventual J domain modifications make some HSP40s able to optimize the activity of certain enzymes with which they interact (Mattoo et al. 2014). In rice, they are known to regulate up to leaf size (Wang et al. 2022). However, the number of papers investigating the maize HSP40s is still relatively small, while it is already clear that their expression levels are increased after thermal stress, notedly during the seedling phase (Qian et al. 2019).

SmallHSPs (which have low MW, < 40 kDa) perform the “cellular defense first line” against stress from excessive heat and/or others that cause protein denaturation (Janowska et al. 2019). All organisms own them, indicating they have evolved very early, before the divergence of the three life domains. They form a very diverse chaperone family, which share an α-crystalline domain flanked by variable and disordered N- and C-terminal extensions, even though certain structural characteristics (*e.g.* an oligomeric form with a β-sandwich domain) tend to remain conserved (Waters & Vierling 2020). In fact, stressful situations, besides causing significant protein conformational rearrangements, resulting in specific binding sites exposure, imply a SmallHSPs' levels rapid increase. They couple, in an ATP-independent manner, to those unstructured proteins, forming large oligomeric complexes, which are stored in inactive forms and function as “sorting centers” for different “quality control pathways”, including the refolding mediated by HSP70s and a kind of “selective autophagy” process (Mogk et al. 2019). This “sequestration”, besides protecting proteins against possible uncontrolled aggregation, also facilitates their refolding by HSP100 disaggregases (Janowska et al. 2019). Certain plant species have dozens of SmallHSPs, which are expressed in many cell compartments, as well as on different development stages. They accumulate in chloroplasts, where, interacting with innumerous proteins, participate in the O_2_ evolution during photosystem II, even under excessively hot conditions (Hu et al. 2015). In mitochondria, they protect against protein denaturation and degradation (Rashed et al. 2022). They also interact with brassinosteroid signaling kinases, conferring salt stress tolerance (Liu et al. [Bibr CR57][Bibr CR58]), as well as with calcium-dependent (and ABA-induced) kinases, which mediate their phosphorylation, essential for their chaperone function (Yulong et al. 2021). Under AbSts' situations, they perform multiple functions, but with different efficiency levels, reflecting the existence of predefined substrates for each SmallHSP within those different intracellular compartments. They are also associated with the presence of some storage proteins, being accumulated in a couple energy reserve organs (Kim et al. 2020). It is known their adoption as biomarkers for screening cultivars with different capacities to tolerate heat stress (X. Chen et al. 2014). In maize, there are several reports of significant SmallHSP expression increases induced by drought (K. H. Kim et al. 2021a; Li et al. 2021) and/or heat stress (El-Sappah et al. 2022; Liu et al. 2022a), as well as such after accumulation (or exogenous application) of hydrogen peroxide. When overexpressed, they confer tolerance to heat and oxidative stress (Sun et al. 2012). Active also during mesocotyl growth, as well as in photosynthetic carbon metabolism reprogramming (particularly under high CO_2_ conditions), they are also accumulated in ABA-deficient mutants. In some QTL studies, SmallHSPs have already been associated with physiological characteristics related to seed longevity (Arif et al. 2022), senescence (Caicedo et al. 2021), grain filling (Yang et al. 2020) and other quantitative traits (Zhou et al. [Bibr CR100][Bibr CR99]; Zhou et al. 2021b).

HSP promoters also deserve a mention, particularly after their increasing biotechnological application as “inducible promoters” (Harrington et al. 2020; Liu et al. 2021), driving the transgene expression in a specific tissue / phenological stage, starting from a thermal stimulus. This feature is of great value in genetic engineering projects, given that the constitutive promoters can lead to undesirable phenotypes (or even embryonic lethality) due to ectopic expression. In short, the transgene inactivity maintenance under normal temperatures has the following advantages: higher rates of directed mutagenesis; improvements in the transformation frequencies on T_0_ generation; higher number of usable events; lower percentage of transgene leakage (Wang et al. [Bibr CR82][Bibr CR83]); higher rates of transmission of mutations to the progenies; and possibility of utilization even on CRISPR projects (Nandy et al. 2019).

In this study, we aimed to carry out a deep ZmHSPs' prospection, categorization and validation, followed by rigorous in silico and in vitro analyses, in order to obtain relevant data about their sequences, structures, genomic distribution, expression profiles, gene ontology and phylogeny, valuable enough to subsidize future projects, mainly concerning genetic engineering on maize.

## Results

### Organization of all the prospected data

***General overview of "******Organization of all the prospected data******" section:*** 1041 accession numbers were collected in total. After eliminating redundancies, 313 different proteins were cataloged. After analyzing their sequences, we validated 182 maize heat-shock proteins. Tables about all of them have been plotted in the supplementary material, in order to establish an extensive database about them.

**Fig. 1:**
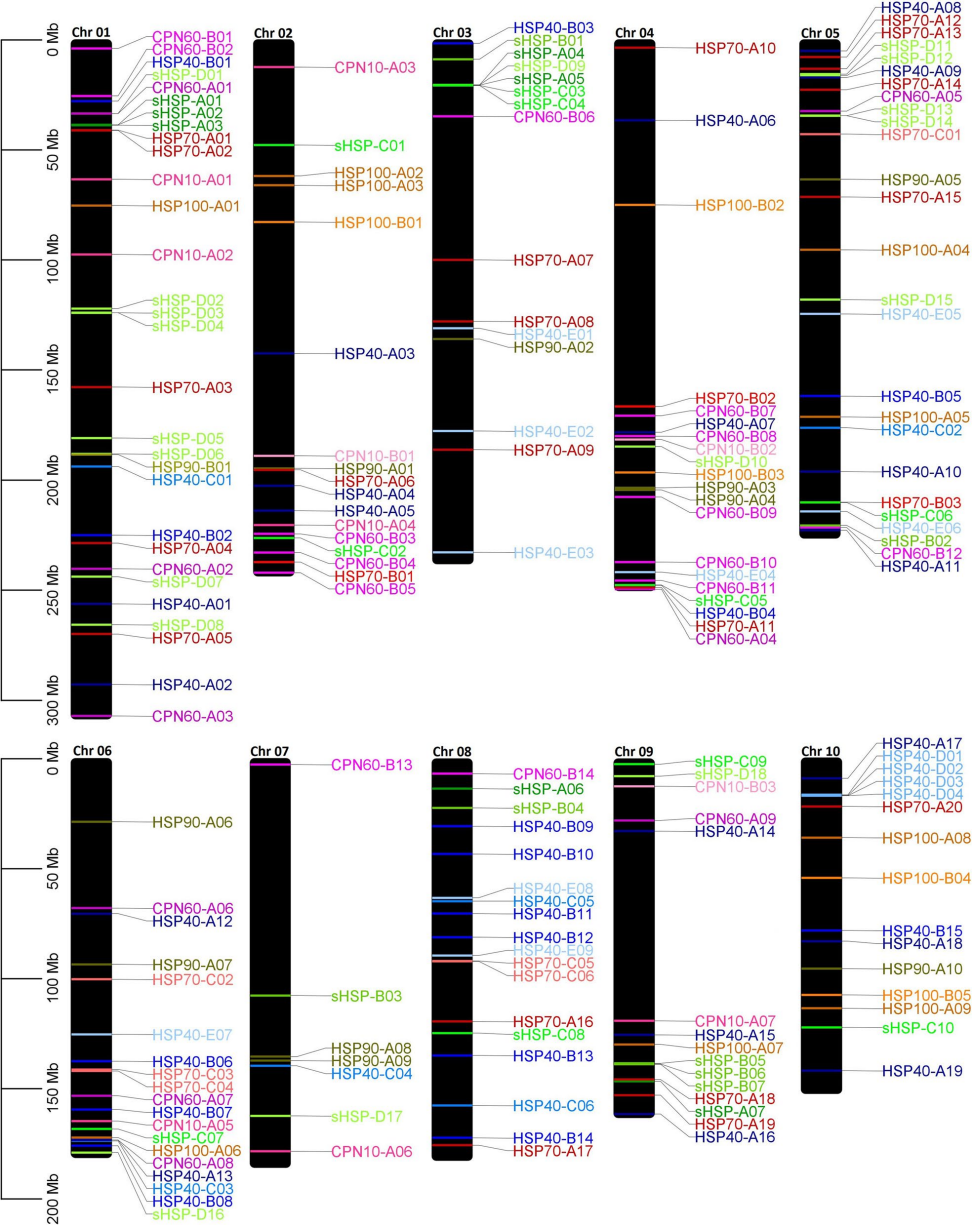
Chromosomal map with localization of the 182 validated ZmHSPs' coding genes. *Annotations:* the 182 ZmHSPs are distributed across all the ten maize chromosomes (B73 reference genome). The genes listed below are identified according to the nomenclature suggested by us. In the Additional File 1 (more precisely, at the Table S3), this nomenclature can be correlated with the one adopted in the most recent assembly version of the B73 maize genome (Zm-B73-REF.-NAM-5.0). At the Table S4 and Table S5 (present in the Additional File 1 as well), this correlation can also be made with older names, mentioned in several papers and databases

At first, 1041 ZmHSPs accession numbers, all of them hitherto arbitrary, were raised (Additional file 1: Table S1). Among the three approaches adopted, the search using profile hidden Markov models (HMMs) proved to be the broadest, generating 646 accession suggestions. However, at that point we had already faced lots of tautology (same protein being cataloged with different names), laconism (only generic presentation of the nomenclature) and disorder cases (same accession number being used to designate proteins whose coding genes are at different *loci*). The clustering process was quite useful, but insufficient; so, a considerable organization portion had to be carried out “manually”, through numerous BLASTs between the biological sequences. Once this process was over, we counted 313 putative HSPs in the B73 maize genome (Additional file 1: Table S2). Nevertheless, not all of them obeyed the premises inherent to each family, particularly regarding the MW and/or the presence of typical conserved domains. Thus, after a new and meticulous filtering, argued below (in the next section), 182 “validated” ZmHSPs were left, which are distributed by the ten maize chromosomes (Fig. 1).

On the Additional File 1, besides the total accession numbers (Table S1) and the list of the 313 putative ZmHSPs, with no tautologies/redundancies (Table S2), we organized tables about: the several nomenclatures for each protein, adopted on the various consulted databases (Table S3, S4 and S5); a new nomenclature, proposed by us, based on family, class and chromosomal location (Table S3); the possible orthologous genes, present in model plants and related species (Table S6 and S7); the motifs identified in each family (Table S8); the codes related to the conserved domains to which such motifs would belong (Table S9); and the ZmHSPs that had their expression levels significantly altered after exposure to AbSts (Table S10). Furthermore, we made available, on the Additional File 2, the spreadsheets referring to data about subcellular localization (Sup. Spr. 1), cellular components (Sup. Spr. 2), biological processes (Sup. Spr. 3), molecular functions (Sup. Spr. 4) and expression patterns (Sup. Spr. 5) of each ZmHSP we have identified / validated, a set of information that subsidized the in silico analyses. Finally, the Additional File 3 reports data from the in vitro analyses of gene expression, which we have performed on tropical maize genotypes.

### Identification of conserved domains and subcategorization into classes

***General overview of "******Identification of conserved domains and subcategorization into classes******" section:*** The 182 maize heat-shock proteins identified belong to seven different families: there are 14 ZmHSP100s/CLPs, 11 ZmHSP90s, 29 ZmHSP70s, 23 chaperonins of 60 kDa (ZmCPN60s), 10 chaperonins of 10 kDa (ZmCPN10s), 53 ZmHSP40s/DNAJs and 42 ZmSmallHSPs. Each ZmHSP family was divided into 2 to 5 classes (subfamilies) according to the presence of certain conserved domains (CD). Several motifs, present in certain subfamilies, exhibit some similarity with proteins from other families (even from different kingdoms), a fact that could help to explain some mechanisms of action still not understood regarding the HSPs. In this section, the results are divided by ZmHSP family:

#### ZmHSP100s/CLPs

Five motifs were identified: the first and the third ones are closely related to the AAA+ ATPases superfamily, which participate in several cellular processes in organisms from all kingdoms. Although one group acts in replication, recombination, DNA repair and transcription, another group (the “classical AAA”) is involved in protein structuring, facilitating folding and unfolding, assembly or disassembly of protein complexes, in addition to their transport and degradation. Our analyses revealed a certain similarity of these sequences with those of the Sigma-54 factor interaction domain, which plays a specific role in bacterial stress responses (Danson et al. 2019). Motifs 2 and 4, besides exhibiting the AAA proteins-related domain, showed another one, typical of CLPB chaperonins. We also noticed some similarity to: bacterial TNIBs, binding proteins involved in heavy metals resistance, encoded by genes in transposons; and to the E protein associated with bacterial virulence, so studied in *Agrobacterium tumefaciens* due to its ability to bind ssDNA (Lapham et al. 2021). Motif 5, in turn, is related to types-R and -N CLPs. So, we subdivided the fourteen ZmHSP100s into two classes: A, spanning nine proteins having all the five motifs, with two copies of the fifth one (located at the N-terminal); and B, with five proteins having, in addition to a motif 5 single copy, only those regions concerning the AAA+ ATPases domain (Additional file 1: Figure S1).

#### ZmHSP90s

The eight motifs are directly associated with the HSP90s' family: being related to HSP90s-like ATPases; or to regulators / mediators of molecular junctions; or even to proteins involved in ATPases' ABC-mediated conformational changes of the invasive elements, which culminates in the splitting / activation of specific effectors under biotic stress conditions (Krishnan et al. 2020). The eleven ZmHSP90 sequences show a highly conserved pattern: ten of them (those classified as A) display the eight motifs in a same order; the only exception (class B) does not have the motifs 1, 2 and 6, which result in a lower MW (40,6 kDa), giving it an appearance of “incomplete”, perhaps due to some arbitrariness during its ORF's inference (Additional file 1: Figure S2).

#### ZmHSP70s

All the seven identified motifs are closely related to the HSP70s' family. Few of them (motifs 1 and 2) show some similarity with: the MRE-B/MBL proteins, which determine bacterial cells shape (and, due to their likeness with actin and tubulin, may explain the evolutionary origin of eukaryotic cytoskeleton constituents); and also with the nucleoporins GLE1, which act in the mature RNA exportation to cytoplasm, beyond in the initiation of translation processes by themselves. ZmHSP70s have a very conserved region (motifs 2, 1, 6, 7 and 5, arranged in a row): those twenty proteins showing motifs 4 (upstream this region) and 3 (downstream) were cataloged in the class A; the three ones having only motif 4 were classified as B, while those six without both, possessing just that more conserved "central region", were put in C-class. B and C classes’ proteins exhibit higher MW (80–100 kDa) (Additional file 1: Figure S3).

#### ZmCPN60s

We basically found domains pertinent to TCP-1/CPN60 family records (occasionally denominated GroEL/TCP-1), some of which also related to the following proteins: in motif 1, TOPRIM, a catalytic domain involved in DNA strand breakage and rebinding; in motif 3, RFC, from the clamp loader complex (ring-shaped proteins that surround the DNA, conferring high processability to DNA polymerases); and AbiEi, which acts on a prokaryotic plasmids' stabilization mechanism known as the “toxin-antitoxin system” (able to respond to stresses, interrupt cell cycle and induce programmed cell death) (Hampton et al. 2018). Motifs 5 and 2 are present in all the twenty-three ZmCPN60s, which were classified into two groups: A, with nine proteins having motifs 1 and 4 in the most central portion; and B, composed by fourteen with motif 3, close to C-terminus (Additional file 1: Figure S4).

#### ZmCPN10s

The three identified motifs concern the chaperonin 10 kDa subunit family, being always arranged in the same order (1–2-3). The difference is that in seven ZmCPN10s (those put on the class A) there is just one copy of this segment, while in three others (class B) two copies in tandem are present at the same protein (Additional file 1: Figure S5).

#### ZmHSP40s/DNAJs

In this family, which is the most heterogeneous among all ZmHSPs, eight motifs were determinant: the second and third regard to the J-domain's N-terminal, with certain similarity to the SLATT proteins, which contain two transmembrane helices and act as pores-forming effectors during situations of cell conflict that depend on the production of secondary messenger nucleotides (key components of signal transduction networks, linking the sensorial input with the output regulatory responses). At the other extreme, motifs 6, 1 and 5 are inherent to J-domain's C-terminal. In the ZmHSP40s' central portion, two motifs stand out: the eighth, proper of the J-domain's X region; and the seventh, from a region where the sequence CXXCXGXG is typically found, similar to Anti-TRAP proteins (which, in prokaryotes, controls the operons transcription, regulating the tryptophan association to tRNA) and to HypA (a metallochaperone that binds nickel, conducting it to enzymes as urease and NiFe hydrogenases) (Higgins 2019). Finally, motif 4 exhibits a domain representative of the indoleamine 2,3-dioxygenase, the first enzyme in tryptophan catabolism through the kynurenine pathway, being responsible for its depletion during immunological subversion. Let us remember that tryptophan is essential in different regulatory pathways during plant growth, both as a common precursor and as a regulatory inductive signal (Forsyth et al. 2020), serving also like a “crossing point” with auxin biosynthesis, thus mediating the transitory growth (Erland & Saxena 2019). We have also noticed that it is relatively common ZmHSP40s being downstream of thioredoxins genes or, sometimes, show any CD typical from these small redox proteins, which act in the ROS response mechanisms, as well as in the cell-to-cell communication, regulating a wide spectrum of critical functions (photosynthesis, flowering, seed development and germination, among others). All this opens up possibilities for many speculations about ZmHSP40s' action means during response mechanisms to AbSts in plants, particularly in signal transduction pathways. Anyway, we cataloged them into 5 classes: A, with nineteen proteins preserving the central region, with the CXXCXGXG standard sequence; B, with fifteen holding just the five motifs pertaining to J-domain's N- and C-terminals' regions; C, with six representatives displaying the J-domain's X region, in addition to its N-terminus; D, which has the extensive motif 4 just downstream of N-terminal region (it is only present in the four tandem-arranged J-like proteins); and E, with nine ZmHSP40s exhibiting only the two motifs from J-domain's N-terminal region (Additional file 1: Figure S6).

#### ZmSmallHSPs

Motifs 2, 3, 8 and 1 (in that order) are inherent to the HSP20-related domains, being present in all the maize SmallHSPs, albeit, sometimes, in an apparently incomplete way. The other ones were observed only in some classes: motif 4 is just upstream to HSP20 domain; motifs 5 and 6 are at N-terminus; motif 7 is at C-terminus. So, the classification looked like this: class A, with seven ZmSmallHSPs containing the 5–4–2–3–8–1–7 motifs; B, with seven proteins displaying the 6–4–2–3–8–1–7 ones; C, ten SmallHSPs with just 4–2–3–8–1; and D, with eighteen showing only the HSP20-domain typical motifs, with no other region conserved up or downstream (Additional file 1: Figure S7). At the end, we noticed certain similarity between the ZmSmallHSPs' sequences and some well-known domains: CS, a protein–protein interaction module usually found fused with other domains; GRAS, present in TFs involved on gibberellin signaling and in other proteins capable of binding and modifying small molecules; L6, from ribosomal proteins that interact with RNAs and other proteins; BOM, typical of OSM-Ys, periplasmic proteins expressed in response to different stress types, mainly the osmotic ones (Bryant et al. 2020); FKBP26, an “exceptionally flexible” multidomain chaperone, with non-specific mechanism for substrate recognition, which allows it to aggregate to a wide range of proteins; and GvpH, which forms intracellular gaseous vesicles, something quite advantageous for maintaining the cell turgor under adverse conditions (Völkner et al. 2020). Although these last four cited domains are typical from bacterial proteins, such information helps us to infer about the ZmSmallHSPs action mode in face of stress situations to which plants are constantly exposed.

### Subcellular localization

***General overview of "******Subcellular localization******" section:*** The 182 validated ZmHSPs could now have their cellular localization predicted. For that, we brought together the Gene Ontology and DeepLoc software algorithms, associating both the individualized prediction (i.e. of each ZmHSP, considered separately) and the one made after their categorization into classes / subfamilies (according to their structural similarities). It is noteworthy that such categorization becomes very useful to visualize the differentiation that exists between each class (even within the same family) in terms of the cellular components in which those protein sets would act.

**Fig. 2:**
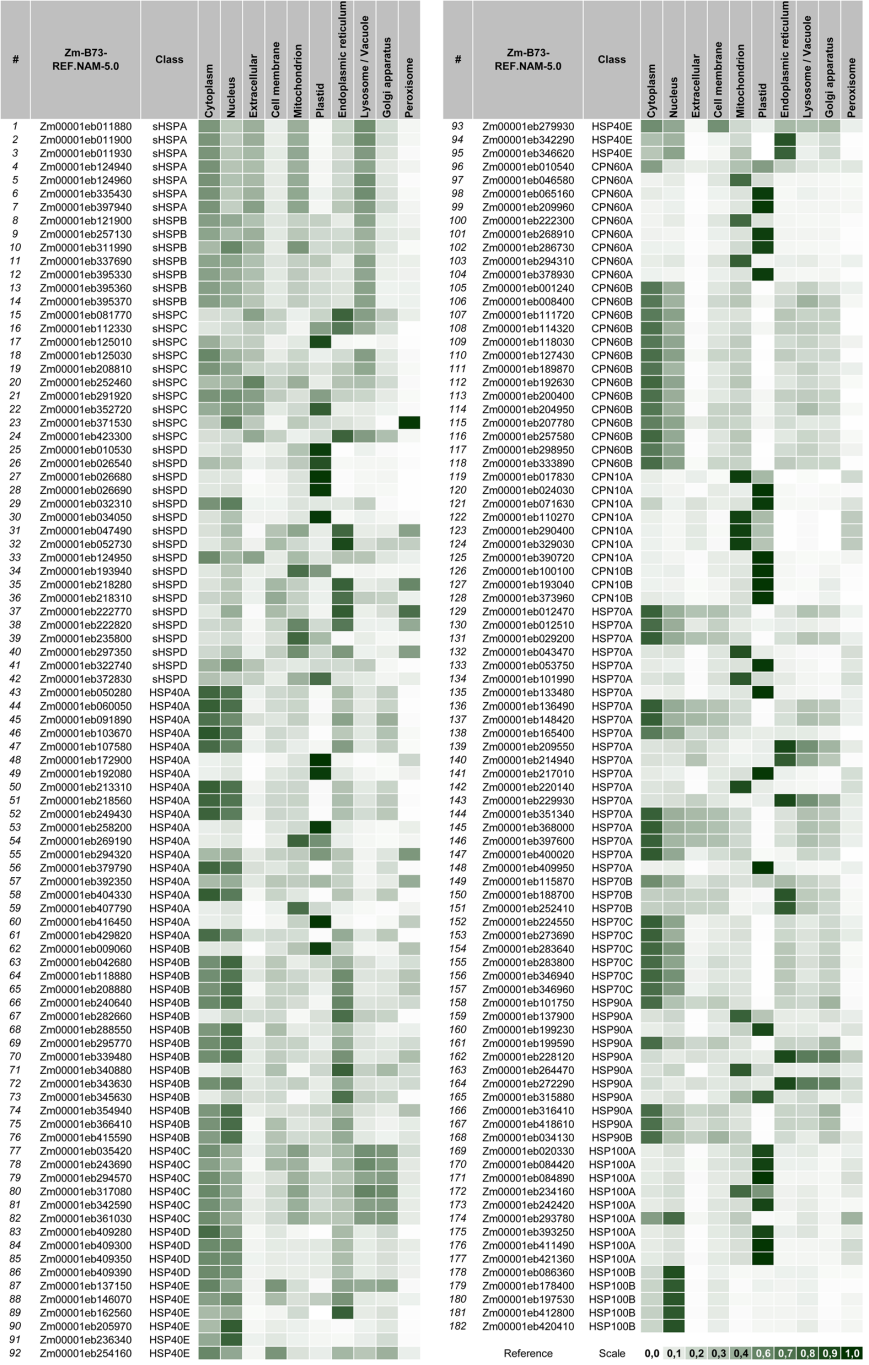
Subcellular localization prediction in line with the DeepLoc tool. *Annotations:* this heatmap indicates the probability (on a 0 to 1 scale, i.e. from 0 to 100%) of the corresponding proteins (cited at the horizontal lines, according to the official Zm-B73-REF.-NAM-5.0 nomenclature) being located in that respective organelle (detailed at the top of the table). The color range adopted (on a white-to-dark green scale) is illustrated in a caption at the bottom right. The data that gave rise to this heatmap are written in Additional File 2 (at the Sup. Spr. 01—Subcellular Localization)

**Fig. 3:**
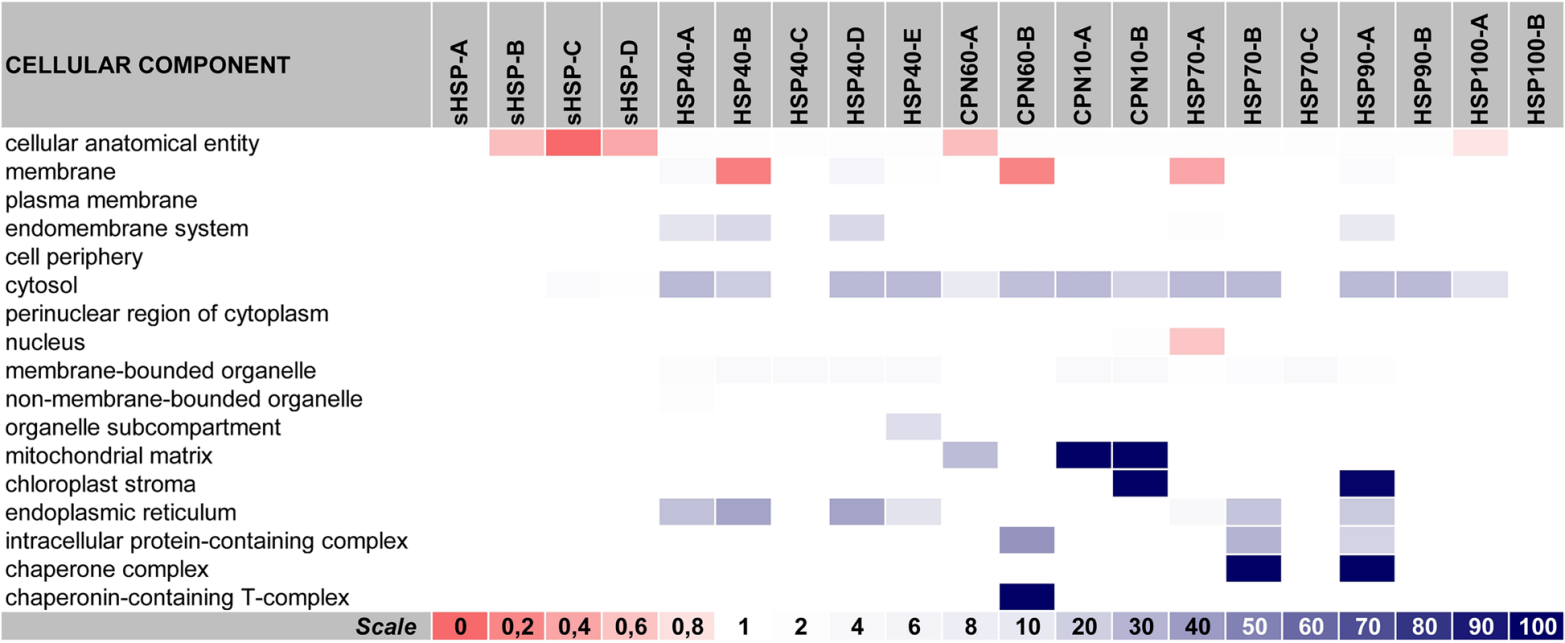
Cellular components in which the ZmHSPs classes (subfamilies) probably act, according to the gene ontology concepts. *Annotations:* this heatmap indicates the fold enrichment (FE, on a 0 to 100 + scale) of each ZmHSP class with regard to seventeen categories. The categories are listed in the horizontal lines, while the columns represent each ZmHSP class (subfamily). FE > 1 indicates over-representation of the respective category: for that cellular component, the number of genes in the list once analyzed (i.e. the genes belonging to that class / subfamily) is greater than the Expected Value (which is calculated based on the Reference List from the PANTHER classification system). Conversely, FE < 1 indicates under-representation. The data that gave rise to this heatmap are written in Additional File 2 (at the Sup. Spr. 2—Cellular Components). The color range adopted (on a red-to-blue scale) is illustrated in a caption at the bottom of the figure

It was possible to confirm that: most ZmHSPs play some role in cytosol; ZmHSP100s-A are more present in plastids, while ZmHSP100s-B tend to be accumulated on nucleus; ZmHSP90s-A appear to act strongly at chloroplast stroma (CS) besides, to a lesser extent, in the endoplasmic reticulum (ER), mitochondria and endomembrane system (EMS); ZmHSP70s-A and B-classes act in ER too, particularly on the cisternal space chaperone complexes, while ZmHSP70s-C are more active on cytoplasm and nucleus; ZmCPN60s-A are located basically in mitochondria and plastids, while ZmCPN60s-B on nucleus and cytoplasm, operating at the TCP-1 chaperone complexes (Gestaut et al. 2019); ZmCPN10s are prominent at the mitochondrial matrix and CS (Zhao & Liu 2018); ZmHSP40s (the most heterogeneous HSP family) can be found in several organelles, but mainly at ER, EMS and nucleus; ZmSmallHSPs do not have a “defined place” to act, given that they perform their functions in different intracellular compartments (Janowska et al. 2019) (Figs. 2 and 3).

### Biological processes

***General overview of "******Biological processes ******" section:*** Using the same tactics of segregation into families and subfamilies (classes), the prediction work was now concerned with the biological processes in which each class of ZmHSPs would be involved. Indeed, the aforementioned categorization may facilitate an attempt to unravel some metabolic pathways in which ZmHSPs apparently act, given that the amino acid composition patterns once detected seems to have influenced such performances.

Firstly, we realized the ZmHSPs' importance in several protein structuring processes: ZmSmallHSPs on oligomeric complexes formation (Waters & Vierling 2020); ZmHSP70s acting on unfolded proteins, remodeling native oligomers (as well as other stable aggregates) through entropic traction, with ATP expenditure; and the role of ZmHSP40s, chaperonins, ZmHSP70s and ZmHSP100s in protein folding. The ZmHSP70s further proved to be crucial for protein structuration, notedly during cellular responses to malformed proteins, topologically incorrect. In addition, it is notorious their participation during the regulation of intracellular signals' transduction (particularly in the apoptotic signaling pathway, which they negatively regulates), even in situations of hypoxia (Zhai et al. 2019) and/or exposure to other stresses sources (Aghaie & Tafreshi 2020).

**Fig. 4:**
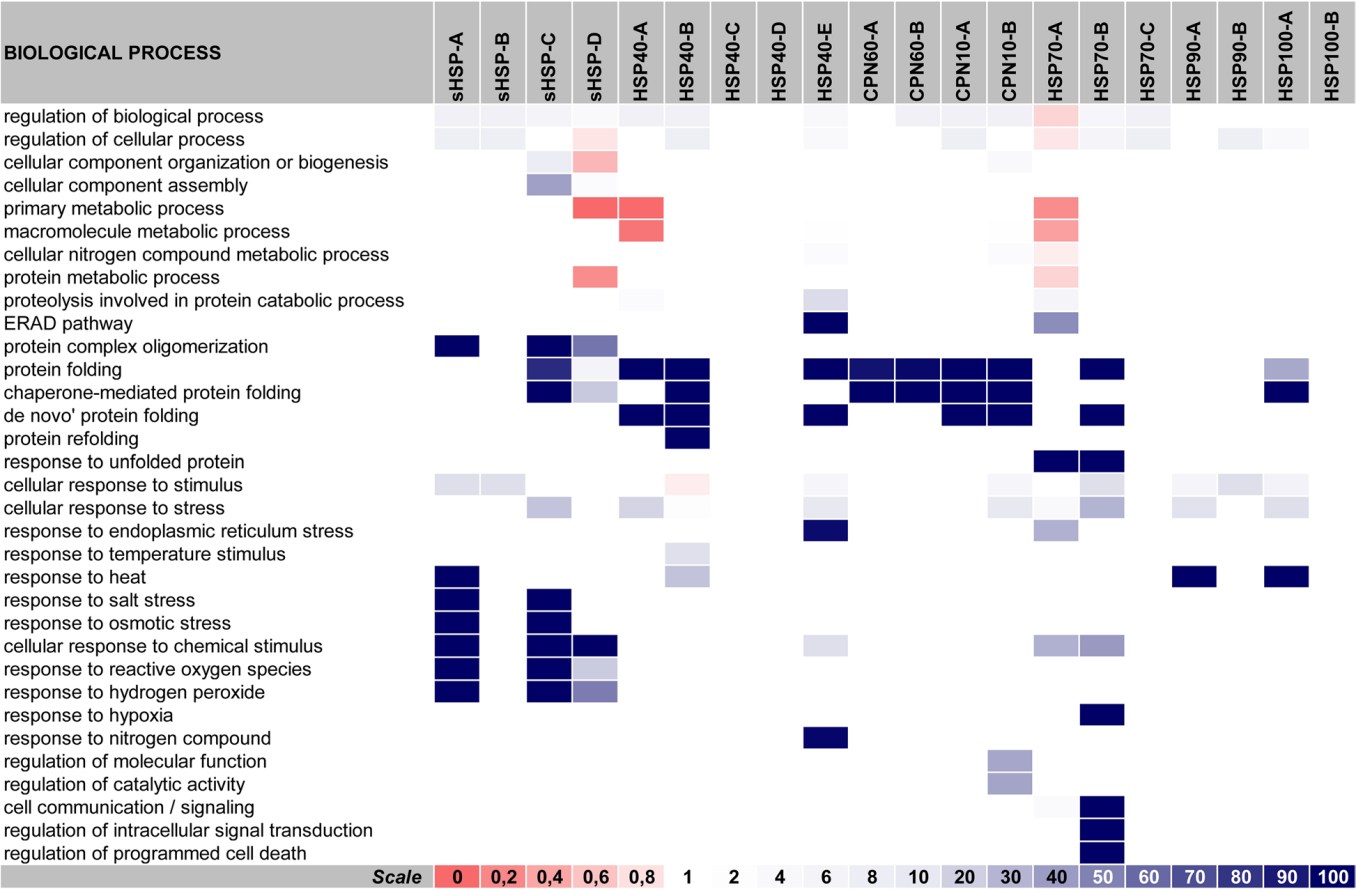
Biological processes in which the ZmHSPs classes (subfamilies) would be involved, according to the gene ontology concepts. *Annotations:* this heatmap indicates the fold enrichment (FE, on a 0 to 100 + scale) of each ZmHSP class with regard to thirty-three categories. The categories are listed in the horizontal lines, while the columns represent each ZmHSP class (subfamily). FE > 1 indicates over-representation of the respective category: for that biological process, the number of genes in the list once analyzed (i.e. the genes belonging to that class / subfamily) is greater than the Expected Value (which is calculated based on the Reference List from the PANTHER classification system). Conversely, FE < 1 indicates under-representation. The data that gave rise to this heatmap are written in Additional File 2 (at the Sup. Spr. 3—Biological Processes). The color range adopted (on a red-to-blue scale) is illustrated in a caption at the bottom of the figure

We still apprehended the ZmSmallHSPs' essential role (mainly those from the A and C-classes) in the response mechanisms to several AbSts' sources, like heat, salinity, ROS and hydrogen peroxide. Analyzing exclusively high temperatures, we also must highlight the ZmHSP90s-A and ZmHSP100s-A again. Regarding the chaperonins, while the ZmCPN60s operate almost exclusively on proteins folding, the ZmCPN10s, in turn, participate in the regulation of important enzymes from the antioxidant system (such as superoxide dismutase and oxidoreductases in general, like catalase, peroxidases and others) (Rajput et al. 2021), besides acting on the proteins' three-dimensional arrangement, mostly those dependent on chaperone complexes (Horwich & Fenton 2020) (Fig. 4).

It is interesting see that “the less complete” subfamilies (e.g. ZmHSP40s-C and D-class, ZmHSP70s-C and ZmHSP100s-B) did not stand out (in terms of biological relevance), strengthening the importance of each conserved domain (in every ZmHSP family and subfamily) for the function performances (Fig. 4).

### Molecular functions

***General overview of "******Molecular functions******" section:*** Complementing the set of information from the previous section, we are now able to predict the molecular functions of each ZmHSPs class (subfamily). Once again, the conserved domains (the main parameter for our previous categorization into classes) were fundamental for making inferences about an eventual functional parity between proteins of the same class. Compiled to what has already been presented formerly, these results help us to elucidate the modus operandi of ZmHSPs on the mechanisms of response to abiotic stresses in maize.

**Fig. 5:**
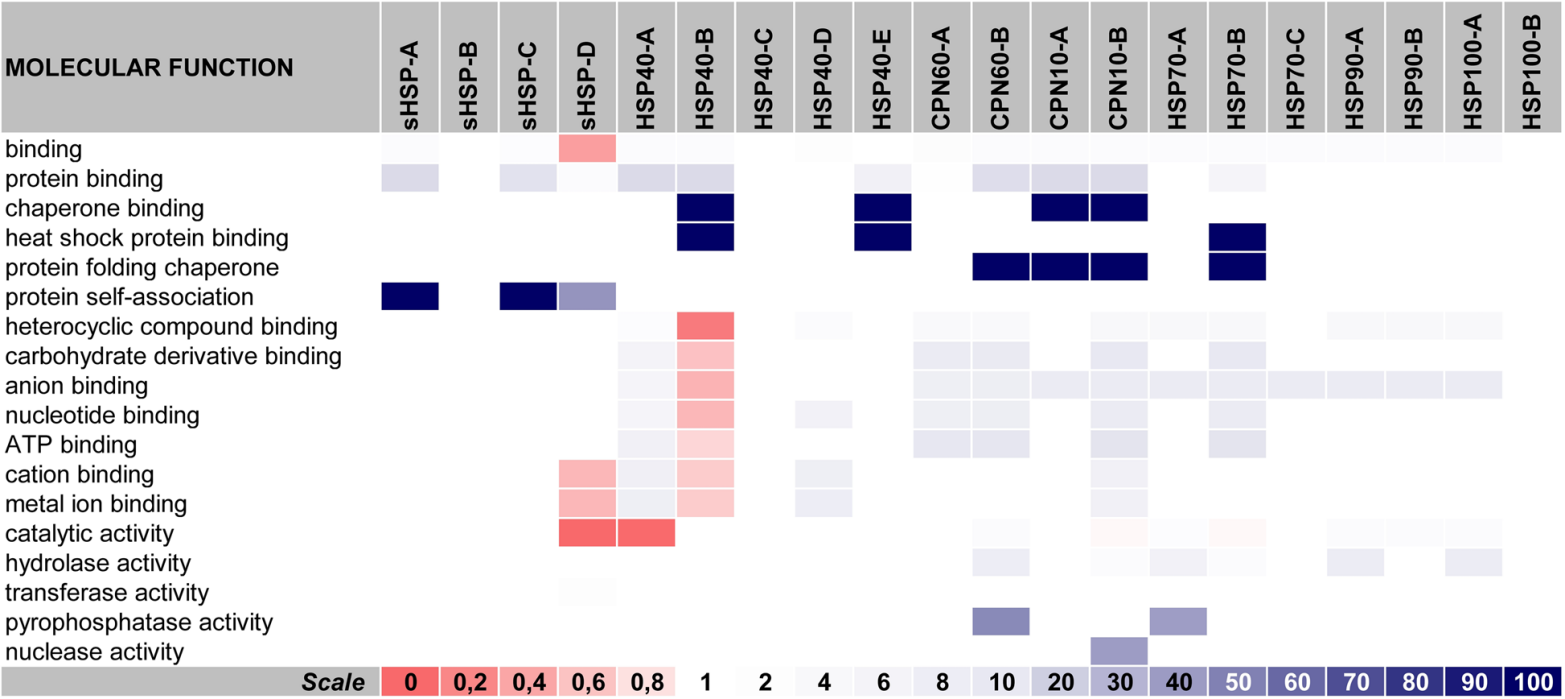
Molecular functions that ZmHSPs classes (subfamilies) apparently perform. *Annotations:* this heatmap indicates the fold enrichment (FE, on a 0 to 100 + scale) of each ZmHSP class with regard to eighteen categories. The categories are listed in the horizontal lines, while the columns represent each ZmHSP class (subfamily). FE > 1 indicates over-representation of the respective category: for that molecular function, the number of genes in the list once analyzed (i.e. the genes belonging to that class / subfamily) is greater than the Expected Value (which is calculated based on the Reference List from the PANTHER classification system). Conversely, FE < 1 indicates under-representation. The data that gave rise to this heatmap are written in Additional File 2 (at the Sup. Spr. 4—Molecular Functions). The color range adopted (on a red-to-blue scale) is illustrated in a caption at the bottom of the figure

At that point, it was still possible to confirm: the ZmSmallHSPs' association with other proteins (Collier & Benesch 2020); the ZmHSP40s' cooptation as co-chaperones at the HSP70 machinery, serving as functional specificity boosters during this “system for quality control of proteins”; and the outstanding participation of ZmHSP70s-B and chaperonins on protein folding through a chaperone action (Genest et al. 2019; Horwich & Fenton 2020), with the difference that ZmHSP70s' expression is stimulated by thermal shock, while ZmCPNs seem to be more ubiquitous. Incidentally, both, as well as the ZmHSP90s, convert the ATP-derived energy into stabilization of the target proteins (Goloubinoff et al. 2018) (Fig. 5).

### Individualized expression profiles

***General overview of "******Individual expression profiles******" section:*** Performing a joint analysis, with data collected soon after diverse maize tissues have been subjected to different sources of abiotic stress, we were able to infer which genes apparently stand out (in terms of multiplication on their expression levels) in that "universe" of 182 previously validated ZmHSPs. These genes (about 3 dozen, listed in Additional file 1: Table S10) are therefore promising for future projects of genetic engineering (particularly for gene overexpression) aimed to design superior genotypes, potentially tolerant to the aforementioned abiotic stresses.

**Fig. 6:**
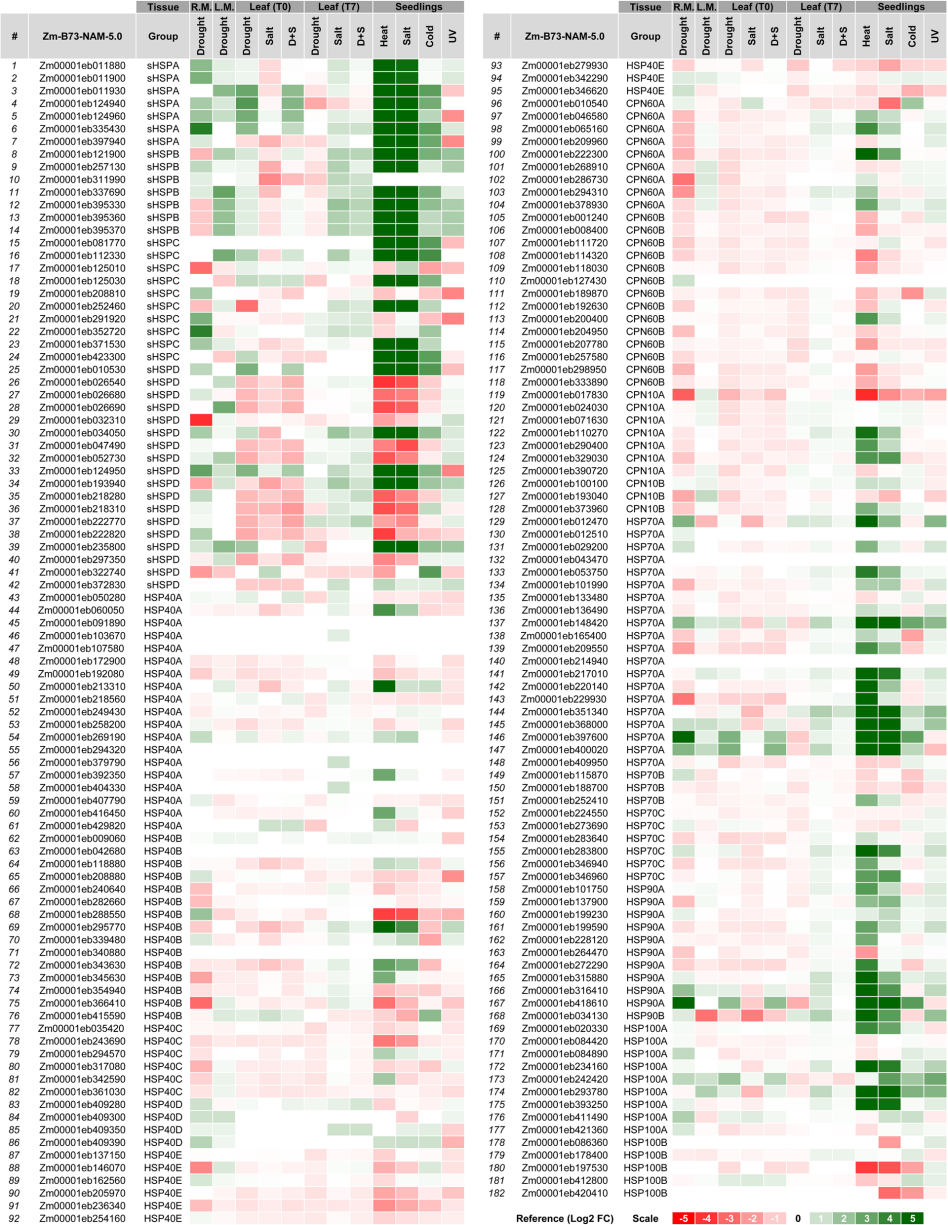
Heatmap of the 182 ZmHSPs' individualized expression profiles in several maize tissues, in terms of different AbSts. *Annotations:* it represents the fold change (FC) after comparing each gene expression level, observed in seedlings / plants exposed to the respective abiotic stresses (detailed at the top of the table), with those from the control ones, which grew in optimal situation (i.e. with no stress). About the tissues above described: “R.M.” means reproductive tissue (at this case, the fertilized ovaries); “L.M.” means leaf meristem; “T0” means leaves from recently stressed plants; “T7” means leaves from plants that were kept irrigated for seven days after the stress exposure. About the abiotic stresses (AbSts), “D + S” means drought plus excessive salinity. The results were plotted on a Log2 FC ratio, a binary logarithm scale to those fold change values. The color range adopted (on a red-to-green scale) is illustrated in a caption at the bottom right. The data that gave rise to this heatmap are written in Additional File 2 (at the Sup. Spr. 5—Expression Patterns)

In maize seedlings, most ZmSmallHSPs and ZmHSP70s had their expression levels multiplied hundreds or thousands of times after exposure to excessive heat. In other families, just one ZmHSP90 and one ZmHSP100 stood out. Faced with saline stress, the behavior was repeated, but on a somewhat lower scale. Then when the seedlings were subjected to cold, once again a relevant expression increase was noted, although relatively smaller (5–10 times). As for UV ray exposure, the increase occurred just in some of those ZmHSPs. Studying singly the drought effects, some ZmSmallHSPs and ZmHSP70s had their expression levels increased 5–10 times in leaves and/or leaf meristems, a behavior that was not repeated when the plants had a week of adequate irrigation to recover themselves. When fertilized ovaries were the evaluated tissue, we saw a much more significant increase: two ZmSmallHSPs, one ZmHSP70 and one ZmHSP90 were 18–50 times more expressed. When drought was combined with salt excess, the results were similar; but when saline stress was applied alone, again the expression patterns change was thinner (Fig. 6, Additional file 1: Table S10).

By analyzing the gene expression data that we ourselves generated, after submitting seeds of four tropical maize inbred lines to two different drying temperatures (one of them being much higher, 50 °C), we noticed that such abiotic stress caused some ZmHSPs to be significantly upregulated. Some of these genes (one ZmSmallHSP-A, one ZmHSP90-A and one ZmHSP100-A), which had already stood out during the in silico analyses, showed a greater expression increase in genotypes tolerant to high drying temperatures. Meanwhile, others (two ZmSmallHSPs-D and one ZmHSP70-A) were upregulated in the four maize inbred lines once evaluated (two tolerant and two non-tolerant) (Additional file 1: Figure S8). By the way, examining the Additional File 1 more thoroughly, we will see that Table S10 highlights promising ZmHSP genes for future projects of genetic engineering, information that can support the development of new transgenic materials, where such genes may be overexpressed.

## Discussion

### Organization of prospected data

The duplicate records, i.e. tautology / redundancy cases detected during primary organization of accession numbers, as well as other disorder situations (including laconism), are usually occasioned from two kinds of situations: a) lack of a “basic standardization” regarding specific genetic annotation (Tello-Ruiz et al. 2019); b) the existence of overlapping genes (OLGs), also called “double coding regions”, which can arise in four ways: from nearby genes, sometimes even juxtaposed; from alternative splicing events of the same gene; from different translation initiation sites of a single transcript; from conserved non-coding regions (SNCs) evolution.

### ZmHSPs chromosomal positions

Observing the chromosomal map (Fig. 1), we can conjecture about probable occurrences of gene duplications, for example, on chromosomes 1 (three ZmSmallHSPs-A), 9 (three ZmSmallHSPs-B) and 10 (four ZmHSPs40s-D), among other possible ones, where *loci* arranged in tandem share high percentages of their respective genomic sequences (Additional file 1: Table S11). Another curiosity is the fact that chaperonins coding genes (ZmCPN60s and ZmCPN10s) are mostly located at the peripheral regions of chromosomes, near the telomeres. In eukaryotes, telomeres are maintained by telomerase ribonucleoprotein (RNP), whose biogenesis pathway is quite complex, with two crucial steps: assembly of the TERT and TERC components into the active telomerase enzyme; and its location on the Cajal bodies, for its subsequent recruitment at telomeres (Viviescas et al. 2019). Both are dependent on the interaction of TERC with cofactor TCAB1, whose folding is carried out precisely by the TRiC/CCT complex, the chaperonins' group II. Therefore, it is quite possible that, during the genomic evolution continuous process, which happens rapidly in the subtelomeric regions (since they are subject to meiotic recombination higher levels), there has been a certain selection in this sense (“strategic location” of the chaperonin genes), given that, although the dynamic nature of these regions provides a functionally useful variation for genetic diversification, it also results in a mechanism that can lead to speciation.

### Expression profiles

**Fig. 7:**
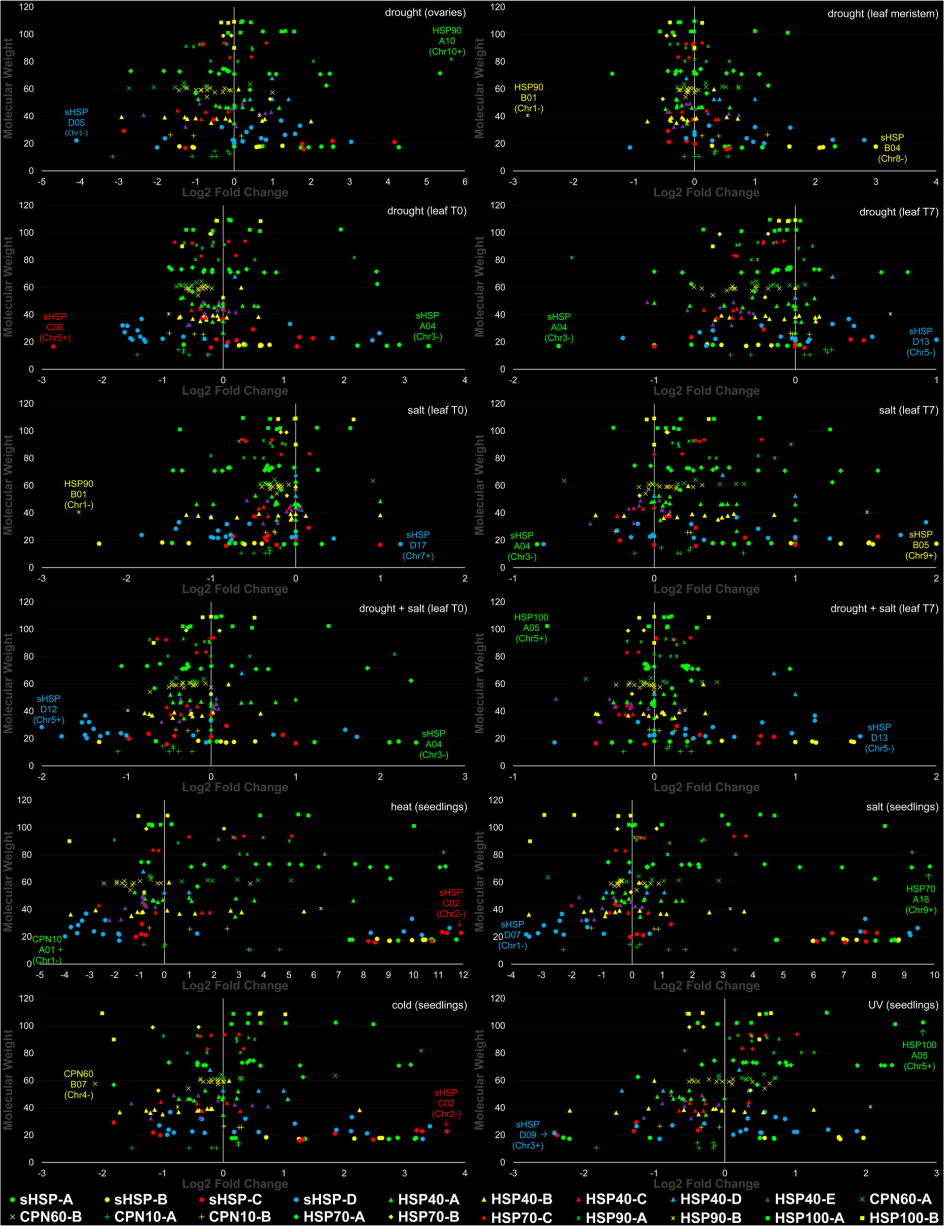
Scatter plot of the expression profiles for each one of the 182 validated ZmHSPs, according to different AbSts' types and maize tissues. *Annotations:* on the x axes of each graph, the MWs (in kDa) of ZmHSPs are plotted; on the y-axis, the fold change (of their expression levels) in a binary logarithm (log2 n) scale. The geometric shapes (caption at the bottom of figure) represent the families (spheres for ZmSmallHSPs, triangles for ZmHSP40s, Xs for ZmCPN60s, crosses for ZmCPN10s, diamonds for ZmHSP70s, asterisks for ZmHSP90s, squares for ZmHSP100s), while the colors designate the proposed classes / subfamilies (green for A, yellow for B, red for C, blue for D, purple for E). The data that gave rise to this scatter plot are detailed in Additional File 2 (at the Sup. Spr. 5—Expression Patterns)

Individualized results (regarding expression levels observed after exposure to several AbSts) have imputed, on those ZmHSP genes that stood out (Additional file 1: Table S10, Fig. 6), a great potential for genetic engineering in maize aiming to increase the tolerance levels. When we deal with such expression profiles after the ZmHSPs have been divided into the classes proposed by us (Fig. 7), we had the following:

#### Drought

ZmHSP100s-A had their expression levels greatly increased in ovaries, which did not happen in those from B-class and/or in ZmHSP100s in general (both classes) when the analyzed tissue were the leaves. It shows us: this family merit during the reproductive stage; also, the relevance of conserved regions placed in motifs 2 and 4 (both absent in ZmHSP100s-B). Among the ZmHSP70s, highlight for B-class those, which expression basically decreased. As for chaperonins (both families, ZmCPN60s and ZmCPN10s), we noticed a strong downward trend after the exposure to drought. ZmSmallHSPs from A and B-classes (both with well-preserved regions at their extremities) exhibited a significant increase in post-drought expression levels, which did not occur in C and D-classes, leading us to speculate about the importance of the motifs 5, 6 (at N -terminal) and 7 (at C-terminal) for such performance.

#### Salinity

Many families / classes of ZmHSPs were downregulated, situation that was reversed when stressed plants were irrigated again, which coaxes us to infer that, although some researchers defend the stochastic model for gene expression during these situations, it is most likely that there really is repression resulting from osmotic stress, probably mediated by chromatin structuring (mainly by H3 histone phosphorylation). In seedlings, the results were even more incisive: the ZmHSP100s-A, the ZmHSP90s, most of the ZmHSP70s (mainly those from A-class), several chaperonins (especially the A-classes in both families, with 60 and 10 kDa) and many ZmSmallHSPs (except most of those that make up the “incomplete” class: D) exhibited very high expression levels.

#### Drought + salinity

When these two stresses were combined, the results in maize leaves were analogous: decreased expression levels in both chaperonin families, in most ZmHSP90s and ZmHSP40s, also in ZmSmallHSPs-D. In contrast, some ZmHSP100s, ZmHSP90s, ZmHSP70s and ZmSmallHSPs, all of them from A-classes (the “most complete” of each family), showed significant increments on their expression. In the three aforementioned cases (drought, salinity and their combination), the situation tended to normalize (i.e. expression levels stabilization) after stress mitigation, via subsequent irrigation.

#### Cold

ZmHSP100s had their expression levels increased. ZmHSP70s-A were upregulated, while B-class downregulated. Most of the ZmSmallHSPs exhibited increased expression, except some of C and D-classes (those with the lowest conserved regions number).

#### UV

Such exposure, in seedlings, was the one with less meaningful results. Nonetheless, ZmHSP90s and ZmHSP70s had their expression levels mostly increased. ZmHSP40s, with some exceptions, and ZmCPN10s were downregulated. ZmSmallHSPs, in turn, exhibited a very misshapen behavior; even so, C and D-classes tended towards upregulation.

#### Heat

The most impressive outcome was even after maize seedlings exposure to high temperatures. Many ZmHSPs were expressed hundreds or thousands of times more: most ZmHSP100s-A, ZmHSP90s A and B-classes, ZmHSP70s-A, ZmCPN60s-A, ZmHSP40s A and B-classes, and also ZmSmallHSPs (except some from C and D-classes) exhibited such behavior, with magnification peaks up to 3000 times.

#### High drying temperatures

Among the proteins whose expression we have analyzed in vitro, three stood out (one ZmSmallHSP, one ZmHSP90 and one ZmHSP100, all from A-classes of their respective family). It is noteworthy that these three ZmHSPs had already shown excellent results in the expression analyses performed in silico. Therefore, this type of stress may be a promising marker for future prospections of genes that confer tolerance to AbSts in general.

Lastly, we emphasized that the "most complete" ZmHSPs, i.e. those with the highest number of motifs inherent to their respective family (as a rule, categorized here as "A-classes") tend to be expressed at higher levels after exposure to AbSts (Fig. 7), leading us to intuit that the more conserved their amino acidic domains are, the better their functional performance will be (Aziz & Caetano-Anollés, 2021).

As conclusions, our results reveal that is undeniable the ZmHSPs' role on the response mechanisms to exposure towards environmental stress from different sources, to which maize plants are constantly subjected. Each ZmHSP family operates in certain subcellular compartments, fulfilling distinguishable molecular functions in the various biological processes in which they act. The ZmHSP families’ segregation into classes (subfamilies) proved to be a very useful approach for their functional analyses, which can be inferred based on the main conserved domains present in their amino acid sequences. The structural differences inherent to each class, within each family, directly imply their functions' performance, i.e., during their response mechanisms after the abiotic adversities.

## Materials and methods

### Prospecting the “arbitrary” aminoacidic sequences

This step was performed concurrently on three platforms: a) by name and species in the Heat Shock Protein Information Resource (HSPIR) (http://pdslab.biochem.iisc.ernet.in/hspir/); b) by name in the Maize Genomic and Genetic Database (MaizeGDB) (RRID:SCR_006600) (https://www.maizegdb.org/), where we also searched via BLAST, using as query sequence each family typical motifs (E-value < 10^–4^); c) and through hidden Markov model (HMMs), downloaded from the Curation & Model section of the respective family page on PFAM website (RRID:SCR_004726) (https://pfam-legacy.xfam.org/), currently under the guardianship of InterPro database (RRID:SCR_006695) (https://www.ebi.ac.uk/interpro/). The HMMs once downloaded were submitted in the HMMSearch tool (https://www.ebi.ac.uk/Tools/hmmer/search/hmmsearch) from HMMER platform (RRID:SCR_005305) (http://hmmer.org/), restricting the results according to species (maize taxonomy ID: 4577). All those newly collected data were compiled, adopting the most recent assembly version of the B73 maize genome (Zm-B73-REF.-NAM-5.0) as our benchmark.

### Clustering the “arbitrary” sequences

In order to exclude possible redundancies (regarding the accession numbers), the amino acid sequences of each ZmHSP candidate were double audited: firstly, they were clustered with the aid of the CD-HIT Suite tool (RRID:SCR_007105) (https://www.bioinformatics.org/cd-hit/), with a cut-off value = 0,99; and then, manually, comparing each sequence with the MaizeGDB database. As a rule, priority was given to sequences that would derive from the canonical transcript, except when other transcript has exhibited characteristics more inherent to that group, especially regarding MW and/or the presence of certain conserved domains (CD).

### Searching for CD

The remaining amino acid sequences (now, with the redundancies already excluded) were analyzed with the MAST tool (https://meme-suite.org/meme/doc/mast.html) from MEME Suite package (RRID:SCR_001783). The identified motifs (p-value < 10^–5^ for each position) were triply useful: for the ZmHSPs' validation (as members of their respective families); for their segregations into “classes” (within each family); and for the function's initial prediction of each one, which was assessed via Motif Search tool (https://www.genome.jp/tools/motif/) from GenomeNet (RRID:SCR_004165).

### Compiling the data

We have organized, on several tables, as much of the gathered information as possible, including: the different nomenclatures (according to each database) of each gene; the locus*'* chromosomal position; the number of transcripts; the size, in base pairs (bp), of the canonical transcript; the protein size, in AA number; the MW, in kDa; and the isoelectric point (pI). We have also prospected, in model plants and species close to maize, the potential orthologous genes.

### Analyzing in silico gene expression

Performed with the qTeller platform aid (https://qteller.maizegdb.org/), where we compared the expression levels of the validated ZmHSPs in the following situations: the effects of drought on maize reproductive tissue (ovaries) and on maize leaf meristem; the effects of drought, high salinity and both (combined) on maize plant leaves which were stressed (T0) or kept irrigated for seven days after the stress application (T7); the effects of high temperature, salinity, cold and exposure to ultraviolet rays on maize seedlings. So, we calculated the fold change (FC) after comparing the expression of each gene in seedlings / plants exposed to the respective abiotic stresses, with those that have grown in optimal situations (i.e. with no stress). A red-to-green heat map was generated, where changes in the expression profiles of each previously validated ZmHSP were illustrated, always on a Log2 FC ratio (a binary logarithm scale to those fold change values).

### Experimental validation for the expression levels under abiotic stress of some ZmHSPs

In order to evaluate the effects of another abiotic stress (high drying temperatures) on maize seeds, we carried out the following experiment: plants of four Geneseeds® Genetic Resources maize inbred lines, previously classified according their tolerance to high temperatures during seed drying, have been grown. The ears were harvested when the seeds had approximately 30% water content. Then, they were subjected to artificial drying: at a “normal condition” (35 °C); or under high temperature (50 °C), until the seed lots reached 13% moisture. The drying system consisted of small-scale stationary dryers, fixed-layer ("bed") model. Each dryer was composed of four removable trays, with a perforated bottom and square sections. (60 cm sides, 20 cm depth). Mercury thermometers were placed in the middle of the maize ears mass, as an additional (and independent) temperature control. The airflow was constant: 34 m^3.^ min^−1.^ ton^−1^. Soon after this process, the maize ears were manually husked. Germination essays and vigor tests ("first seedling count", "accelerated aging" and "cold test") were adopted to confirm the classification (tolerant or non-tolerant) of those maize inbred lines. A portion of seeds from each one of the eight treatments (four inbred lines x two drying temperatures) were macerated. From these materials, we extracted the mRNAs and, from them, we synthesized the cDNAs. Finally, we performed the qPCR analyses, aiming to compare the expression levels of seven maize HSPs genes (three ZmSmallHSPs, two ZmHSP70s, one ZmHSP90 and one ZmHSP100). The list of primers used, as well as the identification of the target and the reference (normalizers) genes adopted in this experiment, are located in the Additional File 1: Table S12.

### Analyzing the RNA enrichment levels

Using the Gene Ontology (GO) knowledgebase (RRID:SCR_002811) (http://geneontology.org/), in conjunction with the PANTHER classification system (RRID:SCR_004869) (http://www.pantherdb.org/), just after having segregated all the validated ZmHSPs into families and classes (subfamilies), we plotted the significantly over (or under) represented gene classes according to three “sets of categories” (biological process, molecular function and cellular component). The Expected Value represents the percentage of genes, out of the total number of genes identified in a given genome (in our case, maize), that are related to the category in question. The software algorithm has calculated the percentage of genes, among the total that we were analyzing (for each case, the gene number of the respective class / subfamily), that are related to that same category. The Fold Enrichment (FE) basically represents the ratio between these two percentages. The test's significance was determined based on the concept of “false discovery rate” (FDR, the estimated probability that the normalized enrichment score represents a false positive finding), as calculated by the Benjamini–Hochberg procedure. Only results with FDR-adjusted p-value (probability of the number of genes observed in that respective category has occurred by chance) < 0,05 (as determined by Fisher’s exact test or Binomial statistic) were considered. The results were set up in a red-to-blue heatmap format, obeying a 0 to 100 + scale regarding the fold enrichment of each ZmHSP class (subfamily) for each category.

### Subcellular localization prediction

Made by DeepLoc 2.0 tool (https://services.healthtech.dtu.dk/service.php?DeepLoc-1.0). Through a recurrent neural network that processes the entire protein sequence, with a special attention to some "key regions", the subcellular localization of each validated ZmHSP was forecasted in terms of occurrence probability (%), whose values (in a 0,0 to 1,0 scale) were plotted on a white-to-dark green heat-map.

### Outlining chromosome map and phylogenetic evolutionary tree

Performed through the TBtools (RRID:SCR_023018) (https://github.com/CJ-Chen/TBtools/releases) and MEGA software (RRID:SCR_000667) (https://www.megasoftware.net/), using the neighbor join method (tests performed with 1000 bootstrap replicas), with further graphical optimization accomplished in the iTol webtool (RRID:SCR_018174) (https://itol.embl.de/).

### Supplementary Information

**Additional file 1: Table S1.** Amount of accession numbers collected according to the different approaches. **Table S2.** List of the 313 ZmHSPs candidates previously identified after redundancy elimination. **Table S3.** List of the 182 validated ZmHSPs, organized according to: locus' chromosomal position; number of transcripts; canonical transcript size (bp); family, proposed class (subfamily), number of amino acids, MW (in kDa) and isoelectric point (pI) of the encoded protein. **Table S4.** List of the 182 validated ZmHSPs, organized according to their multiple nomenclatures, regarded the different notations found at MaizeGDB, NCBI and Plaza, besides some arbitrary names used on papers and such. **Table S5.** List of the 182 validated ZmHSPs, organized according to their multiple nomenclatures, regarded the different notations found at UniProtKB, GenBank and other databases. **Table S6.** List of the orthologous genes to those coding for the 182 maize HSPs, identified in the sorghum and rice genomes. **Table S7.** List of the orthologous genes to those coding for the 182 maize HSPs, identified in the millet, brachypodium and arabidopsis genomes. **Table S8.** List of motifs identified in each ZmHSP family. **Table S9.** Codes of the protein families related to the conserved domains identified in each ZmHSP family, according to data from the PFAM and PROSITE platforms. **Table S10.** List of ZmHSPs that had their expression profiles significantly altered after exposure to certain types of abiotic stresses. **Table S11.** Percent identity matrix of three ZmHSPs groups, where the occurrence of gene duplication was conjectured. **Table S12.** List of target and normalizers genes, as well as their respective primers, used for the expression analyses on seeds from four tropical maize inbred lines, previously submitted to two drying temperatures (35 and 50 °C). **Fig. S1.** Alignment of the fourteen validated ZmHSP100s/CLPs. **Fig. S2.** Alignment of the eleven validated ZmHSP90s. **Fig. S3.** Alignment of the twenty-nine validated ZmHSP70s. **Fig. S4.** Alignment of the twenty-three validated ZmCPN60s. **Fig. S5.** Alignment of the ten validated ZmCPN10s. **Fig. S6.** Alignment of the fifty-three validated ZmHSP40s/DNAJs. **Fig. S7.** Alignment of the forty-two validated ZmSmallHSPs. **Fig. S8. **Changes in the expression levels of seven ZmHSPs, measured after the seeds of four tropical maize inbred lines have been subjected to two different drying temperatures. **Fig. S9.** Phylogenetic tree of the 182 validated ZmHSPs.

**Additional file 2: Sup. Spr. 1. **Subcellular localization.** Sup. Spr. 2.** Cellular components. **Sup. Spr. 3. **Biological processes. **Sup. Spr. 4. **Molecular functions. **Sup. Spr. 5.** Expression patterns.

**Additional file 3.** Results of the relative expression analyses of seven ZmHSPs genes after seeds from four maize inbred lines had been submitted to two different drying temperature: 35 °C (normal temperature) and 50 °C (high temperature, a significant abiotic stress).

## Data Availability

All data generated or analyzed during this study are included in this published article and/or on its three supplementary information files, as the following documents.

## Abbreviations

AA: Amino acid
ABA: Abscisic acid
AbSts: Abiotic stresses
Add'l File: Additional File
APPJ: Cold plasma jets at atmospheric pressure
BLAST: Basic local alignment search tool
cAMP: Cyclic adenosine monophosphate
CCT: Chaperonin containing TCP-1
CD: Conserved domains
CPNs: Chaperonins
CS: Chloroplast stroma
DSBs: Double-stranded DNA breaks
EMS: Endomembrane system
ER: Endoplasmic reticulum
FC: Fold change
GO: Gene ontology
HMMs: Profile hidden Markov models
HSFs: Heat shock proteins’ transcription factors
HSPIR: Heat Shock Protein Information Resource
HSPs: Heat shock proteins
HSR: Thermal shock response
IRES: Internal ribosome entry site
kDa: Kilodalton
MW: Molecular weight
OLGs: Overlapping genes
QTL: Quantitative trait locus
RNP: Ribonucleoprotein
ROS: Reactive oxygen species
RuBisCO: Ribulose 1,5-bisphosphate carboxylase-oxygenase
SNCs: Conserved non-coding regions
ssDNA: Single-stranded DNA
Figure S: Supplementary figure
Sup. Spr.: Supplementary spreadsheet
Table S: Supplementary table
TFs: Transcription factors
TRiC: T-complex protein Ring Complex
UPR: Unfolded protein response
UTR: Untranslated region
ZmCPNs: Maize chaperonins
ZmHSPs: Maize heat shock proteins
