# Supplementary material for: Maize heat shock proteins—prospection, validation, categorization and in silico analysis of the different ZmHSP families
Source: Stress Biol. 2023 Sep 6;3(1):37. doi: 10.1007/s44154-023-00104-2 (PMC10482818; doi:10.1007/s44154-023-00104-2)
Supplement: Supplementary file 1 — Additional file 1: Table S1. Amount of accession numbers collected according to the different approaches. Table S2. List of the 313 ZmHSPs candidates previously identified after redundancy elimination. Table S3. List of the 182 validated ZmHSPs, organized according to: locus' chromosomal position; number of transcripts; canonical transcript size (bp); family, proposed class (subfamily), number of amino acids, MW (in kDa) and isoelectric point (pI) of the encoded protein. Table S4. List of the 182 validated ZmHSPs, organized according to their multiple nomenclatures, regarded the different notations found at MaizeGDB, NCBI and Plaza, besides some arbitrary names used on papers and such. Table S5. List of the 182 validated ZmHSPs, organized according to their multiple nomenclatures, regarded the different notations found at UniProtKB, GenBank and other databases. Table S6. List of the orthologous genes to those coding for the 182 maize HSPs, identified in the sorghum and rice genomes. Table S7. List of the orthologous genes to those coding for the 182 maize HSPs, identified in the millet, brachypodium and arabidopsis genomes. Table S8. List of motifs identified in each ZmHSP family. Table S9. Codes of the protein families related to the conserved domains identified in each ZmHSP family, according to data from the PFAM and PROSITE platforms. Table S10. List of ZmHSPs that had their expression profiles significantly altered after exposure to certain types of abiotic stresses. Table S11. Percent identity matrix of three ZmHSPs groups, where the occurrence of gene duplication was conjectured. Table S12. List of target and normalizers genes, as well as their respective primers, used for the expression analyses on seeds from four tropical maize inbred lines, previously submitted to two drying temperatures (35 and 50 °C). Fig. S1. Alignment of the fourteen validated ZmHSP100s/CLPs. Fig. S2. Alignment of the eleven validated ZmHSP90s. Fig. S3. Alignment of the twenty-nine val [file 44154_2023_104_MOESM1_ESM.docx]

**ADDITIONAL FILE 01**

*** Manuscript being resubmitted, after requested revisions,**

**as "Original Paper" to the Stress Biology journal**

**Maize heat shock proteins - Prospection, validation, categorization and *in silico* analysis of the different ZmHSP families**

Rubens Diogo-Jr.^1,^^2✉^, Edila Vilela de Resende von Pinho^2^, Renan Terassi Pinto^3^, Lingrui Zhang^1^, Jorge Alberto Condori-Apfata^1,4^, Paula Andrade Pereira^2^, Danielle Rezende Vilela^2^

^1^ Department of Horticulture and Landscape Architecture, Purdue University, West Lafayette-IN (47907), USA;

^2^ Department of Agriculture, Federal University of Lavras (UFLA), Lavras-MG (37200-900), Brazil;

^3^ Faculty of Philosophy and Sciences at Ribeirao Preto, University of Sao Paulo (USP), Ribeirao Preto-SP (14040-901), Brazil;

^4^ Faculty of Engineering and Agricultural Sciences, Universidad Nacional Toribio Rodriguez de Mendoza de Amazonas (UNTRM), Chachapoyas-AM (01001), Peru.

^✉^ Correspondence: rubens.junior@estudante.ufla.br

**This PDF includes:**

**Supplementary Tables: Tables S1 to S12**

**Supplementary Figures: Figures S1 to S9**

**June 2023**

**Table S1** Amount of accession numbers collected according to the different approaches:

|  | **Source** | | | **After Clustering** | **Approved** | **Excluded** |
| --- | --- | --- | --- | --- | --- | --- |
| **Family** | **HSPIR** | **MaizeGDB** | **HMMSearch** |  |  |  |
| sHSPs/HSP20s | 69 | 25 | 46 (PF00011) | 57 | 42 | 15 |
| HSP40s/DNAJs | 91 | 45 | 215 (PF00226) | 99 | 53 | 46 |
|  |  |  | 69 (PF01556) |  |  |  |
|  |  |  | 14 (PF14308) |  |  |  |
|  |  |  | 46 (PF00684) |  |  |  |
| HSP/CPN60s | 41 | 25 | 74 (PF00118) | 53 | 23 | 30 |
| HSP/CPN10s |  |  | 13 (PF00166) | 13 | 10 | 3 |
| HSP70s | 37 | 22 | 83 (PF00012) | 51 | 29 | 22 |
| HSP90s | 14 | 7 | 24 (PF00183) | 13 | 11 | 2 |
| HSP100s/CLPs | 12 | 7 | 34 (PF02861) | 27 | 14 | 13 |
|  |  |  | 28 (PF10431) |  |  |  |
| **Subtotal** | **264** | **131** | **646** | **313** | **182** | **131** |

**Table S2** List of the 313 ZmHSPs candidates previously identified after redundancy elimination. This list includes the 182 validated (divided according their respective families, also subdivided into classes/subfamilies) and the 131 excluded ones:

**ZmHSP100s class A** (nine proteins): Zm00001eb020330, Zm00001eb084420, Zm00001eb084890, Zm00001eb234160, Zm00001eb242420, Zm00001eb293780, Zm00001eb393250, Zm00001eb411490 and Zm00001eb421360.

**ZmHSP100s class B** (five proteins): Zm00001eb086360, Zm00001eb178400, Zm00001eb197530, Zm00001eb412800 and Zm00001eb420410.

**Putative ZmHSP100s excluded from final list** (thirteen proteins): two CLPAs (Zm00001eb025630 and Zm00001eb157760) that share only motif 2 with the other putative HSP100s; five CLPXs (Zm00001eb182540, Zm00001eb236990, Zm00001eb244240, Zm00001eb293170 and Zm00001eb365990) which sequences have some homology' degree to those CLPBs; three SMAX1-like (Zm00001eb164080, Zm00001eb243020 and Zm00001eb422090), that only show motif 5; and also three uncharacterized proteins (Zm00001eb021370, Zm00001eb234580 and Zm00001eb334170) that only exhibit a single motif (1, 2 and 4, respectively), a criterion considered insufficient for such cataloging.

**ZmHSP90s class A** (ten proteins): Zm00001eb101750, Zm00001eb137900, Zm00001eb199230, Zm00001eb199590, Zm00001eb228120, Zm00001eb264470, Zm00001eb272290, Zm00001eb315880, Zm00001eb316410 and Zm00001eb418610.

**ZmHSP90s class B** (one protein): Zm00001eb034130.

**Putative ZmHSP90s excluded from final list** (two proteins): two (Zm00001eb061370 and Zm00001eb402920) present only motif 3, which (alone) didn’t look sufficiently characteristic to us.

**ZmHSP70s class A** (twenty proteins): Zm00001eb012470, Zm00001eb012510, Zm00001eb029200, Zm00001eb043470, Zm00001eb053750, Zm00001eb101990, Zm00001eb133480, Zm00001eb136490, Zm00001eb148420, Zm00001eb165400, Zm00001eb209550, Zm00001eb214940, Zm00001eb217010, Zm00001eb220140, Zm00001eb229930, Zm00001eb351340, Zm00001eb368000, Zm00001eb397600, Zm00001eb400020 and Zm00001eb409950.

**ZmHSP70s class B** (three proteins): Zm00001eb115870, Zm00001eb188700 and Zm00001eb252410.

**ZmHSP70s class C** (six proteins): Zm00001eb224550, Zm00001eb273690, Zm00001eb283640, Zm00001eb283800, Zm00001eb346940 and Zm00001eb346960.

**Putative ZmHSP70s excluded from final list** (twenty-two proteins): all them (Zm00001eb091040, Zm00001eb188750, Zm00001eb231480, Zm00001eb280370, Zm00001eb301370, Zm00001eb301610, Zm00001eb309670, Zm00001eb310680, Zm00001eb316900, Zm00001eb358790, Zm00001eb383180, Zm00001eb395140, Zm00001eb403610, Zm00001eb419860, Zm00001eb423680, Zm00001eb423690, Zm00001eb423720, Zm00001eb423730, Zm00001eb423740, Zm00001eb423790, Zm00001eb423800 and Zm00001eb432270) having just a single region (generally motifs 5 and/or 6) common to the other HSP70s, but nothing more, which culminates in a very low molecular weight (between 10 and 30 kDa).

**ZmCPN60s class A** (nine proteins): Zm00001eb010540, Zm00001eb046580, Zm00001eb065160, Zm00001eb209960, Zm00001eb222300, Zm00001eb268910, Zm00001eb286730, Zm00001eb294310 and Zm00001eb378930.

**ZmCPN60s class B** (fourteen proteins): Zm00001eb001240, Zm00001eb008400, Zm00001eb111720, Zm00001eb114320, Zm00001eb118030, Zm00001eb127430, Zm00001eb189870, Zm00001eb192630, Zm00001eb200400, Zm00001eb204950, Zm00001eb207780, Zm00001eb257580, Zm00001eb298950 and Zm00001eb333890.

**Putative ZmCPN60s excluded from final list** (thirty proteins): all exhibiting any conserved domain typical of CPN60s, some of them (twenty) smaller (10-30 kDa) (Zm00001eb025750, Zm00001eb034680, Zm00001eb035510, Zm00001eb045220, Zm00001eb087180, Zm00001eb096310, Zm00001eb096320, Zm00001eb096330, Zm00001eb121080, Zm00001eb121090, Zm00001eb121100, Zm00001eb130780, Zm00001eb131780, Zm00001eb178260, Zm00001eb210250, Zm00001eb212460, Zm00001eb250320, Zm00001eb290420, Zm00001eb382370 and Zm00001eb424810) and other ten much bigger (100 to 200 kDa) (Zm00001eb019420, Zm00001eb041840, Zm00001eb076550, Zm00001eb100110, Zm00001eb146180, Zm00001eb174440, Zm00001eb177150, Zm00001eb260170, Zm00001eb280640 and Zm00001eb312210).

**ZmCPN10s class A** (seven proteins): Zm00001eb017830, Zm00001eb024030, Zm00001eb071630, Zm00001eb110270, Zm00001eb290400, Zm00001eb329030 and Zm00001eb390720.

**ZmCPN10s class B** (three proteins): Zm00001eb100100, Zm00001eb193040 and Zm00001eb373960.

**Putative ZmCPN10s excluded from final list** (three proteins): all (Zm00001eb266000, Zm00001eb329020 and Zm00001eb365450) haven’t showed the family related motifs.

**ZmHSP40s class A** (nineteen proteins): Zm00001eb050280, Zm00001eb060050, Zm00001eb091890, Zm00001eb103670, Zm00001eb107580, Zm00001eb172900, Zm00001eb192080, Zm00001eb213310, Zm00001eb218560, Zm00001eb249430, Zm00001eb258200, Zm00001eb269190, Zm00001eb294320, Zm00001eb379790, Zm00001eb392350, Zm00001eb404330, Zm00001eb407790, Zm00001eb416450 and Zm00001eb429820.

**ZmHSP40s class B** (fifteen proteins): Zm00001eb009060, Zm00001eb042680, Zm00001eb118880, Zm00001eb208880, Zm00001eb240640, Zm00001eb282660, Zm00001eb288550, Zm00001eb295770, Zm00001eb339480, Zm00001eb340880, Zm00001eb343630, Zm00001eb345630, Zm00001eb354940, Zm00001eb366410 and Zm00001eb415590.

**ZmHSP40s class C** (six proteins): Zm00001eb035420, Zm00001eb243690, Zm00001eb294570, Zm00001eb317080, Zm00001eb342590 and Zm00001eb361030.

**ZmHSP40s class D** (four proteins): Zm00001eb409280, Zm00001eb409300, Zm00001eb409350 and Zm00001eb409390.

**ZmHSP40s class E** (nine proteins): Zm00001eb137150, Zm00001eb146070, Zm00001eb162560, Zm00001eb205970, Zm00001eb236340, Zm00001eb254160, Zm00001eb279930, Zm00001eb342290 and Zm00001eb346620.

**Putative ZmHSP40s excluded from final list** (forty-six proteins): twelve (Zm00001eb009070, Zm00001eb021850, Zm00001eb050270, Zm00001eb052720, Zm00001eb086520, Zm00001eb157250, Zm00001eb209410, Zm00001eb218540, Zm00001eb220290, Zm00001eb228100, Zm00001eb362030 and Zm00001eb391460), although previously cataloged as alleged HSP40s, haven’t align properly; other candidates, in turn, proved to be too incomplete: fourteen from 10 to 25 kDa (Zm00001eb010480, Zm00001eb016700, Zm00001eb028880, Zm00001eb062810, Zm00001eb083700, Zm00001eb103660, Zm00001eb113670, Zm00001eb127110, Zm00001eb185180, Zm00001eb245890, Zm00001eb278850, Zm00001eb311170, Zm00001eb315320 and Zm00001eb334090) and thirteen others over 75 kDa (Zm00001eb054940, Zm00001eb085350, Zm00001eb085870, Zm00001eb085940, Zm00001eb180900, Zm00001eb200390, Zm00001eb334270, Zm00001eb334280, Zm00001eb364230, Zm00001eb421150, Zm00001eb423990, Zm00001eb423990 and Zm00001eb424000), besides the molecular weight issue, contain only a small portion of the sequence inherent to the J-domain N-terminal region (and nothing else conserved downstream); five (Zm00001eb017510, Zm00001eb018130, Zm00001eb106600, Zm00001eb293140 and Zm00001eb318980) were excluded because they present only a part of the C-terminal region; then other two (Zm00001eb391470 and Zm00001eb391480) possess only the typical domain of J-like proteins.

**ZmSmallHSPs class A** (seven proteins): Zm00001eb011880, Zm00001eb011900, Zm00001eb011930, Zm00001eb124940, Zm00001eb124960, Zm00001eb335430 and Zm00001eb397940.

**ZmSmallHSPs class B** (seven proteins): Zm00001eb121900, Zm00001eb257130, Zm00001eb311990, Zm00001eb337690, Zm00001eb395330, Zm00001eb395360 and Zm00001eb395370.

**ZmSmallHSPs class C** (ten proteins): Zm00001eb081770, Zm00001eb112330, Zm00001eb125010, Zm00001eb125030, Zm00001eb208810, Zm00001eb252460, Zm00001eb291920, Zm00001eb352720, Zm00001eb371530 and Zm00001eb423300.

**ZmSmallHSPs class D** (eighteen proteins): Zm00001eb010530, Zm00001eb026540, Zm00001eb026680, Zm00001eb026690, Zm00001eb032310, Zm00001eb034050, Zm00001eb047490, Zm00001eb052730, Zm00001eb124950, Zm00001eb193940, Zm00001eb218280, Zm00001eb218310, Zm00001eb222770, Zm00001eb222820, Zm00001eb235800, Zm00001eb297350, Zm00001eb322740 and Zm00001eb372830.

**Putative ZmSmallHSPs excluded from final list** (fifteen proteins): five supposedly identified sHSPs superimposed on complementary strand (Zm00001eb011890, Zm00001eb125000, Zm00001eb297340, Zm00001eb337680) to validated sHSPs, sometimes even palindromicly (Zm00001eb222810); four ARIDs transcriptional factors (Zm00001eb188950, Zm00001eb252640, Zm00001eb275690 and Zm00001eb388060) of higher molecular weight that show a certain analogy to the sHSPs aminoacidic chains; and six proteins (Zm00001eb011890, Zm00001eb222810, Zm00001eb233440, Zm00001eb298480, Zm00001eb337680 and Zm00001eb357380) which, despite the term “HSP” in the name, have no any motif characteristic of the aforementioned classes.

**Table S3** List of the 182 validated ZmHSPs, already with the nomenclature that we are suggesting, organized according to: *locus* chromosomal position; number of transcripts; canonical transcript size (bp); family, proposed class (subfamily), number of amino acids, molecular weight (in kDa) and isoelectric point (pI) of the encoded protein:

| **#** | **Zm-B73-NAM-5.0** | **Suggested Name** | **Chromosome - Locus** | **Strand** | **Class** | **AAs** | **MW (kDa)** | **pI** | **Length (bp)** | **Transcript Number** |
| --- | --- | --- | --- | --- | --- | --- | --- | --- | --- | --- |
| *1* | Zm00001eb011880 | **sHSP-A01(Chr1+)** | Chr1:038574688-038575736 | + | A | 158 | 17,79 | 5,43 | 1048 | 1 |
| *2* | Zm00001eb011900 | **sHSP-A02(Chr1+)** | Chr1:038662195-038663648 | + | A | 158 | 17,76 | 5,43 | 1453 | 2 |
| *3* | Zm00001eb011930 | **sHSP-A03(Chr1+)** | Chr1:038791422-038792306 | + | A | 159 | 17,88 | 7,08 | 884 | 1 |
| *4* | Zm00001eb124940 | **sHSP-A04(Chr3-)** | Chr3:020467510-020468557 | - | A | 154 | 17,06 | 7,02 | 1047 | 1 |
| *5* | Zm00001eb124960 | **sHSP-A05(Chr3+)** | Chr3:020587711-020588602 | + | A | 152 | 17,23 | 5,66 | 891 | 1 |
| *6* | Zm00001eb335430 | **sHSP-A06(Chr8-)** | Chr8:013446129-013447047 | - | A | 155 | 17,34 | 5,67 | 918 | 1 |
| *7* | Zm00001eb397940 | **sHSP-A07(Chr9+)** | Chr9:146472960-146473813 | + | A | 163 | 18,02 | 5,42 | 853 | 1 |
| *8* | Zm00001eb121900 | **sHSP-B01(Chr3+)** | Chr3:008819782-008820963 | + | B | 165 | 17,87 | 5,82 | 1181 | 1 |
| *9* | Zm00001eb257130 | **sHSP-B02(Chr5+)** | Chr5:220633019-220634115 | + | B | 171 | 18,35 | 6,36 | 1096 | 1 |
| *10* | Zm00001eb311990 | **sHSP-B03(Chr7+)** | Chr7:107630591-107631239 | + | B | 160 | 17,46 | 5,89 | 576 | 1 |
| *11* | Zm00001eb337690 | **sHSP-B04(Chr8-)** | Chr8:022215123-022216180 | - | B | 164 | 17,80 | 5,23 | 1057 | 1 |
| *12* | Zm00001eb395330 | **sHSP-B05(Chr9+)** | Chr9:138243093-138244008 | + | B | 160 | 17,45 | 5,99 | 915 | 1 |
| *13* | Zm00001eb395360 | **sHSP-B06(Chr9+)** | Chr9:138502278-138503208 | + | B | 154 | 17,05 | 7,66 | 930 | 1 |
| *14* | Zm00001eb395370 | **sHSP-B07(Chr9+)** | Chr9:138662842-138663742 | + | B | 154 | 17,05 | 7,66 | 813 | 1 |
| *15* | Zm00001eb081770 | **sHSP-C01(Chr2-)** | Chr2:047778184-047779308 | - | C | 208 | 22,83 | 5,78 | 1124 | 1 |
| *16* | Zm00001eb112330 | **sHSP-C02(Chr2-)** | Chr2:226318678-226319544 | - | C | 213 | 22,74 | 6,14 | 866 | 1 |
| *17* | Zm00001eb125010 | **sHSP-C03(Chr3-)** | Chr3:020636265-020637167 | - | C | 273 | 29,23 | 9,42 | 902 | 1 |
| *18* | Zm00001eb125030 | **sHSP-C04(Chr3-)** | Chr3:020688973-020690215 | - | C | 149 | 16,65 | 6,03 | 1242 | 1 |
| *19* | Zm00001eb208810 | **sHSP-C05(Chr4+)** | Chr4:247704206-247704996 | + | C | 184 | 19,86 | 7,19 | 790 | 1 |
| *20* | Zm00001eb252460 | **sHSP-C06(Chr5+)** | Chr5:210133404-210134536 | + | C | 157 | 16,69 | 7,25 | 1132 | 1 |
| *21* | Zm00001eb291920 | **sHSP-C07(Chr6+)** | Chr6:168208375-168210639 | + | C | 197 | 21,88 | 4,63 | 2264 | 1 |
| *22* | Zm00001eb352720 | **sHSP-C08(Chr8+)** | Chr8:124547599-124549579 | + | C | 190 | 21,19 | 4,71 | 1980 | 1 |
| *23* | Zm00001eb371530 | **sHSP-C09(Chr9-)** | Chr9:002328307-002329279 | - | C | 146 | 15,85 | 8,06 | 972 | 1 |
| *24* | Zm00001eb423300 | **sHSP-C10(Chr10+)** | Chr10:122371558-122372715 | + | C | 217 | 23,44 | 5,81 | 1157 | 1 |
| *25* | Zm00001eb010530 | **sHSP-D01(Chr1+)** | Chr1:033450523-033451655 | + | D | 240 | 26,38 | 7,66 | 1132 | 1 |
| *26* | Zm00001eb026540 | **sHSP-D02(Chr1-)** | Chr1:122090601-122091601 | - | D | 185 | 20,19 | 8,06 | 1000 | 1 |
| *27* | Zm00001eb026680 | **sHSP-D03(Chr1-)** | Chr1:123912382-123913569 | - | D | 221 | 23,93 | 8,48 | 1187 | 2 |
| *28* | Zm00001eb026690 | **sHSP-D04(Chr1-)** | Chr1:124029455-124030640 | - | D | 208 | 22,62 | 8,58 | 1185 | 2 |
| *29* | Zm00001eb032310 | **sHSP-D05(Chr1-)** | Chr1:180913725-180914598 | - | D | 200 | 22,25 | 10,10 | 873 | 1 |
| *30* | Zm00001eb034050 | **sHSP-D06(Chr1-)** | Chr1:188096008-188100376 | - | D | 304 | 33,12 | 9,24 | 1368 | 1 |
| *31* | Zm00001eb047490 | **sHSP-D07(Chr1-)** | Chr1:243850571-243851742 | - | D | 200 | 21,80 | 9,20 | 1171 | 1 |
| *32* | Zm00001eb052730 | **sHSP-D08(Chr1+)** | Chr1:265713613-265714958 | + | D | 290 | 31,75 | 6,57 | 1345 | 1 |
| *33* | Zm00001eb124950 | **sHSP-D09(Chr3+)** | Chr3:020490417-020491182 | + | D | 188 | 21,26 | 8,80 | 765 | 1 |
| *34* | Zm00001eb193940 | **sHSP-D10(Chr4-)** | Chr4:184768056-184769410 | - | D | 219 | 23,87 | 7,13 | 1354 | 3 |
| *35* | Zm00001eb218280 | **sHSP-D11(Chr5+)** | Chr5:015533079-015534721 | + | D | 342 | 36,84 | 8,52 | 1507 | 2 |
| *36* | Zm00001eb218310 | **sHSP-D12(Chr5+)** | Chr5:016267532-016268426 | + | D | 260 | 28,42 | 8,76 | 894 | 1 |
| *37* | Zm00001eb222770 | **sHSP-D13(Chr5-)** | Chr5:034381356-034382293 | - | D | 200 | 21,61 | 7,80 | 937 | 1 |
| *38* | Zm00001eb222820 | **sHSP-D14(Chr5-)** | Chr5:034490337-034491630 | - | D | 253 | 27,08 | 8,15 | 1275 | 2 |
| *39* | Zm00001eb235800 | **sHSP-D15(Chr5+)** | Chr5:117986616-117987732 | + | D | 211 | 22,82 | 6,89 | 1116 | 1 |
| *40* | Zm00001eb297350 | **sHSP-D16(Chr6-)** | Chr6:178981640-178982873 | - | D | 304 | 32,06 | 8,22 | 1233 | 1 |
| *41* | Zm00001eb322740 | **sHSP-D17(Chr7+)** | Chr7:162303093-162303883 | + | D | 155 | 17,16 | 6,40 | 790 | 1 |
| *42* | Zm00001eb372830 | **sHSP-D18(Chr9+)** | Chr9:007799475-007800671 | + | D | 208 | 22,28 | 5,30 | 1196 | 1 |
| *43* | Zm00001eb050280 | **HSP40-A01(Chr1-)** | Chr1:256248442-256252143 | - | A | 419 | 46,73 | 5,81 | 3659 | 4 |
| *44* | Zm00001eb060050 | **HSP40-A02(Chr1+)** | Chr1:292715146-292718283 | + | A | 418 | 46,65 | 5,62 | 3137 | 1 |
| *45* | Zm00001eb091890 | **HSP40-A03(Chr2-)** | Chr2:142427575-142431164 | - | A | 321 | 35,96 | 5,21 | 3589 | 1 |
| *46* | Zm00001eb103670 | **HSP40-A04(Chr2+)** | Chr2:202575590-202576488 | + | A | 235 | 26,84 | 4,90 | 898 | 1 |
| *47* | Zm00001eb107580 | **HSP40-A05(Chr2+)** | Chr2:213881529-213887745 | + | A | 394 | 44,28 | 5,03 | 6216 | 1 |
| *48* | Zm00001eb172900 | **HSP40-A06(Chr4-)** | Chr4:036495353-036499977 | - | A | 448 | 48,02 | 8,62 | 4624 | 3 |
| *49* | Zm00001eb192080 | **HSP40-A07(Chr4+)** | Chr4:178286234-178298582 | + | A | 493 | 52,76 | 8,51 | 12129 | 2 |
| *50* | Zm00001eb213310 | **HSP40-A08(Chr5-)** | Chr5:004976950-004980219 | - | A | 417 | 46,57 | 5,53 | 3269 | 1 |
| *51* | Zm00001eb218560 | **HSP40-A09(Chr5-)** | Chr5:017049360-017054387 | - | A | 419 | 46,62 | 5,90 | 5027 | 4 |
| *52* | Zm00001eb249430 | **HSP40-A10(Chr5+)** | Chr5:196100776-196107759 | + | A | 422 | 47,09 | 6,30 | 6605 | 2 |
| *53* | Zm00001eb258200 | **HSP40-A11(Chr5-)** | Chr5:222788619-222799235 | - | A | 488 | 52,17 | 8,19 | 10616 | 4 |
| *54* | Zm00001eb269190 | **HSP40-A12(Chr6-)** | Chr6:070454718-070459962 | - | A | 429 | 46,33 | 7,89 | 5026 | 4 |
| *55* | Zm00001eb294320 | **HSP40-A13(Chr6-)** | Chr6:173256396-173261446 | - | A | 379 | 41,11 | 5,75 | 5050 | 1 |
| *56* | Zm00001eb379790 | **HSP40-A14(Chr9+)** | Chr9:032754838-032757839 | + | A | 415 | 46,38 | 5,38 | 3001 | 1 |
| *57* | Zm00001eb392350 | **HSP40-A15(Chr9-)** | Chr9:125316279-125321329 | - | A | 328 | 34,87 | 7,09 | 5050 | 1 |
| *58* | Zm00001eb404330 | **HSP40-A16(Chr9-)** | Chr9:161322652-161325030 | - | A | 312 | 35,23 | 5,40 | 2378 | 1 |
| *59* | Zm00001eb407790 | **HSP40-A17(Chr10+)** | Chr10:009189670-009206858 | + | A | 418 | 45,46 | 7,87 | 16868 | 5 |
| *60* | Zm00001eb416450 | **HSP40-A18(Chr10-)** | Chr10:083210894-083215232 | - | A | 448 | 48,14 | 8,72 | 4318 | 3 |
| *61* | Zm00001eb429820 | **HSP40-A19(Chr10+)** | Chr10:141994415-141997453 | + | A | 433 | 48,79 | 5,07 | 3038 | 1 |
| *62* | Zm00001eb009060 | **HSP40-B01(Chr1+)** | Chr1:027838646-027855600 | + | B | 538 | 59,83 | 8,32 | 16954 | 1 |
| *63* | Zm00001eb042680 | **HSP40-B02(Chr1+)** | Chr1:225018041-225019798 | + | B | 341 | 37,31 | 5,33 | 1757 | 1 |
| *64* | Zm00001eb118880 | **HSP40-B03(Chr3-)** | Chr3:001537879-001542296 | - | B | 336 | 37,02 | 8,62 | 4417 | 1 |
| *65* | Zm00001eb208880 | **HSP40-B04(Chr4-)** | Chr4:247792418-247795733 | - | B | 338 | 37,99 | 8,38 | 3311 | 2 |
| *66* | Zm00001eb240640 | **HSP40-B05(Chr5-)** | Chr5:161796886-161810286 | - | B | 346 | 38,20 | 7,99 | 13400 | 2 |
| *67* | Zm00001eb282660 | **HSP40-B06(Chr6-)** | Chr6:137444553-137452787 | - | B | 350 | 39,31 | 5,79 | 7824 | 3 |
| *68* | Zm00001eb288550 | **HSP40-B07(Chr6-)** | Chr6:159408954-159411279 | - | B | 335 | 36,38 | 8,97 | 2271 | 2 |
| *69* | Zm00001eb295770 | **HSP40-B08(Chr6-)** | Chr6:175833424-175835739 | - | B | 362 | 38,66 | 8,20 | 2315 | 1 |
| *70* | Zm00001eb339480 | **HSP40-B09(Chr8+)** | Chr8:030517236-030520362 | + | B | 336 | 36,82 | 8,50 | 3126 | 2 |
| *71* | Zm00001eb340880 | **HSP40-B10(Chr8+)** | Chr8:043131153-043134549 | + | B | 377 | 42,72 | 6,24 | 3396 | 1 |
| *72* | Zm00001eb343630 | **HSP40-B11(Chr8-)** | Chr8:070207576-070209859 | - | B | 353 | 38,02 | 8,31 | 2272 | 2 |
| *73* | Zm00001eb345630 | **HSP40-B12(Chr8-)** | Chr8:080981090-080990017 | - | B | 350 | 39,22 | 6,73 | 8927 | 3 |
| *74* | Zm00001eb354940 | **HSP40-B13(Chr8-)** | Chr8:134709953-134713774 | - | B | 326 | 35,27 | 8,57 | 3821 | 1 |
| *75* | Zm00001eb366410 | **HSP40-B14(Chr8-)** | Chr8:172072514-172075285 | - | B | 362 | 39,50 | 8,64 | 2771 | 2 |
| *76* | Zm00001eb415590 | **HSP40-B15(Chr10+)** | Chr10:078340672-078343058 | + | B | 348 | 38,42 | 8,56 | 2386 | 1 |
| *77* | Zm00001eb035420 | **HSP40-C01(Chr1-)** | Chr1:193831465-193837096 | - | C | 390 | 43,64 | 5,23 | 5427 | 2 |
| *78* | Zm00001eb243690 | **HSP40-C02(Chr5+)** | Chr5:176230665-176234022 | + | C | 386 | 43,32 | 5,27 | 3357 | 2 |
| *79* | Zm00001eb294570 | **HSP40-C03(Chr6-)** | Chr6:173618088-173621891 | - | C | 338 | 37,81 | 5,47 | 3803 | 6 |
| *80* | Zm00001eb317080 | **HSP40-C04(Chr7-)** | Chr7:139515966-139522756 | - | C | 394 | 44,45 | 4,85 | 6790 | 2 |
| *81* | Zm00001eb342590 | **HSP40-C05(Chr8-)** | Chr8:064631958-064635399 | - | C | 338 | 37,96 | 6,39 | 3441 | 4 |
| *82* | Zm00001eb361030 | **HSP40-C06(Chr8+)** | Chr8:157402317-157407964 | + | C | 336 | 37,89 | 5,22 | 5647 | 1 |
| *83* | Zm00001eb409280 | **HSP40-D01(Chr10-)** | Chr10:016630294-016634834 | - | D | 604 | 67,96 | 5,57 | 4397 | 2 |
| *84* | Zm00001eb409300 | **HSP40-D02(Chr10-)** | Chr10:016896223-016904624 | - | D | 466 | 52,77 | 5,83 | 1570 | 2 |
| *85* | Zm00001eb409350 | **HSP40-D03(Chr10-)** | Chr10:017035793-017037482 | - | D | 466 | 52,59 | 5,72 | 1689 | 1 |
| *86* | Zm00001eb409390 | **HSP40-D04(Chr10-)** | Chr10:017116227-017117954 | - | D | 466 | 52,66 | 5,83 | 1727 | 1 |
| *87* | Zm00001eb137150 | **HSP40-E01(Chr3-)** | Chr3:131008931-131020175 | - | E | 407 | 45,55 | 5,48 | 11244 | 1 |
| *88* | Zm00001eb146070 | **HSP40-E02(Chr3-)** | Chr3:177693040-177695787 | - | E | 382 | 41,10 | 10,32 | 2747 | 3 |
| *89* | Zm00001eb162560 | **HSP40-E03(Chr3+)** | Chr3:232825991-232833751 | + | E | 375 | 42,41 | 7,31 | 7760 | 3 |
| *90* | Zm00001eb205970 | **HSP40-E04(Chr4-)** | Chr4:241832549-241836640 | - | E | 286 | 32,83 | 7,43 | 3701 | 5 |
| *91* | Zm00001eb236340 | **HSP40-E05(Chr5-)** | Chr5:124469886-124474031 | - | E | 284 | 32,35 | 7,11 | 4145 | 1 |
| *92* | Zm00001eb254160 | **HSP40-E06(Chr5+)** | Chr5:214231585-214236538 | + | E | 441 | 49,20 | 7,79 | 4953 | 3 |
| *93* | Zm00001eb279930 | **HSP40-E07(Chr6-)** | Chr6:125265594-125271612 | - | E | 441 | 49,32 | 7,89 | 6018 | 1 |
| *94* | Zm00001eb342290 | **HSP40-E08(Chr8-)** | Chr8:063098281-063106566 | - | E | 370 | 41,95 | 7,32 | 8285 | 3 |
| *95* | Zm00001eb346620 | **HSP40-E09(Chr8+)** | Chr8:089302111-089305932 | + | E | 373 | 43,30 | 8,67 | 3404 | 5 |
| *96* | Zm00001eb010540 | **CPN60-A01(Chr1-)** | Chr1:033461325-033465763 | - | A | 588 | 63,53 | 5,40 | 4336 | 2 |
| *97* | Zm00001eb046580 | **CPN60-A02(Chr1-)** | Chr1:240288964-240294856 | - | A | 576 | 60,94 | 5,54 | 5892 | 1 |
| *98* | Zm00001eb065160 | **CPN60-A03(Chr1-)** | Chr1:307034584-307038939 | - | A | 584 | 61,41 | 5,12 | 4355 | 1 |
| *99* | Zm00001eb209960 | **CPN60-A04(Chr4-)** | Chr4:249553936-249558464 | - | A | 605 | 64,37 | 5,67 | 4528 | 1 |
| *100* | Zm00001eb222300 | **CPN60-A05(Chr5-)** | Chr5:032327957-032333903 | - | A | 577 | 61,22 | 5,55 | 5946 | 2 |
| *101* | Zm00001eb268910 | **CPN60-A06(Chr6-)** | Chr6:067852001-067858417 | - | A | 579 | 61,86 | 5,34 | 6416 | 2 |
| *102* | Zm00001eb286730 | **CPN60-A07(Chr6-)** | Chr6:153147110-153151531 | - | A | 572 | 60,53 | 4,93 | 4421 | 1 |
| *103* | Zm00001eb294310 | **CPN60-A08(Chr6-)** | Chr6:173220518-173225900 | - | A | 580 | 61,27 | 6,11 | 5382 | 5 |
| *104* | Zm00001eb378930 | **CPN60-A09(Chr9+)** | Chr9:027938604-027943801 | + | A | 579 | 61,79 | 5,35 | 5176 | 5 |
| *105* | Zm00001eb001240 | **CPN60-B01(Chr1+)** | Chr1:003903971-003908178 | + | B | 502 | 54,13 | 6,45 | 4207 | 2 |
| *106* | Zm00001eb008400 | **CPN60-B02(Chr1-)** | Chr1:025489652-025496517 | - | B | 558 | 60,74 | 6,02 | 6865 | 1 |
| *107* | Zm00001eb111720 | **CPN60-B03(Chr2+)** | Chr2:224399017-224403831 | + | B | 535 | 59,10 | 5,96 | 4814 | 2 |
| *108* | Zm00001eb114320 | **CPN60-B04(Chr2+)** | Chr2:232913959-232918936 | + | B | 548 | 58,99 | 5,17 | 4831 | 2 |
| *109* | Zm00001eb118030 | **CPN60-B05(Chr2+)** | Chr2:242070765-242075991 | + | B | 535 | 59,08 | 5,42 | 5226 | 4 |
| *110* | Zm00001eb127430 | **CPN60-B06(Chr3+)** | Chr3:034728773-034735203 | + | B | 525 | 57,4 | 5,39 | 6430 | 1 |
| *111* | Zm00001eb189870 | **CPN60-B07(Chr4-)** | Chr4:170751022-170758167 | - | B | 535 | 57,65 | 6,43 | 7145 | 2 |
| *112* | Zm00001eb192630 | **CPN60-B08(Chr4-)** | Chr4:179991666-179997230 | - | B | 545 | 59,20 | 5,59 | 5564 | 1 |
| *113* | Zm00001eb200400 | **CPN60-B09(Chr4-)** | Chr4:207674486-207679216 | - | B | 406 | 58,91 | 5,31 | 4730 | 2 |
| *114* | Zm00001eb204950 | **CPN60-B10(Chr4-)** | Chr4:237245588-237256529 | - | B | 558 | 60,70 | 5,92 | 10941 | 1 |
| *115* | Zm00001eb207780 | **CPN60-B11(Chr4-)** | Chr4:245629316-245634717 | - | B | 560 | 60,31 | 6,61 | 5225 | 5 |
| *116* | Zm00001eb257580 | **CPN60-B12(Chr5+)** | Chr5:221541900-221547838 | + | B | 545 | 59,27 | 5,66 | 5938 | 1 |
| *117* | Zm00001eb298950 | **CPN60-B13(Chr7-)** | Chr7:002642329-002648187 | - | B | 535 | 59,01 | 5,38 | 5858 | 1 |
| *118* | Zm00001eb333890 | **CPN60-B14(Chr8-)** | Chr8:006629128-006639874 | - | B | 525 | 57,38 | 5,31 | 10470 | 3 |
| *119* | Zm00001eb017830 | **CPN10-A01(Chr1-)** | Chr1:063355095-063357831 | - | A | 97 | 10,58 | 6,93 | 2736 | 1 |
| *120* | Zm00001eb024030 | **CPN10-A02(Chr1+)** | Chr1:097502866-097504964 | + | A | 134 | 14,43 | 5,95 | 2098 | 1 |
| *121* | Zm00001eb071630 | **CPN10-A03(Chr2-)** | Chr2:012333485-012335877 | - | A | 117 | 12,49 | 6,64 | 2392 | 1 |
| *122* | Zm00001eb110270 | **CPN10-A04(Chr2+)** | Chr2:220419444-220421627 | + | A | 99 | 10,61 | 6,71 | 2183 | 1 |
| *123* | Zm00001eb290400 | **CPN10-A05(Chr6+)** | Chr6:164665009-164665577 | + | A | 98 | 10,51 | 6,71 | 568 | 1 |
| *124* | Zm00001eb329030 | **CPN10-A06(Chr7+)** | Chr7:178343373-178345376 | + | A | 98 | 10,54 | 8,06 | 2003 | 1 |
| *125* | Zm00001eb390720 | **CPN10-A07(Chr9-)** | Chr9:118929001-118931547 | - | A | 135 | 14,44 | 6,53 | 2546 | 1 |
| *126* | Zm00001eb100100 | **CPN10-B01(Chr2-)** | Chr2:188984228-188989465 | - | B | 245 | 25,58 | 8,15 | 5225 | 2 |
| *127* | Zm00001eb193040 | **CPN10-B02(Chr4+)** | Chr4:181556786-181558848 | + | B | 254 | 26,41 | 6,75 | 2062 | 3 |
| *128* | Zm00001eb373960 | **CPN10-B03(Chr9-)** | Chr9:012409089-012414124 | - | B | 194 | 25,76 | 7,87 | 5002 | 8 |
| *129* | Zm00001eb012470 | **HSP70-A01(Chr1+)** | Chr1:041027185-041032789 | + | A | 649 | 71,15 | 4,96 | 2604 | 1 |
| *130* | Zm00001eb012510 | **HSP70-A02(Chr1-)** | Chr1:041142629-041149958 | - | A | 540 | 59,42 | 5,90 | 4329 | 1 |
| *131* | Zm00001eb029200 | **HSP70-A03(Chr1+)** | Chr1:157756210-157760355 | + | A | 649 | 71,10 | 5,03 | 4145 | 3 |
| *132* | Zm00001eb043470 | **HSP70-A04(Chr1+)** | Chr1:228596659-228601178 | + | A | 678 | 72,67 | 5,43 | 4519 | 1 |
| *133* | Zm00001eb053750 | **HSP70-A05(Chr1+)** | Chr1:269789451-269797981 | + | A | 683 | 73,03 | 5,04 | 5530 | 1 |
| *134* | Zm00001eb101990 | **HSP70-A06(Chr2-)** | Chr2:195376595-195382949 | - | A | 681 | 72,76 | 5,50 | 3354 | 1 |
| *135* | Zm00001eb133480 | **HSP70-A07(Chr3+)** | Chr3:099949226-099957992 | + | A | 703 | 74,50 | 4,98 | 8460 | 3 |
| *136* | Zm00001eb136490 | **HSP70-A08(Chr3-)** | Chr3:127901534-127908631 | - | A | 649 | 71,17 | 5,00 | 4097 | 2 |
| *137* | Zm00001eb148420 | **HSP70-A09(Chr3-)** | Chr3:186198894-186202456 | - | A | 648 | 70,85 | 5,00 | 3562 | 2 |
| *138* | Zm00001eb165400 | **HSP70-A10(Chr4+)** | Chr4:003530483-003533031 | + | A | 515 | 56,82 | 5,40 | 2548 | 2 |
| *139* | Zm00001eb209550 | **HSP70-A11(Chr4+)** | Chr4:248765270-248769422 | + | A | 663 | 73,14 | 5,04 | 4152 | 1 |
| *140* | Zm00001eb214940 | **HSP70-A12(Chr5-)** | Chr5:007794462-007796634 | - | A | 592 | 65,70 | 5,52 | 2172 | 2 |
| *141* | Zm00001eb217010 | **HSP70-A13(Chr5-)** | Chr5:013121936-013130573 | - | A | 683 | 73,24 | 5,01 | 5637 | 1 |
| *142* | Zm00001eb220140 | **HSP70-A14(Chr5+)** | Chr5:022643552-022649333 | + | A | 678 | 72,70 | 5,50 | 5781 | 2 |
| *143* | Zm00001eb229930 | **HSP70-A15(Chr5-)** | Chr5:071317948-071322177 | - | A | 663 | 73,10 | 5,01 | 4049 | 2 |
| *144* | Zm00001eb351340 | **HSP70-A16(Chr8-)** | Chr8:119215374-119218426 | - | A | 649 | 70,97 | 5,10 | 3052 | 3 |
| *145* | Zm00001eb368000 | **HSP70-A17(Chr8-)** | Chr8:175434894-175438173 | - | A | 648 | 70,89 | 4,98 | 3279 | 1 |
| *146* | Zm00001eb397600 | **HSP70-A18(Chr9+)** | Chr9:145591040-145594173 | + | A | 652 | 71,51 | 5,12 | 3133 | 1 |
| *147* | Zm00001eb400020 | **HSP70-A19(Chr9+)** | Chr9:152764309-152767655 | + | A | 578 | 62,48 | 5,47 | 3346 | 1 |
| *148* | Zm00001eb409950 | **HSP70-A20(Chr10+)** | Chr10:022003764-022012203 | + | A | 703 | 74,68 | 4,98 | 5439 | 3 |
| *149* | Zm00001eb115870 | **HSP70-B01(Chr2-)** | Chr2:237240855-237244486 | - | B | 484 | 52,63 | 4,95 | 3199 | 3 |
| *150* | Zm00001eb188700 | **HSP70-B02(Chr4+)** | Chr4:166532569-166544015 | + | B | 899 | 98,96 | 5,41 | 8446 | 2 |
| *151* | Zm00001eb252410 | **HSP70-B03(Chr5+)** | Chr5:210089133-210100750 | + | B | 897 | 99,12 | 5,34 | 8617 | 3 |
| *152* | Zm00001eb224550 | **HSP70-C01(Chr5-)** | Chr5:042838363-042845259 | - | C | 750 | 82,91 | 5,32 | 6896 | 2 |
| *153* | Zm00001eb273690 | **HSP70-C02(Chr6-)** | Chr6:100177288-100187894 | - | C | 753 | 83,30 | 5,36 | 10415 | 4 |
| *154* | Zm00001eb283640 | **HSP70-C03(Chr6+)** | Chr6:141236744-141246333 | + | C | 841 | 93,05 | 5,04 | 6263 | 5 |
| *155* | Zm00001eb283800 | **HSP70-C04(Chr6-)** | Chr6:141862893-141874217 | - | C | 843 | 93,75 | 5,26 | 11149 | 2 |
| *156* | Zm00001eb346940 | **HSP70-C05(Chr8-)** | Chr8:091794623-091801084 | - | C | 833 | 92,32 | 5,10 | 6461 | 3 |
| *157* | Zm00001eb346960 | **HSP70-C06(Chr8+)** | Chr8:091925935-091936745 | + | C | 848 | 93,58 | 5,16 | 7810 | 3 |
| *158* | Zm00001eb101750 | **HSP90-A01(Chr2+)** | Chr2:194675915-194679753 | + | A | 698 | 80,18 | 4,93 | 3838 | 1 |
| *159* | Zm00001eb137900 | **HSP90-A02(Chr3+)** | Chr3:135890402-135900659 | + | A | 813 | 91,19 | 5,11 | 10257 | 1 |
| *160* | Zm00001eb199230 | **HSP90-A03(Chr4-)** | Chr4:203484540-203489254 | - | A | 787 | 88,68 | 4,98 | 4714 | 1 |
| *161* | Zm00001eb199590 | **HSP90-A04(Chr4-)** | Chr4:204474121-204478467 | - | A | 699 | 80,37 | 4,89 | 4346 | 2 |
| *162* | Zm00001eb228120 | **HSP90-A05(Chr5-)** | Chr5:063379671-063384937 | - | A | 808 | 92,88 | 4,82 | 5043 | 2 |
| *163* | Zm00001eb264470 | **HSP90-A06(Chr6+)** | Chr6:028646381-028677797 | + | A | 804 | 90,88 | 5,13 | 31416 | 5 |
| *164* | Zm00001eb272290 | **HSP90-A07(Chr6+)** | Chr6:093476676-093482297 | + | A | 804 | 92,58 | 4,86 | 5621 | 5 |
| *165* | Zm00001eb315880 | **HSP90-A08(Chr7-)** | Chr7:135311473-135316330 | - | A | 797 | 90,21 | 4,85 | 4836 | 2 |
| *166* | Zm00001eb316410 | **HSP90-A09(Chr7+)** | Chr7:137154149-137157876 | + | A | 699 | 80,28 | 4,96 | 3727 | 2 |
| *167* | Zm00001eb418610 | **HSP90-A10(Chr10+)** | Chr10:095651629-095657768 | + | A | 714 | 81,81 | 4,94 | 3139 | 1 |
| *168* | Zm00001eb034130 | **HSP90-B01(Chr1-)** | Chr1:188402249-188406911 | - | B | 351 | 40,60 | 4,89 | 4662 | 2 |
| *169* | Zm00001eb020330 | **HSP100-A01(Chr1+)** | Chr1:075277050-075290816 | + | A | 978 | 108,88 | 6,46 | 13548 | 2 |
| *170* | Zm00001eb084420 | **HSP100-A02(Chr2-)** | Chr2:061836490-061843899 | - | A | 932 | 101,32 | 7,51 | 7229 | 6 |
| *171* | Zm00001eb084890 | **HSP100-A03(Chr2+)** | Chr2:066025600-066032963 | + | A | 921 | 102,11 | 6,01 | 7363 | 1 |
| *172* | Zm00001eb234160 | **HSP100-A04(Chr5-)** | Chr5:095338002-095344275 | - | A | 988 | 109,57 | 5,90 | 6273 | 2 |
| *173* | Zm00001eb242420 | **HSP100-A05(Chr5+)** | Chr5:171243097-171248059 | + | A | 947 | 102,38 | 5,96 | 4962 | 2 |
| *174* | Zm00001eb293780 | **HSP100-A06(Chr6+)** | Chr6:172120888-172124712 | + | A | 912 | 101,14 | 5,65 | 3824 | 2 |
| *175* | Zm00001eb393250 | **HSP100-A07(Chr9+)** | Chr9:129727125-129734139 | + | A | 974 | 108,79 | 7,10 | 6610 | 2 |
| *176* | Zm00001eb411490 | **HSP100-A08(Chr10+)** | Chr10:036165092-036171873 | + | A | 921 | 102,06 | 6,01 | 6781 | 2 |
| *177* | Zm00001eb421360 | **HSP100-A09(Chr10-)** | Chr10:113641188-113648714 | - | A | 921 | 102,09 | 6,01 | 7526 | 2 |
| *178* | Zm00001eb086360 | **HSP100-B01(Chr2-)** | Chr2:082765095-082769925 | - | B | 1040 | 109,19 | 6,24 | 4830 | 2 |
| *179* | Zm00001eb178400 | **HSP100-B02(Chr4-)** | Chr4:074963137-074967644 | - | B | 1028 | 108,72 | 7,31 | 4507 | 1 |
| *180* | Zm00001eb197530 | **HSP100-B03(Chr4+)** | Chr4:196531671-196535092 | + | B | 838 | 90,01 | 6,99 | 3421 | 2 |
| *181* | Zm00001eb412800 | **HSP100-B04(Chr10+)** | Chr10:054420561-054424851 | + | B | 1023 | 108,38 | 7,44 | 4131 | 2 |
| *182* | Zm00001eb420410 | **HSP100-B05(Chr10-)** | Chr10:107604434-107609437 | - | B | 1030 | 109,23 | 5,76 | 5003 | 4 |

**Table S4** List of the 182 validated ZmHSPs, organized according to their multiple nomenclatures, regarded the different notations found at MaizeGDB, NCBI and Plaza, besides some arbitrary names used on papers and such:

| **#** | **Zm-B73-NAM-5.0** | **B73-GRAMENE-4.0** | **B73-RefGen_v3(5b+)** | **B73-RefGen_v2 (5b)** | **NCBI (Entrez Gene)** | **Plaza** | **Arbitrary** |
| --- | --- | --- | --- | --- | --- | --- | --- |
| *1* | Zm00001eb011880 | Zm00001d028555 | AC208204.3_FG006 | GRMZM2G481525 | LOC100274593 | - | HSP10 |
| *2* | Zm00001eb011900 | Zm00001d028557 | GRMZM2G046382 | GRMZM2G046382 | LOC103634220 | ZM01G10840 | HSP9-B |
| *3* | Zm00001eb011930 | Zm00001d028561 | GRMZM2G306679 | GRMZM2G306714 | LOC100191552 | ZM01G10870 | HSP11 |
| *4* | Zm00001eb124940 | Zm00001d039933 | GRMZM2G437100 | GRMZM5G857693 | LOC100280576 | ZM03G05680 | EU952685 |
| *5* | Zm00001eb124960 | Zm00001d039936 | GRMZM2G158232 | GRMZM2G158232 | LOC100286044 | ZM03G05710 | HSP12 |
| *6* | Zm00001eb335430 | Zm00001d008577 | GRMZM2G333635 | GRMZM2G333635 | LOC103634805 | ZM08G03190 | HSP1-1 |
| *7* | Zm00001eb397940 | Zm00001d047841 | GRMZM2G479260 | GRMZM2G479260 | LOC100283766 | ZM09G24060 | HSP17,4-2 |
| *8* | Zm00001eb121900 | Zm00001d039566 | GRMZM2G083810 | GRMZM2G083810 | LOC542723 | ZM03G02810 | HSP18F |
| *9* | Zm00001eb257130 | Zm00001d018298 | GRMZM2G098167 | GRMZM2G098167 | LOC100191598 | ZM05G42580 | HSP28 |
| *10* | Zm00001eb311990 | Zm00001d020390 | GRMZM2G085934 | GRMZM2G085934 | LOC118473030 | ZM07G13420 | HSP17,5 |
| *11* | Zm00001eb337690 | Zm00001d008841 | GRMZM2G034157 | GRMZM2G034157 | LOC100037778 | ZM08G05110 | HSP18C-2 |
| *12* | Zm00001eb395330 | Zm00001d047542 | GRMZM2G012455 | GRMZM2G311710 | LOC100383105 | ZM09G21700 | HSP17,4-1 |
| *13* | Zm00001eb395360 | Zm00001d047548 | GRMZM2G404249 | GRMZM2G404274 | LOC542293 | ZM09G21760 | HSP18A |
| *14* | Zm00001eb395370 | Zm00001d047553 | GRMZM5G899188 | GRMZM5G899188 | LOC103639183 | ZM09G21780 | EU964864 |
| *15* | Zm00001eb081770 | Zm00001d003554 | GRMZM2G331701 | GRMZM2G331701 | LOC100284772 | ZM02G14200 | HSP22-C |
| *16* | Zm00001eb112330 | Zm00001d007271 | GRMZM2G375517 | GRMZM2G375517 | LOC100282088 | ZM02G41720 | HSP24 |
| *17* | Zm00001eb125010 | Zm00001d039941 | GRMZM2G413897 | GRMZM2G413897 | LOC100285576 | ZM03G05740 | HSP20-like-D |
| *18* | Zm00001eb125030 | Zm00001d039942 | GRMZM2G049767 | GRMZM2G049767 | LOC100282956 | ZM03G05750 | HSP16,9-3 |
| *19* | Zm00001eb208810 | Zm00001d053965 | GRMZM2G324956 | GRMZM2G324956 | LOC103654949 | ZM04G40730 | HSP 22-B |
| *20* | Zm00001eb252460 | Zm00001d017813 | GRMZM2G481605 | GRMZM2G481605 | LOC100285269 | ZM05G38280 | HSP17,4-3 |
| *21* | Zm00001eb291920 | Zm00001d038608 | GRMZM2G013970 | GRMZM2G013970 | LOC100275360 | ZM06G27710 | HSP21,7-6 |
| *22* | Zm00001eb352720 | Zm00001d010693 | GRMZM2G010927 | GRMZM2G010927 | LOC100278747 | ZM08G18850 | HSP20-like-N |
| *23* | Zm00001eb371530 | Zm00001d044728 | GRMZM2G335242 | GRMZM2G335242 | LOC100284139 | ZM09G00140 | HSP27 |
| *24* | Zm00001eb423300 | Zm00001d025508 | GRMZM2G346839 | GRMZM5G858128 | LOC100283239 | ZM10G17370 | HSP20 |
| *25* | Zm00001eb010530 | Zm00001d028408 | GRMZM2G149647 | GRMZM2G149647 | LOC542576 | ZM01G09580 | HSP26 |
| *26* | Zm00001eb026540 | Zm00001d030317 | GRMZM2G109814 | GRMZM2G109814 | LOC100284367 | ZM01G24290 | HSP20-like-F |
| *27* | Zm00001eb026680 | Zm00001d030345 | AC210517.3_FG003 | AC210517.3_FG003 | LOC100193370 | - | HSP20-like-L |
| *28* | Zm00001eb026690 | Zm00001d030346 | AC202185.4_FG004 | GRMZM5G849455 | LOC100283821 | - | HSP20-like-K |
| *29* | Zm00001eb032310 | Zm00001d031128 | GRMZM2G149751 | GRMZM2G449274 | LOC100383840 | ZM01G29940 | METS-1 |
| *30* | Zm00001eb034050 | Zm00001d031325 | GRMZM2G080724 | GRMZM2G080724 | LOC100281798 | ZM01G26930 | HSP8 |
| *31* | Zm00001eb047490 | Zm00001d032893 | GRMZM2G352627 | GRMZM2G352627 | LOC100285715 | ZM01G43830 | HSP20-like-J |
| *32* | Zm00001eb052730 | Zm00001d033478 | GRMZM2G081822 | GRMZM2G081822 | LOC100383047 | ZM01G48440 | HSP20-like-E |
| *33* | Zm00001eb124950 | Zm00001d039935 | GRMZM2G422240 | GRMZM5G849535 | LOC103649807 | ZM03G05700 | HSP17,2 |
| *34* | Zm00001eb193940 | Zm00001d052194 | GRMZM2G007729 | GRMZM2G007729 | LOC542602 | ZM04G27350 | HSP22-A |
| *35* | Zm00001eb218280 | Zm00001d013641 | GRMZM2G037146 | GRMZM2G037146 | LOC100382094 | ZM05G07180 | HSP20-like-A |
| *36* | Zm00001eb218310 | Zm00001d013643 | AC191676.3_FG011 | AC191676.3_FG011 | LOC103626029 | - | HSP20-like-O |
| *37* | Zm00001eb222770 | Zm00001d014146 | GRMZM2G465723 | GRMZM2G465723 | LOC100284197 | ZM05G10950 | HSP20-like-I |
| *38* | Zm00001eb222820 | Zm00001d014149 | GRMZM2G126972 | GRMZM2G126972 | LOC100193100 | ZM05G10970 | HSP20-like-H2 |
| *39* | Zm00001eb235800 | Zm00001d015777 | GRMZM5G803365 | GRMZM5G803365 | LOC100283886 | ZM05G23100 | HSP29 |
| *40* | Zm00001eb297350 | Zm00001d039169 | GRMZM2G099529 | GRMZM2G099626 | LOC100284584 | ZM06G32370 | HSP20-like-M |
| *41* | Zm00001eb322740 | Zm00001d021634 | GRMZM2G429396 | GRMZM2G429396 | LOC103633231 | ZM07G23030 | HSP30 |
| *42* | Zm00001eb372830 | Zm00001d044874 | GRMZM2G135960 | GRMZM2G135960 | LOC100276133 | ZM09G01130 | HSP23,6-mit |
| *43* | Zm00001eb050280 | Zm00001d033210 | GRMZM2G364069 | GRMZM2G364069 | LOC100191411 | ZM01G46340 | CDJ2-B |
| *44* | Zm00001eb060050 | Zm00001d034368 | GRMZM2G134917 | GRMZM2G134917 | LOC100382437 | ZM01G55470 | CDJ1 |
| *45* | Zm00001eb091890 | Zm00001d004840 | GRMZM2G029079 | GRMZM2G497058 | - | ZM02G23810 | DNAJ-p17 |
| *46* | Zm00001eb103670 | Zm00001d006230 | GRMZM2G433854 | GRMZM2G433854 | LOC103647623 | ZM02G34230 | DNAJ-p14 |
| *47* | Zm00001eb107580 | Zm00001d006666 | GRMZM2G337845 | GRMZM2G337845 | - | ZM02G37550 | DNAJ-p26 |
| *48* | Zm00001eb172900 | Zm00001d049583 | GRMZM2G091811 | AC216262.2_FG001 | LOC100382361 | ZM04G07810 | DNAJ-A6-chl |
| *49* | Zm00001eb192080 | Zm00001d052001 | GRMZM2G054076 | GRMZM2G054076 | LOC100280316 | ZM04G25650 | IDP4515 |
| *50* | Zm00001eb213310 | Zm00001d013111 | GRMZM2G028218 | GRMZM2G028218 | LOC100283585 | ZM05G02950 | DNAJ-p8 |
| *51* | Zm00001eb218560 | Zm00001d013669 | GRMZM2G134980 | GRMZM2G134980 | LOC100280317 | ZM05G07410 | CDJ3-B |
| *52* | Zm00001eb249430 | Zm00001d017476 | GRMZM2G118731 | GRMZM2G553232 | LOC100216704 | ZM05G35510 | UMC2639 |
| *53* | Zm00001eb258200 | Zm00001d018413 | GRMZM2G092632 | GRMZM2G536644 | LOC100191825 | ZM05G43490 | CL8519-1A |
| *54* | Zm00001eb269190 | Zm00001d035975 | GRMZM2G473367 | GRMZM2G473367 | LOC100282679 | ZM06G07220 | GFA2-mit-1 |
| *55* | Zm00001eb294320 | - | GRMZM5G851710 | GRMZM5G851710 | - | - | IDP8326 |
| *56* | Zm00001eb379790 | Zm00001d045645 | GRMZM2G434839 | GRMZM2G434839 | LOC103639869 | ZM09G07210 | DNAJ-p12 |
| *57* | Zm00001eb392350 | Zm00001d047195 | GRMZM2G013934 | GRMZM2G013934 | - | ZM09G18890 | CL825X-3C |
| *58* | Zm00001eb404330 | Zm00001d048538 | GRMZM2G354746 | GRMZM2G354746 | LOC103640467 | ZM09G29650 | DNAJ-p13 |
| *59* | Zm00001eb407790 | Zm00001d023537 | GRMZM2G118316 | GRMZM2G418621 | LOC100304399 | ZM10G02730 | GFA2-mit-2 |
| *60* | Zm00001eb416450 | Zm00001d024635 | GRMZM5G856084 | GRMZM5G856084 | LOC103641303 | ZM10G10800 | IDP6583 |
| *61* | Zm00001eb429820 | Zm00001d026185 | - | - | LOC109942943 | - | DNAJ-p18 |
| *62* | Zm00001eb009060 | Zm00001d028256 | GRMZM2G040561 | GRMZM2G040561 | LOC100382193 | ZM01G08300 | IDP4752A |
| *63* | Zm00001eb042680 | Zm00001d032347 | GRMZM2G047153 | GRMZM2G047153 | LOC100193031 | ZM01G39460 | DNAJ-p33 |
| *64* | Zm00001eb118880 | Zm00001d039259 | GRMZM5G835677 | GRMZM5G835677 | LOC100194306 | ZM03G00270 | DNAJ-p21 |
| *65* | Zm00001eb208880 | Zm00001d053972 | GRMZM2G069603 | GRMZM2G069603 | LOC100282146 | ZM04G40790 | DNAJ-B4-2 |
| *66* | Zm00001eb240640 | Zm00001d016440 | GRMZM2G137495 | GRMZM2G137495 | LOC100191212 | ZM05G27910 | PCO112909-B |
| *67* | Zm00001eb282660 | Zm00001d037586 | GRMZM2G129218 | GRMZM2G129218 | LOC103630059 | ZM06G19420 | ERDJ3B |
| *68* | Zm00001eb288550 | Zm00001d038250 | GRMZM2G175860 | GRMZM2G175860 | LOC100273460 | ZM06G24700 | DNAJ-p10 |
| *69* | Zm00001eb295770 | Zm00001d039000 | GRMZM2G039886 | GRMZM2G039886 | LOC100282636 | ZM06G31050 | DNAJ-p9 |
| *70* | Zm00001eb339480 | Zm00001d009027 | GRMZM2G372870 | GRMZM2G372870 | LOC100282723 | ZM08G06590 | DNAJ-iso1 |
| *71* | Zm00001eb340880 | Zm00001d009210 | GRMZM2G108259 | GRMZM2G108259 | LOC100381538 | ZM08G07970 | DNAJ-p23 |
| *72* | Zm00001eb343630 | Zm00001d009556 | GRMZM2G119316 | GRMZM2G419643 | LOC100382308 | ZM08G10470 | DNAJ-p19 |
| *73* | Zm00001eb345630 | Zm00001d009783 | GRMZM2G086964 | GRMZM2G086964 | LOC103635449 | ZM08G12140 | CL10549-1B |
| *74* | Zm00001eb354940 | Zm00001d010958 | GRMZM2G119483 | GRMZM2G119483 | LOC100283703 | ZM08G20740 | DNAJ-B13 |
| *75* | Zm00001eb366410 | Zm00001d012242 | GRMZM2G128248 | GRMZM2G128248 | LOC100282402 | ZM08G30610 | DNAJ-p31 |
| *76* | Zm00001eb415590 | Zm00001d024527 | GRMZM2G171187 | GRMZM2G171187 | LOC103641254 | ZM10G09970 | DNAJ-B4-1 |
| *77* | Zm00001eb035420 | Zm00001d031490 | GRMZM2G141784 | GRMZM2G141784 | LOC100282737 | ZM01G32890 | DNAJ-10-B |
| *78* | Zm00001eb243690 | Zm00001d016810 | GRMZM2G119566 | GRMZM2G119566 | LOC100381679 | ZM05G30580 | DNAJ-p1 |
| *79* | Zm00001eb294570 | Zm00001d038879 | GRMZM2G175089 | GRMZM2G175089 | LOC100273210 | ZM06G30060 | DNAJ-10-A |
| *80* | Zm00001eb317080 | Zm00001d020973 | GRMZM2G168397 | GRMZM2G168397 | LOC100284051 | ZM07G18200 | PCO134240-B |
| *81* | Zm00001eb342590 | Zm00001d009436 | GRMZM2G119722 | GRMZM2G119722 | LOC100192576 | ZM08G09640 | CL2587-2B |
| *82* | Zm00001eb361030 | Zm00001d011668 | GRMZM2G469901 | GRMZM2G469901 | LOC103636214 | ZM08G26250 | DNAJ-p2 |
| *83* | Zm00001eb409280 | Zm00001d023721 | GRMZM2G130127 | GRMZM2G130127 | LOC100383616 | ZM10G04010 | JJJ1-2 |
| *84* | Zm00001eb409300 | Zm00001d023722 | GRMZM2G170027 | GRMZM2G585033 | LOC103640894 | ZM10G04020 | JJJ1-3 |
| *85* | Zm00001eb409350 | Zm00001d023723 | GRMZM2G047889 | GRMZM2G047889 | LOC103640901 | ZM10G04070 | JJJ1-1 |
| *86* | Zm00001eb409390 | Zm00001d023728 | GRMZM2G302883 | GRMZM2G302882 | LOC100277078 | ZM10G04090 | JJJ1-like-1 |
| *87* | Zm00001eb137150 | Zm00001d041627 | GRMZM2G005753 | GRMZM2G005753 | LOC100282943 | ZM03G17500 | DNAJ-p15 |
| *88* | Zm00001eb146070 | Zm00001d042653 | GRMZM2G049373 | GRMZM2G049373 | LOC100192790 | ZM03G25360 | DNAJ-p22 |
| *89* | Zm00001eb162560 | Zm00001d044528 | GRMZM2G064031 | GRMZM2G064031 | LOC103651627 | ZM03G40290 | UMC1641 |
| *90* | Zm00001eb205970 | Zm00001d053667 | GRMZM2G075485 | GRMZM2G075485 | LOC100273764 | ZM04G38160 | DNAJ-p6 |
| *91* | Zm00001eb236340 | Zm00001d015839 | GRMZM2G129987 | GRMZM2G129987 | LOC100284400 | ZM05G23530 | ATJ6 |
| *92* | Zm00001eb254160 | Zm00001d017995 | GRMZM2G140070 | GRMZM2G140070 | LOC100382440 | ZM05G39890 | CL18199-2 |
| *93* | Zm00001eb279930 | Zm00001d037257 | GRMZM2G009826 | GRMZM2G009826 | LOC100283349 | - | DNAJ-p16 |
| *94* | Zm00001eb342290 | Zm00001d009401 | GRMZM2G048611 | GRMZM2G048611 | LOC100272688 | ZM08G09350 | CL11333-1 |
| *95* | Zm00001eb346620 | Zm00001d009912 | GRMZM2G351775 | GRMZM2G351775 | LOC103635523 | ZM08G13040 | CL14391-1 |
| *96* | Zm00001eb010540 | Zm00001d028409 | GRMZM2G042253 | GRMZM2G042253 | LOC103633537 | ZM01G09590 | CPN60-β4-chl |
| *97* | Zm00001eb046580 | Zm00001d032789 | GRMZM2G416120 | GRMZM2G416120 | LOC542707 | ZM01G43100 | HSP60-2 |
| *98* | Zm00001eb065160 | Zm00001d034919 | GRMZM2G434173 | GRMZM5G808367 | LOC100281701 | ZM01G60140 | PCO140434 |
| *99* | Zm00001eb209960 | Zm00001d054089 | GRMZM2G015989 | GRMZM2G015989 | LOC100280498 | ZM04G41680 | TCP1-iso1 |
| *100* | Zm00001eb222300 | Zm00001d014090 | GRMZM2G111477 | GRMZM2G458208 | LOC542736 | ZM05G10550 | HSP60-1 |
| *101* | Zm00001eb268910 | Zm00001d035937 | GRMZM2G095252 | GRMZM2G133698 | LOC103629384 | ZM06G06960 | CPN60-β2-2 |
| *102* | Zm00001eb286730 | Zm00001d000399 | AC215201.3_FG005 | AC215201.3_FG005 | LOC103630297 | - | CPS2 |
| *103* | Zm00001eb294310 | Zm00001d038857 | GRMZM2G074790 | GRMZM5G898025 | LOC100272259 | ZM06G29830 | MMP50 |
| *104* | Zm00001eb378930 | Zm00001d045544 | GRMZM2G083716 | GRMZM2G083716 | LOC542185 | ZM09G06360 | CPN60-β2-1 |
| *105* | Zm00001eb001240 | Zm00001d027371 | GRMZM2G085909 | GRMZM2G085909 | LOC100381530 | ZM01G01180 | TCP1-D |
| *106* | Zm00001eb008400 | Zm00001d028183 | GRMZM2G069765 | GRMZM2G069765 | LOC100285961 | ZM01G07690 | PCO073966 |
| *107* | Zm00001eb111720 | Zm00001d007192 | GRMZM2G109425 | GRMZM2G109425 | LOC100193477 | ZM02G41130 | TCP1-Ζ |
| *108* | Zm00001eb114320 | Zm00001d007508 | GRMZM2G083095 | GRMZM2G083095 | LOC100382493 | ZM02G43700 | CL8405-1a |
| *109* | Zm00001eb118030 | Zm00001d007960 | GRMZM2G070542 | GRMZM2G070542 | LOC100282671 | ZM02G47250 | PCO099347 |
| *110* | Zm00001eb127430 | Zm00001d040256 | EF517601.1_FG012 | EF517601.1_FG012 | LOC100191320 | - | CCT-β2 |
| *111* | Zm00001eb189870 | Zm00001d051742 | GRMZM2G122767 | GRMZM2G122767 | LOC100191456 | ZM04G23780 | UMC1329 |
| *112* | Zm00001eb192630 | Zm00001d052056 | GRMZM2G039263 | GRMZM5G801722 | LOC100283716 | ZM04G26180 | CCT-α1 |
| *113* | Zm00001eb200400 | Zm00001d052948 | GRMZM2G381744 | GRMZM2G381744 | LOC103654524 | ZM04G32700 | CL8405-1b |
| *114* | Zm00001eb204950 | Zm00001d053553 | GRMZM2G175510 | GRMZM2G363779 | LOC103654781 | ZM04G37180 | TCP1-G |
| *115* | Zm00001eb207780 | Zm00001d053857 | GRMZM2G009871 | GRMZM2G009871 | LOC100272855 | ZM04G39760 | TCP1-H |
| *116* | Zm00001eb257580 | Zm00001d018349 | GRMZM2G110626 | GRMZM2G110626 | LOC100272316 | ZM05G42930 | CCT-α2 |
| *117* | Zm00001eb298950 | Zm00001d018694 | GRMZM2G043383 | GRMZM2G043722 | LOC100272988 | ZM07G00880 | TCP1-Y |
| *118* | Zm00001eb333890 | Zm00001d008369 | GRMZM2G058276 | GRMZM2G058276 | LOC103634709 | ZM08G01530 | CL2646 |
| *119* | Zm00001eb017830 | Zm00001d029263 | GRMZM2G073401 | GRMZM2G073401 | LOC100282681 | ZM01G16260 | PCO063745 |
| *120* | Zm00001eb024030 | Zm00001d029997 | GRMZM2G050961 | GRMZM5G825144 | LOC100273606 | ZM01G21800 | CL7048-1a |
| *121* | Zm00001eb071630 | Zm00001d002408 | GRMZM2G090872 | GRMZM2G090872 | LOC103645976 | ZM02G05210 | B6U478 |
| *122* | Zm00001eb110270 | Zm00001d007031 | GRMZM2G035063 | GRMZM2G035063 | LOC103647892 | ZM02G39790 | CPN10-3 |
| *123* | Zm00001eb290400 | Zm00001d038456 | GRMZM2G410812 | GRMZM2G410815 | LOC100285797 | - | PCO066521-B |
| *124* | Zm00001eb329030 | Zm00001d022307 | GRMZM2G013652 | GRMZM2G013652 | LOC100193174 | ZM07G28670 | CPN10-2A |
| *125* | Zm00001eb390720 | Zm00001d047067 | GRMZM2G067495 | GRMZM2G067495 | LOC100381273 | - | CHL-CPN10 |
| *126* | Zm00001eb100100 | Zm00001d005812 | AC190999.2_FG004 | GRMZM2G399284 | LOC100216904 | - | CL21464-1 |
| *127* | Zm00001eb193040 | Zm00001d052101 | GRMZM2G091189 | GRMZM2G091189 | LOC100383971 | ZM04G26540 | CPN10-1 |
| *128* | Zm00001eb373960 | Zm00001d045025 | GRMZM2G127609 | GRMZM2G127609 | LOC100191248 | ZM09G02210 | CPN20-2 |
| *129* | Zm00001eb012470 | Zm00001d028630 | GRMZM2G428391 | GRMZM2G428391 | LOC100281806 | ZM01G11380 | HSP 70-6 |
| *130* | Zm00001eb012510 | Zm00001d028639 | GRMZM2G145275 | GRMZM2G145275 | LOC103634464 | ZM01G11410 | HSP 70-3A |
| *131* | Zm00001eb029200 | Zm00001d030725 | GRMZM2G056039 | GRMZM2G056039 | LOC103639924 | ZM01G26930 | HSP 70-1 |
| *132* | Zm00001eb043470 | - | - | - | LOC100191408 | - | DNAK-1 |
| *133* | Zm00001eb053750 | Zm00001d033591 | GRMZM2G079668 | GRMZM2G079668 | LOC100280354 | ZM01G49450 | HSP 70-2 |
| *134* | Zm00001eb101990 | Zm00001d006036 | GRMZM2G365374 | GRMZM2G365374 | LOC100286241 | ZM02G32820 | HSP 70-5 |
| *135* | Zm00001eb133480 | Zm00001d041119 | GRMZM2G453617 | GRMZM2G133044 | LOC103650436 | - | HSP 70-6-chl |
| *136* | Zm00001eb136490 | Zm00001d041550 | GRMZM2G340251 | GRMZM5G817178 | LOC103650526 | ZM03G16880 | HSP 70-4 |
| *137* | Zm00001eb148420 | Zm00001d042922 | AC209784.3_FG007 | AC209784.3_FG007 | LOC103651028 | - | HSP 6 |
| *138* | Zm00001eb165400 | Zm00001d048687 | GRMZM2G106429 | GRMZM2G106429 | LOC103652717 | ZM04G00910 | HSP 70-3B |
| *139* | Zm00001eb209550 | Zm00001d054043 | GRMZM2G415007 | GRMZM2G415007 | LOC732809 | ZM04G41380 | BIP2-A |
| *140* | Zm00001eb214940 | - | GRMZM2G471196 | GRMZM2G471196 | LOC103625886 | ZM05G04300 | BIP3 |
| *141* | Zm00001eb217010 | Zm00001d013507 | GRMZM2G111475 | GRMZM2G111475 | LOC103625973 | ZM05G06140 | HSP 70-17A |
| *142* | Zm00001eb220140 | Zm00001d013842 | GRMZM2G153815 | GRMZM2G153815 | LOC100285374 | ZM05G08800 | HSP 70-12 |
| *143* | Zm00001eb229930 | Zm00001d014993 | GRMZM2G114793 | GRMZM2G114793 | LOC732808 | ZM05G17320 | BIP1 |
| *144* | Zm00001eb351340 | Zm00001d010529 | GRMZM5G802801 | GRMZM5G802801 | LOC103635762 | ZM08G17610 | HSP 7 |
| *145* | Zm00001eb368000 | Zm00001d012420 | GRMZM2G310431 | GRMZM2G310431 | LOC100501536 | ZM08G32130 | HSP 1 |
| *146* | Zm00001eb397600 | Zm00001d047799 | GRMZM2G366532 | GRMZM2G366532 | LOC100272911 | ZM09G23740 | HSP 5 |
| *147* | Zm00001eb400020 | Zm00001d048073 | GRMZM2G024718 | GRMZM2G324499 | LOC100281706 | ZM09G25800 | HSP 70-8 |
| *148* | Zm00001eb409950 | Zm00001d023802 | GRMZM2G001500 | GRMZM2G001567 | LOC100286168 | ZM10G04570 | HSP 70-22 |
| *149* | Zm00001eb115870 | Zm00001d007706 | GRMZM2G066902 | GRMZM2G066902 | LOC100277908 | ZM02G45420 | UnchProt-52,6 |
| *150* | Zm00001eb188700 | Zm00001d051607 | GRMZM2G158093 | GRMZM2G319649 | LOC103653979 | ZM04G22500 | HSP 70-7 |
| *151* | Zm00001eb252410 | Zm00001d017809 | GRMZM2G020040 | GRMZM2G020040 | LOC100279899 | ZM05G38240 | HSP 70-9 |
| *152* | Zm00001eb224550 | Zm00001d014358 | GRMZM2G056766 | GRMZM2G056766 | LOC103626279 | ZM05G12480 | HSP 70-10 |
| *153* | Zm00001eb273690 | Zm00001d036571 | GRMZM2G016886 | GRMZM5G805493 | LOC103629632 | ZM06G11470 | HSP 70-16B |
| *154* | Zm00001eb283640 | Zm00001d037700 | GRMZM2G023232 | GRMZM2G023232 | LOC100217188 | ZM06G20290 | HSP 70-14 |
| *155* | Zm00001eb283800 | Zm00001d037717 | GRMZM2G361605 | GRMZM5G822995 | LOC103630125 | ZM06G20480 | HSP 23 |
| *156* | Zm00001eb346940 | Zm00001d009948 | GRMZM2G001358 | GRMZM2G148870 | LOC100281415 | - | HSP 70-15 |
| *157* | Zm00001eb346960 | Zm00001d009950 | GRMZM2G063676 | GRMZM5G886850 | LOC100285213 | ZM08G13320 | HSP 70-16A |
| *158* | Zm00001eb101750 | Zm00001d006008 | GRMZM2G112165 | GRMZM2G112165 | LOC103647533 | ZM02G32560 | HSP81-1 |
| *159* | Zm00001eb137900 | Zm00001d041719 | GRMZM2G047434 | GRMZM2G370470 | LOC100286003 | ZM03G18110 | HSP90-6 |
| *160* | Zm00001eb199230 | Zm00001d052809 | GRMZM2G002220 | GRMZM2G002220 | LOC100383793 | ZM04G31660 | HSP90-5 |
| *161* | Zm00001eb199590 | Zm00001d052855 | GRMZM2G012631 | GRMZM2G012631 | LOC100384478 | ZM04G32000 | HSP13 |
| *162* | Zm00001eb228120 | Zm00001d014792 | GRMZM2G399073 | GRMZM2G399073 | LOC100279926 | ZM05G15800 | SHPL1 |
| *163* | Zm00001eb264470 | Zm00001d035285 | AC234183.1_FG002 | AC234183.1_FG002 | LOC103629136 | - | HSP90-6-mit |
| *164* | Zm00001eb272290 | Zm00001d036401 | GRMZM2G141931 | GRMZM2G141931 | LOC100285108 | ZM06G10080 | SHPL2 |
| *165* | Zm00001eb315880 | Zm00001d020827 | GRMZM5G813217 | GRMZM5G813217 | LOC100279992 | ZM07G17050 | HSP90-5-chl |
| *166* | Zm00001eb316410 | Zm00001d020898 | GRMZM2G069651 | GRMZM2G069651 | LOC100272368 | ZM07G17580 | HSP4 |
| *167* | Zm00001eb418610 | Zm00001d024903 | GRMZM5G833699 | GRMZM5G833699 | LOC100196928 | ZM10G12980 | HSP90-1 |
| *168* | Zm00001eb034130 | Zm00001d031332 | GRMZM2G024668 | GRMZM2G024668 | LOC100384473 | ZM01G31580 | HSP90-2 |
| *169* | Zm00001eb020330 | Zm00001d029557 | GRMZM2G130121 | GRMZM2G333795 | LOC103636567 | ZM01G18450 | CLPB-3 |
| *170* | Zm00001eb084420 | Zm00001d003847 | GRMZM2G149567 | GRMZM5G883585 | LOC100383428 | ZM02G16440 | CLPD-2 |
| *171* | Zm00001eb084890 | Zm00001d003894 | GRMZM2G001084 | GRMZM2G373849 | LOC100502219 | ZM02G16920 | CLPN-1 |
| *172* | Zm00001eb234160 | Zm00001d015520 | GRMZM2G060561 | GRMZM2G060561 | LOC100217072 | ZM05G21370 | CLPB-4 |
| *173* | Zm00001eb242420 | Zm00001d016664 | GRMZM2G172230 | GRMZM2G172230 | LOC100383653 | ZM05G29550 | CLPD-1 |
| *174* | Zm00001eb293780 | Zm00001d038806 | GRMZM2G360681 | GRMZM2G360681 | LOC541780 | ZM06G29340 | HSP101-A |
| *175* | Zm00001eb393250 | Zm00001d047302 | GRMZM2G162968 | GRMZM5G857423 | LOC100193235 | ZM09G19730 | CLPB-p |
| *176* | Zm00001eb411490 | Zm00001d024009 | GRMZM2G009443 | GRMZM2G009443 | LOC100383416 | ZM10G06060 | CLPC-1 |
| *177* | Zm00001eb421360 | Zm00001d025273 | GRMZM2G123922 | GRMZM2G123922 | LOC103641592 | ZM10G15640 | CLPN-2 |
| *178* | Zm00001eb086360 | Zm00001d004094 | GRMZM2G359779 | GRMZM2G359779 | LOC103646632 | ZM02G18590 | SMAX1-LIKE-4-A |
| *179* | Zm00001eb178400 | Zm00001d050227 | GRMZM2G119696 | GRMZM2G119696 | LOC103653403 | ZM04G12800 | SUP-MAX2-2 |
| *180* | Zm00001eb197530 | Zm00001d052612 | GRMZM2G013128 | GRMZM2G013128 | LOC103654382 | ZM04G30190 | SMAX1-LIKE-3-B |
| *181* | Zm00001eb412800 | Zm00001d024187 | GRMZM2G117836 | GRMZM2G117836 | LOC103641087 | ZM10G07240 | SUP-MAX2-1 |
| *182* | Zm00001eb420410 | Zm00001d025136 | GRMZM2G032547 | GRMZM2G032547 | LOC103641545 | ZM10G14680 | SMAX1-LIKE-4-B |

**Table S5** List of the 182 validated ZmHSPs, organized according to their multiple nomenclatures, regarded the different notations found at UniProtKB, GenBank and other databases:

| **#** | **Zm-B73-NAM-5.0** | **UniProtKB** | **GenBank** | **Alternative Names** |
| --- | --- | --- | --- | --- |
| *1* | Zm00001eb011880 | A0A0F6P1Q3, B6TDB5 | ONL96363, DAA44560 | A0A317YLJ8, AC208204, AC217897, AC219047, B4G250, BM074312, BT016867, C0PLX5, ctg10, ctg11, EU949258, EU962980, EZ096968, HSP17,4-3, IDPC175, NM_001148946, NP_001142418 |
| *2* | Zm00001eb011900 | A0A1D6JXF4, KJ789381 | ONL96365, DAA44562 | CM007647, XP_008654024, ZmHSP17.4 |
| *3* | Zm00001eb011930 | A0A317YLI6, B4F976 | ONL96399, DAA44566 | A0A3L6DEF1, B6TLK8, BT033664, CM007647, EU961108 |
| *4* | Zm00001eb124940 | A0A1D6MLV8, B6SIX0 | ONM30193, DAA53575 | ACG24803, CM007649, EU952685, zma:100280576 |
| *5* | Zm00001eb124960 | B4G197 | ONM30198, DAA53578 | ACG40361, B6TTC8, DAA53578, EU968243, Q43701 |
| *6* | Zm00001eb335430 | K7VA55 | AQK90196, AFW79982 | AY109481, CC785247, cl2172, ctg326 |
| *7* | Zm00001eb397940 | B4FT59 | AQL07768, AFW88862 | BM075784, rs132552657, TIDP2749 |
| *8* | Zm00001eb121900 | B4F9K4 | ONM29253, DAA53125 | ACF78797, AY103645, BT016317, BT033792, CM007649, cMHSP18-9, EU960295, MHSP18-3, pZmHSP17.2, ttu1, uaz210, uwo10, X54076, zma:542723 |
| *9* | Zm00001eb257130 | B4F9E8 | AQK75501, AFW73777 | ACF78741, ACR38487, NP_001130499, zma:100191598 |
| *10* | Zm00001eb311990 | A0A1D6I3X6, B6U175 | DAA61337 | ACG43108, EU970990, ZM07G13420 |
| *11* | Zm00001eb337690 | P24632 | AFW80297 | HSP17,8-2, IDP2369, MHSP18-3, NP_001105954, zma:100037778 |
| *12* | Zm00001eb395330 | C0PD31 | AQL06966, AFW88482 | - |
| *13* | Zm00001eb395360 | Q08275 | AQL06974, AFW88488 | AC202069, AC204874, AC213518, AI854979, BT016978, BT070148, ctg386, EU942728, EU952864, EU965727, GRMZM2G056857, HSP17,5-2, IDP1957, magi5272, NP_001105352, S59779, uwo9, XP_008660193, zma:542293, ZmHSP18-1 |
| *14* | Zm00001eb395370 | Q08275 | AQL06974, AFW88490 | ACG36982, B6SJE9, EU964864, ZM09G21780 |
| *15* | Zm00001eb081770 | A0A3L6G620, C0P8J4 | ONM17149, DAA37620 | A0A3L6FWF1, B6TG53, B6TXB5, rs131341018 |
| *16* | Zm00001eb112330 | A0A1D6F559, B6T3F5 | ONM26430, DAA41888 | A0A0C4UTI1, A0A3L6FTK1, CM007648 |
| *17* | Zm00001eb125010 | B6U6V5 | ONM30202, DAA53581 | - |
| *18* | Zm00001eb125030 | B6SLV6 | ONM30203, DAA53582 | B6TD78, LOC100282956, NM_001155861, NP_00114933 |
| *19* | Zm00001eb208810 | A0A3L6F6L4, K7UST5 | AQK60868, AFW66666 | B6TVT8, CK347788, ctg202, LOC100284632, NM_001157527, NP_001150999, rs129966963, umc2656 |
| *20* | Zm00001eb252460 | A0A1D6HI24, B6U2K9 | AQK74160, AFW73076 | NM_001158163, NP_001151635 |
| *21* | Zm00001eb291920 | B6SRT5 | AQK87024, AFW78566 | NM_001149453, NP_001142925 |
| *22* | Zm00001eb352720 | B6UD04 | AQK94610, AFW82416 | - |
| *23* | Zm00001eb371530 | K7VQ98 | AQL00739, AFW85157 | B6TQD6 |
| *24* | Zm00001eb423300 | K7TTJ0 | AQK43884, AFW58362 | rs132587893 |
| *25* | Zm00001eb010530 | A0A317Y2K5, Q41815 | ONL95913, DAA44366 | AC177947, AC193470, AC211263, B7ZEQ0, C0PP83, CC748126, CC825836, CM007647, EU951996, EU952790, EU964533, L28712 |
| *26* | Zm00001eb026540 | A0A1D6KBW7, B6TSR7 | ONM00795, DAA46729 | A0A317YEQ3, LOC100284367, NM_001157262, NP_001150734 |
| *27* | Zm00001eb026680 | A0A317Y6A5, B4FES7 | ONM00851, DAA46745 | B6T1R8, ACG31051, EU958933, NP_001064201 |
| *28* | Zm00001eb026690 | A0A1D6KC46, B6TM52 | ONM00852, DAA46746 | A0A317Y6A2, AI649666, AY104209, ctg29, LOC100283821, NM_001156719, NP_001150191, TC171944, UMC1461 |
| *29* | Zm00001eb032310 | A0A317Y093, C0PJF1 | ONM02231, DAA47593 | A0A1D6KGM6, AA030695, B4G1T4, C0P5Y3 |
| *30* | Zm00001eb034050 | A0A317Y0Z8, C0PPG1 | ONM02701, DAA47836 | B6SZ50, BT070180, CM007647 |
| *31* | Zm00001eb047490 | A0A1D6KUQ3, B6U8I5 | ONM06292, DAA49876 | A0A1D6KUQ2, A0A317Y5F2, LOC100285715, NM_001158606, NP_001152078 |
| *32* | Zm00001eb052730 | A0A1D6KZ63, C0PCL7 | ONM07666, DAA50620 | A0A317Y0J6, LOC100383047, NM_001175723, NP_001169194 |
| *33* | Zm00001eb124950 | A0A1D6MLV7, B4G197 | ONM30196, DAA53577 | A0A1D6MLV9, AC193322, AC207472, AC207795, AI944086, AW042291, AY109481, AY111665, BE123155, BE509732, CC197688, CC747159, CC789825, CL2172, CL13719, CM007649, ctg115, DQ244240, EU952538, EU968243, TC172835, TC178268, X65725 |
| *34* | Zm00001eb193940 | A0A3L6F5U7, O64960 | AQK56431, AFW64398 | ACG32582, AP003488, AY104434, B4FQS7, B6T649, CC784624, CC785510, ctg182, EU960464, HSP24,1-mit, MT019963, pco116068, umc2652, zma:542602, ZmHSP23.5 |
| *35* | Zm00001eb218280 | A0A1D6GL72, Q8W0Q8 | AQK64066, AFW68104 | C0P4A5, ZM05G07180 |
| *36* | Zm00001eb218310 | A0A1D6GL74 | AQK64068, AFW68107 | - |
| *37* | Zm00001eb222770 | B6TR54 | AQK65343, AFW68711 | NM_001157092, NP_001150564 |
| *38* | Zm00001eb222820 | B4FDS8 | AQK65348, AFW68715 | B8A1I4, LOC100280039, NM_001152981, NP_001146453 |
| *39* | Zm00001eb235800 | B6TMR8 | AQK69523, AFW70692 | ACG38401, EU966283, NM_001156784, NP_001150256, zma:100283886 |
| *40* | Zm00001eb297350 | A0A1D6ME89, B4F9Z4 | AQK88965, AFW79370 | K7UNA8 |
| *41* | Zm00001eb322740 | B6SRC0 | ONM57747, DAA62920 | ACG27403, EU955285, XM_008654929, XP_008653151 |
| *42* | Zm00001eb372830 | C4J5C1 | AQL01048, AFW85291 | ACR36371, BT086018 |
| *43* | Zm00001eb050280 | A0A1D6KWY2, B8A362 | ONM06976, DAA50285 | A0A1D6GLE1, A0A1D6GLE3, A0A1D6KIF8, A0A1D6KWY6, A0A317Y6H5, A0A3L6EII9, AA231897, AF053468, AI586871, AI615176, AI737639, AI977916, AW067282, AW261401, AW330853, AW506618, AW562755, b0611E16, BE129842, BT016805, BT016912, BT055206, BT088091, csu164, ctg57, DQ642431, EU949609, EU961250, EZ106800, O65160, T12748, T25244, TC169671, umc1968 |
| *44* | Zm00001eb060050 | A0A317YGS1, C0P7S8 | ONM10053, DAA51803 | A0A3L6EQ36, A0A3L6GB05, AC084296, AC191090, AC202525, AC205502, AY103727, c0083O09, c0090G18, c0449L11, CC146313, CC149579, CC738121, CC743650, CC740721, CC789880, CD988458, csu63, ctg38, ctg62, ctg92, EU973975, gnp_QAP4e06a, gpm394a, HSP40, pco082317, T12693, TC180588, umc343 |
| *45* | Zm00001eb091890 | A0A1D6EHR1 | ONM19663, DAA39063 | A0A3L6DW44, A0A3L6FSJ8, A0A3L6FV28, GRMZM2G029060 |
| *46* | Zm00001eb103670 | A0A1D6ETZ2 | ONM23140, DAA40640 | - |
| *47* | Zm00001eb107580 | A0A1D6EZH8 | ONM24679, DAA41207 | - |
| *48* | Zm00001eb172900 | A0A1D6PWA4, C0P6X3 | AQK50843, AFW61248 | A0A1D6PWA6, A0A3L6EXU9, A0A3L6G6I8 |
| *49* | Zm00001eb192080 | A0A1D6QBV5, B6U659 | AQK55720, AFW64107 | A0A1D6QBW2, A0A3L6F779, B8A360, IDP4515, K7U1A5 |
| *50* | Zm00001eb213310 | A0A1D6GG02, B6TJQ8 | AQK62482, AFW67421 | - |
| *51* | Zm00001eb218560 | A0A1D6GLE1, B8A362 | AQK64135, AFW68140 | A0A096S8H0, A0A1D6GLE3, A0A1D6GLE5, A0A1D6KWY5, AA231897, AY104862, BT017212, BT024164, BV079852, BV10654, csu164, ctg360, EU942141, EU949390, EZ055497, EZ107056, EZ107923, NM_001153243, rs130000312, rs131176955, T12748 |
| *52* | Zm00001eb249430 | A0A1D6HF19, B4FJC3 | AQK73236, AFW72634 | CC792315, CK347175, ctg247, umc2639 |
| *53* | Zm00001eb258200 | A0A1D6HNM7, B4FA93 | AQK75907, AFW73920 | A0A1D6HNN3, A0A1D6HNP0, AC211323, AFW73919, AY109726, B4FAB2, CC755627, CC827038, cl8519, ctg254 |
| *54* | Zm00001eb269190 | A0A1D6LK09, B4FVD3 | AQK80053, AFW75310 | AC203265, AP002837, AY105010, CC149145, CC786141, CC830168, ctg265, K7VBK5, pco077147 |
| *55* | Zm00001eb294320 | - | - | AC211579, AY112013, cl825-3, ctg287, ctg288, IDP8326 |
| *56* | Zm00001eb379790 | A0A1D6NY26, K7W7U2 | AQL02909, AFW86285 | - |
| *57* | Zm00001eb392350 | A0A1D6P7P5, K7VYH8 | AQL05853, AFW87992 | AY112013, CC784114, cl825-3c, ctg383 |
| *58* | Zm00001eb404330 | A0A1D6PM15, K7W3M2 | AQL10199, AFW89867 | - |
| *59* | Zm00001eb407790 | A0A1D6ITZ5, C0HFE1 | AQK39517, AFW56175 | A0A1D6IU00, AC190559, AY107146, BV083116, BV109583, BV109587, ctg393, pco098048, PZA01451, rs131175977, rs131185829, ss196417311 |
| *60* | Zm00001eb416450 | A0A1D6J0S9, K7U0W2 | AQK41670, AFW57376 | A0A1D6J0T0, A0A1D6J0T3, IDP6583 |
| *61* | Zm00001eb429820 | A0A1D6JD84 | AQK45787 | - |
| *62* | Zm00001eb009060 | A0A1D6JTM6, C0P5A0 | ONL95207, DAA44136 | A0A1D6GMR6, A0A1D6JTM5, A0A1D6JTM6, A0A1D6JTN0, A0A1D6QBW1, A0A317YJS5, magi15492, rs128404673 |
| *63* | Zm00001eb042680 | A0A317YG45, B4FDJ5 | ONM04906, DAA49176 | - |
| *64* | Zm00001eb118880 | A0A1D6MES0, B4FI78 | ONM28135, DAA52692 | - |
| *65* | Zm00001eb208880 | A0A1D6QTZ2, B6T422 | AQK60885, AFW66677 | A0A3L6F9R5 |
| *66* | Zm00001eb240640 | A0A1D6H7L4, B4F810 | AQK70753, AFW71393 | CC746813, CC747041, CC823557, ctg236, pco112909b, rs131187294 |
| *67* | Zm00001eb282660 | A0A1D6LZ70, K7UL98 | AQK84387, AFW77262 | A0A1D6LZ71 |
| *68* | Zm00001eb288550 | B6SZU1 | AQK86131, AFW78082 | B4FVP7 |
| *69* | Zm00001eb295770 | A0A1D6MCJ0, B4FT54 | AQK88434, AFW79152 | - |
| *70* | Zm00001eb339480 | A0A1D6FHB6, B8A1D6 | AQK91224, AFW80519 | A0A1D6FHB7, A0A1D6FHB9 |
| *71* | Zm00001eb340880 | A0A1D6FI63, C0HGU9 | AQK91491, AFW80710 | LOC100381538, NM_001174367, NP_001167838 |
| *72* | Zm00001eb343630 | A0A1D6FJZ7, C0P6C7 | AQK92086, AFW81114 | K7UTD2 |
| *73* | Zm00001eb345630 | A0A1D6FM02, K7UU30 | AQK92697, AFW81379 | A0A1D6FM03, AC199380, AY111286, c0046P07, cl10549-1b, ctg344, rs130794845, rs131179726, rs131681031, rs727141588 |
| *74* | Zm00001eb354940 | A0A1D6FV38, K7VXU7 | AQK95316, AFW82745 | AY109858, BH140794, CC824881, cl82411, ctg354, rs132469999 |
| *75* | Zm00001eb366410 | C0PA53 | AQK99185, AFW84421 | - |
| *76* | Zm00001eb415590 | K7THS8 | AQK41422, AFW57248 | - |
| *77* | Zm00001eb035420 | A0A1D6KJ45, B6TAW2 | ONM02970, DAA48044 | A0A1D6KJ48, A0A1D6KJ50, A0A317YFR2 |
| *78* | Zm00001eb243690 | A0A1D6HAF4, C4J8T7 | AQK71689, AFW71793 | A0A1D6HAF5 |
| *79* | Zm00001eb294570 | A0A1D6MBJ6, B4FTK7 | AQK88137, AFW78974 | A0A096SZL3, A0A1D6MBJ4 |
| *80* | Zm00001eb317080 | A0A1D6I7F3, B4G111 | ONM55991, DAA62093 | A0A1D6I7F2, AC099403, AC203444, AY107498, c0108E24, ctg318, pco134240 |
| *81* | Zm00001eb342590 | A0A1D6FJF4, B4FBV4 | AQK91896, AFW80966 | A0A1D6FJF8, AC200623, AY112585, CC759937, cl2587-2b, ctg333, rs130776147 |
| *82* | Zm00001eb361030 | A0A1D6G2W8, K7W0M3 | AQK97708, AFW83680 | - |
| *83* | Zm00001eb409280 | C0PHP6 | AQK39886, AFW56376 | - |
| *84* | Zm00001eb409300 | A0A1D6IV51, K7TN70 | AQK39887, AFW56377 | - |
| *85* | Zm00001eb409350 | B4FTM2 | AQK39888, AFW56383 | - |
| *86* | Zm00001eb409390 | K7TJI2 | AQK39908, AFW56385 | - |
| *87* | Zm00001eb137150 | B4FR07 | ONM33405, DAA55281 | AC219080, AY107477, CC740187, ctg124, pco093020 |
| *88* | Zm00001eb146070 | A0A1D6N5S3, B4FCM5 | ONM35974, DAA56578 | A0A1D6N5S3 |
| *89* | Zm00001eb162560 | C0P4V7 | ONM41514, DAA59042 | AC189060, AY110279, CC175461, CC744915CC744915, ctg151, EU968804, EZ056856, EZ111789, rs132120891, umc1641 |
| *90* | Zm00001eb205970 | A0A1D6EPZ5, B4FXD1 | AQK60071, AFW66187 | A0A1D6H489, A0A1D6PZA3, A0A1D6QKT2, A0A3L6E2U8, A0A3L6EY14, A0A3L6FPK0, A0A3L6FXZ5, C0PIN6 |
| *91* | Zm00001eb236340 | A0A1D6H489, C0HDZ7 | AQK69639, AFW70759 | A0A1D6H487 |
| *92* | Zm00001eb254160 | A0A1D6HJU8, C0P7T4 | AQK74739, AFW73343 | A0A1D6HJV0, AC203172, AY109911, c0073M20, CC753954, CC829762, cl18199, ctg251, rs131176236 |
| *93* | Zm00001eb279930 | A0A1D6LW01 | AQK83427 | - |
| *94* | Zm00001eb342290 | B4FPY9 | AQK91822, AFW80911 | CC744498, CC759861, cl11333, ctg333, IDP6787 |
| *95* | Zm00001eb346620 | A0A1D6FN25, K7V2H4 | AQK93061, AFW81518 | AC188723, CC826371, AY111847, cl14391-1, ctg337, rs130805330 |
| *96* | Zm00001eb010540 | A0A1D6JVU2, Q6B7Q9 | ONL95914, DAA44367 | A0A317Y2A7 |
| *97* | Zm00001eb046580 | A0A1R3MBV7, Q43298 | ONM06053, DAA49764 | A0A1D6KTZ3, A0A317YBC9, AC027038, AC186905, AI586761, AI739977, AI746162, AI944341, AW202522, AW225294, AY109623, CL2221, CPN60II, CPNB, ctg48, L21006, L21008, mtcpn60II, Q43253, TC170812, Z11546, Z12115 |
| *98* | Zm00001eb065160 | A0A317YBA0, B6SXW8 | ONM11757, DAA52602 | A0A1D6EM50, AC092263, AY108560, B7ZZZ2, ctg67 |
| *99* | Zm00001eb209960 | A0A1D6QV85, K7UGR2 | AQK61253, AFW66838 | A0A3L6EXQ7, B6SH83, B6THN2 |
| *100* | Zm00001eb222300 | A0A804PAZ7, P29185 | AQK65199, AFW68650 | A0A1D6GPU9, AAA33450, AC196086, AFW68651, AY109623, BT016176, BT023997, BT065549, BT087018, CAA77645, cpn60I, csu396, ctg215, EZ083831, EZ092765, L21007, PZA02113, PZA02753, W49907, Z11546, Z12114, ZEAMMB73_664923, ZEAMMB73_202720, ZM05G10560, |
| *101* | Zm00001eb268910 | A0A1D6LJS9, K7UCB5 | AQK79985, AFW75263 | A0A1D6LJS8, AFW75267, ZEAMMB73_095494, ZEAMMB73_244170, ZM06G06970 |
| *102* | Zm00001eb286730 | A0A1X7YIM9, K7US98 | ONM62310, AFW77798 | A0A1X7YIG0, A0A1X7YIH8, AC215201, ACL54466, AY108518, B8A2R7, CPN60-α1, ctg284, hcf133, PCO123570 |
| *103* | Zm00001eb294310 | C0HFM6 | AQK88072, AFW78925 | AZ916313, CPN60-like2, IDP7454, magi78462, rs130445232 |
| *104* | Zm00001eb378930 | A0A1D6NWP6, C0PHP3 | AQL02524, AFW86138 | C0P530 |
| *105* | Zm00001eb001240 | A0A317Y710, C0HGT5 | ONL92943, DAA42956 | T-complex-1-delta |
| *106* | Zm00001eb008400 | A0A1D6JSN1, B6UCD0 | ONL94912, DAA44030 | A0A1D6JSM9, A0A1D6JSN, A0A317Y6T9, AC191457, AY103793, CC756995, ctg9, TC178292 |
| *107* | Zm00001eb111720 | A0A1D6F4L0, B6T8Q5 | ONM26261, DAA41793 | A0A1D6F4L2, A0A1D6N5E0, A0A3L6FWH8, B4FF57, B6U118 |
| *108* | Zm00001eb114320 | A0A1D6F6Y9, C0P2U6 | ONM27003, DAA42171 | A0A1D6F6Y, A0A1D6F6Z, AC183901, AY109440, CC828065, CC831348, CC833557, C0P8C6, ctg108, rs129245414, rs129245430, rs131186200, rs132008067 |
| *109* | Zm00001eb118030 | A0A1D6FAH0, B4FQL5 | ONM28068, DAA42697 | AP003714, AY103729, ctg110, rs131176090 |
| *110* | Zm00001eb127430 | A0A1D6MPG3, G2XK63 | ONM30938, DAA53945 | A0A1D6MPH1, B4F8E0, ONM30947 |
| *111* | Zm00001eb189870 | A0A3L6F3B8, B4F8V9 | AQK55055, AFW63777 | AC068924, AI834628, AI881613, AY104979, ctg182, PCO112869, TC169858 |
| *112* | Zm00001eb192630 | A0A1D6QCF9, B6TKY3 | AQK55906, AFW64184 | A0A1D6QCF7, A0A1D6QCG, A0A3L6F409 |
| *113* | Zm00001eb200400 | A0A3L6F088, K7UCH1 | AQK58420, AFW65297 | AC204880, AY109440, c0173I08, CC786603, ctg194 |
| *114* | Zm00001eb204950 | A0A1D6QQ13, C0PL01 | AQK59624, AFW65994 | A0A1D6QQ15, A0A1D6QQ17, A0A3L6F956 |
| *115* | Zm00001eb207780 | A0A1D6QST1, B4FBP0 | AQK60535, AFW66482 | A0A1D6QSU, A0A3L6F2M8 |
| *116* | Zm00001eb257580 | A0A1D6HN01, B4FMY3 | AQK75688, AFW73829 | A0A1D6HN02, A0A1D6HN04, A0A1D6HN06 |
| *117* | Zm00001eb298950 | A0A1D6HRM1, B4FS49 | ONM51136, DAA59430 | A0A1D6HRM3, A0A1D6HRM5 |
| *118* | Zm00001eb333890 | A0A804Q4W4, K7UPD0 | AQK89675, AFW79714 | A0A1D6FC9, A0A804Q2Z5, A0A804UC92, AY111831, ctg326, TCP-1/cpn60 |
| *119* | Zm00001eb017830 | A0A317YIR8, B6TAA7 | ONL98339, DAA45494 | AY107730, CC740659, ctg14 |
| *120* | Zm00001eb024030 | B4FBN1, A0A3L6DHY3 | ONL99953, DAA46365 | A0A1D6E0F1, A0A317Y1D7, AY110237, B6T3W7, B6T646, B6U478, C4J0L4, CC833770, CC743909, CC754128, ctg20, ctg21 |
| *121* | Zm00001eb071630 | A0A1D6E0F1, B6SN74 | ONM14218, DAA36151 | B6U478 |
| *122* | Zm00001eb110270 | A0A1D6F2C6, B6SLX1 | ONM25586, DAA41590 | - |
| *123* | Zm00001eb290400 | A0A1D6M6G1 | AQK86666, | AY103856, brk3-para, nap1-homo |
| *124* | Zm00001eb329030 | A0A1D6IL03, B4FE30 | ONM60081, DAA63865 | ACG24626, ACG29402, ACG45957, zma:100193174 |
| *125* | Zm00001eb390720 | A0A1D6P6Q3, B4FAE4 | AQL05535, | - |
| *126* | Zm00001eb100100 | A0A1D6EQY9, B6T8U7 | ONM22164, DAA40151 | A0A1D6EQY8, A0A3L6FPW2, AC211512, AY109707, B4FKC1, CC824022, ctg97 |
| *127* | Zm00001eb193040 | A0A1D6QDB3, C0PKD9 | AQK56188, AFW64263 | A0A3L6F6M8, AW191174, BG268094, BT068758, rs129849097, T18415, T20328, umc1610 |
| *128* | Zm00001eb373960 | A0A1D6NSZ2, B4F848 | AQL01350, AFW85447 | K7V9N5 |
| *129* | Zm00001eb012470 | A0A1D6JY70, B6SZ69 | ONL96611, DAA44671 | A0A1D6JY6, A0A317Y6H2, B6U1E4, pco083553, Q9S9I7 |
| *130* | Zm00001eb012510 | A0A1D6JY78 | ONL96620, DAA44676 | A0A1D6JY79, CM007647, XM_008655984, XP_008654206 |
| *131* | Zm00001eb029200 | A0A1D6KE29 | ONM01426, DAA47114 | A0A1D6MWU6, A0A1D6PDJ3, A0A804LQS0, CM007647 |
| *132* | Zm00001eb043470 | A0A317YGW6, B4F8Q2 | - | A0A3L6FMV6, C0P9K2 |
| *133* | Zm00001eb053750 | A0A096R6Z8, B8A3D0 | ONM07984, DAA50790 | A0A1D6L092, A0A317Y7M0, CM007647 |
| *134* | Zm00001eb101990 | C4JBB8 | ONM22625, DAA40410 | AC190523, ACR38468, BM078551, C4JBB8, CC149201, CM007648, ctg98, magi98796, pco093821, TC183541, TIDP3615 |
| *135* | Zm00001eb133480 | A0A1D6MUE8, | ONM32482 | A0A1D6MUF0, GRMZM2G027317 |
| *136* | Zm00001eb136490 | A0A1D6MWV2, B7ZZ42 | ONM33229, DAA55192 | A0A1D6MWU, AC208455, AC210168, BT016812, BT054584, CC452040, ctg124, EU944313, EU959271, X73472, X78414, ZmERD2 |
| *137* | Zm00001eb148420 | A0A1D6N7I4, C4J410 | ONM36552, DAA56909 | A0A1D6N7I2, A0A1D6N7I6, A0A1D6N7I9, CM007649 |
| *138* | Zm00001eb165400 | A0A1D6F848, K7TQM1 | AQK48417, AFW60178 | A0A1D6PNI7, A0A3L6F716 |
| *139* | Zm00001eb209550 | A0A1D6GYL9, B4FWJ8 | AQK61112, AFW66784 | A0A1D6GYM1, A0A1D6GYM2, A0A1D6QUQ8, A0A1D6QUQ9, A0A1D6QUR1, A0A1D6QUR3, A0A3L6ELZ4, A0A3L6F420, AC194475, AC189749, BIP2, cBiPe3, CC750058, CC823317, CC825254, CC832193, ctg202, gsy249a, M59449, ncrb70b, O24581, U58209, umc1058 |
| *140* | Zm00001eb214940 | - | AFW67630 | BIP3 |
| *141* | Zm00001eb217010 | A0A1D6GK64 | AQK63737, AFW67932 | AA231687, AA231688, A0A1D6GK64, BCD1072 |
| *142* | Zm00001eb220140 | A0A1D6GMN4, B4FXX3 | AQK64548, AFW68374 | NM_001158267, NP_001151739, zma:100285374 |
| *143* | Zm00001eb229930 | A0A1D6GYL9, P24067 | AQK67854, AFW69798 | A0A1D6GYM0, A0A1D6GYM1, A0A1D6GYM2, AC208403, AC217639, BIP1, CC464536, ctg223, EU945261, gsy249b, M59449, ncr200b, ncrb70a |
| *144* | Zm00001eb351340 | K7VW90 | AQK94252, AFW82225 | A0A1D6FRN, A0A1D6FRP0, XP_008656365, ZmHsp70-19 |
| *145* | Zm00001eb368000 | C4J410 | AQK99510, AFW84633 | AY914601, BT016462, BT016692, BT085557, BT086887, EU942324, NP_001183165, npi119, P11143, X03658, X03697, X03714, X78415, zma:100501536, ZmHsp70-18 |
| *146* | Zm00001eb397600 | A0A1D6PDJ1, K7VJF3 | AQL07639, AFW88810 | AC083942, AY106152, CC148790, pco094428, XP_008658250, ZmHsp70-21 |
| *147* | Zm00001eb400020 | B7ZXR4 | AQL08622, AFW89181 | ACG29713, B6SXY0, bnl5.09, NP_001148098, pco134267, zma:100281706, ZmHsp70-20 |
| *148* | Zm00001eb409950 | A0A804RDP0, K7UCZ5 | AQK40138, AFW56459 | A0A1D6IW15, AA231687, AA231688, BCD1072, C4J6X8 |
| *149* | Zm00001eb115870 | A0A1D6F848, B4FSP6 | ONM27384, DAA42403 | - |
| *150* | Zm00001eb188700 | A0A1D6Q8C9, K7UYN7 | AQK54662, AFW63584 | A0A1D6Q8C8, AP004161, AY110979, CC149104, ctg182, ZM04G22500 |
| *151* | Zm00001eb252410 | A0A1D6HHX7, B8A0J2 | AQK74125, AFW73067 | A0A1D6HHX7, AC212564, AQK74129, AP004161, AY110979, CC787349, CC788191, cl36581, ctg250, XM_008645274, XP_008643496, ZmBiP3 |
| *152* | Zm00001eb224550 | A0A1D6GSN4 | AQK66021, AFW68969 | A0A1D6GSM, A0A1D6GSN, AQK66027 |
| *153* | Zm00001eb273690 | A0A1D6LPI6, K7UEF9 | AQK81427, AFW75994 | A0A1D6LPI5, A0A1D6LPI6, A0A1D6LPI9, A0A1D6LPJ1, A0A1D6LPJ2, AFW75995, K7VDH9, ZM06G11480 |
| *154* | Zm00001eb283640 | A0A1D6LZY7 | AQK84633, AFW77385 | A0A1D6LZX, A0A1D6LZZ, XP_008647466, ZM06G20290m |
| *155* | Zm00001eb283800 | A0A1D6M007, K7UR48 | AQK84649, AFW77408 | AQK84650, HSP14, XP_008649434, zma:103630125, ZmHsp70-15 |
| *156* | Zm00001eb346940 | A0A1D6FN98 | AQK93148 | A0A1D6FN97, A0A1D6FNA0, A0A1D6FNB3 |
| *157* | Zm00001eb346960 | A0A1D6FNB8, B6U237 | AQK93152, AFW81562 | A0A1D6FNC, AI770497, AP003047, B7ZZJ4, ctg340, umc1415 |
| *158* | Zm00001eb101750 | A0A3L6FZE4 | ONM22533, DAA40366 | A0A1D6ES19, A0A1D6ES21, pza03529, rs131971025, ss196415213 |
| *159* | Zm00001eb137900 | A0A1D6MXW5, C0PDG3 | ONM33566, DAA55368 | - |
| *160* | Zm00001eb199230 | A0A1D6QK73, K7V364 | AQK58130, AFW65129 | A0A1D6QK75, A0A3L6F9G2, C0PJ29, IDP7330, rs132182898 |
| *161* | Zm00001eb199590 | A0A1D6QKI4, C3UZ63 | AQK58233, AFW65179 | A0A1D6KID1, A0A3L6F8S8, C0PLZ0, FJ805746 |
| *162* | Zm00001eb228120 | A0A1D6GWE1, B8A0P3 | AQK67196, AFW69515 | 0A1D6GWD9, AC155503, AC209334, AY103537, c0032N18, CC757715, CC805967, ctg220, EU947132, EU950875, EZ081463, EZ096484, EZ107707, EZ114144, IDP7018, magi32079, PCO098403, shpl1, TC169170 |
| *163* | Zm00001eb264470 | A0A1D6LFA6, K7V9P8 | AQK78627, AFW74645 | A0A1D6LFA5, A0A1D6LFA7, A0A1D6LFB0, AQK78628 |
| *164* | Zm00001eb272290 | A0A1D6LMW9 | AQK80922, AFW75767 | A0A1D6LMX0, AC206514, c0142I08, ctg270, rs131179309, rs131577814, shpl2 |
| *165* | Zm00001eb315880 | A0A1D6I6H5, B8A158 | ONM55676, DAA61912 | - |
| *166* | Zm00001eb316410 | A0A1D6I6T0 | ONM55777, DAA61991 | A0A1D6I6S9, A0A1D6I6T, ONM55783, pco150589 |
| *167* | Zm00001eb418610 | C0P4Q3 | AQK42298, AFW57714 | ACN27969, HSP82, NP_001135416, Q08277, S59780, zma:100196928 |
| *168* | Zm00001eb034130 | A0A1D6KIC6, C4J3K8 | ONM02740, DAA47844 | A0A1D6QKI7, A0A1D6KID1, IDP6902, pco153543, pza03531, rs128285185, rs131175311, ss196414630, TC168775 |
| *169* | Zm00001eb020330 | A0A1D6K5Y7, C0HG17 | ONL98985, DAA45851 | A0A317Y9T0, GRMZM5G833665 |
| *170* | Zm00001eb084420 | A0A3L6D9M7, C0PFZ0 | ONM17820, DAA37986 | A0A1D6FFG1, A0A1D6LIV8, A0A1D6MHQ2, A0A1D6Q3K7, A0A3L6DQ89, A0A3L6EIB2, A0A3L6FDA5, A0A3L6FVA2, AY108784, ctg80, pco133857, rs129076096, rs129076101, rs131349392 |
| *171* | Zm00001eb084890 | A0A1D6EC46, C4J9D9 | ONM17897, DAA38058 | A0A1D6EC43, A0A1D6EC44, A0A1D6EC51 |
| *172* | Zm00001eb234160 | A0A1D6H2L6, B4FL89 | AQK69080, AFW70447 | AA231821, rs130070462, rs131543677, rz87-clp |
| *173* | Zm00001eb242420 | A0A1D6H9K3, C0PI40 | AQK71386, AFW71636 | ZmERD1 |
| *174* | Zm00001eb293780 | A0A1D6MAR6, C0PDC7 | AQK87867, AFW78844 | ACN32172, AF083327, AF133840, AY485529, CL1518, BT018333, EU943411, EU943494, ms42, NM_001111465, NP_001104935, rs130443284, zma:541780 |
| *175* | Zm00001eb393250 | K7VD78 | AQL06127, AFW88139 | AC155586, AC190857, AY105111, ctg385, pco093519b, rs131127311 |
| *176* | Zm00001eb411490 | A0A1D6IXA0, C0PFV4 | AQK40546, AFW56690 | A0A1D6IXA1, AC205627, AC211866, AY108805, c0151G24, CC145312, ctg396, rs128309149, T14785, T18738, uaz242-clp |
| *177* | Zm00001eb421360 | A0A1D6J612, K7TSX2 | AQK43358, AFW58112 | A0A1D6J609, A0A1D6J610, A0A1D6J631 |
| *178* | Zm00001eb086360 | A0A1D6EDF1 | ONM18317, DAA38303 | A0A1D6EDE9, A0A1D6EDF0, A0A3L6G3R6 |
| *179* | Zm00001eb178400 | A0A3L6F5G3, K7UUD1 | AQK52148, AFW62064 | AY111260, CC823684, cl8508-1, ctg172, PZA02767, rs131175599, rs55625501, ss196415768 |
| *180* | Zm00001eb197530 | A0A3L6F386, K7U3L9 | AQK57485, AFW64883 | umc15a |
| *181* | Zm00001eb412800 | K7TGQ6 | AQK40746, AFW56848 | - |
| *182* | Zm00001eb420410 | A0A1D6J560, K7UHH8 | AQK43074, AFW57964 | A0A1D6J562, A0A1D6J563 |

**Table S6** List of the orthologous genes to those coding for the 182 maize HSPs, identified in the sorghum and rice genomes:

| **#** | **Zm-B73-NAM-5.0** | ***Sorghum bicolor*** | ***Oryza sativa*** |
| --- | --- | --- | --- |
| *1* | Zm00001eb011880 | SORBI_3001G426000, Sb01g040030 | BGIOSGA012297, LOC_Os03g15960 |
| *2* | Zm00001eb011900 | SORBI_3001G426000, Sb01g040030 | BGIOSGA012297, LOC_Os03g15960 |
| *3* | Zm00001eb011930 | Sb01g039990 | LOC_Os03g16020, LOC_Os03g16040 |
| *4* | Zm00001eb124940 | SORBI_3003G082000, SORBI_3003G081900, Sb03g006870 | LOC_Os01g04370, LOC_Os01g04380 |
| *5* | Zm00001eb124960 | Sb03g006880 | LOC_Os01g04370, LOC_Os01g04380 |
| *6* | Zm00001eb335430 | Sb03g006880 | LOC_Os01g04370, LOC_Os01g04380 |
| *7* | Zm00001eb397940 | SORBI_3001G425600, Sb01g040000 | BGIOSGA012297, LOC_Os03g15960, LOC_Os03g16030 |
| *8* | Zm00001eb121900 | SORBI_3003G039400, Sb03g003530 | BGIOSGA002242, LOC_Os01g08860, Os01g0184100 |
| *9* | Zm00001eb257130 | SORBI_3004G321000, Sb04g035130 | BGIOSGA009154, LOC_Os02g54140, Os02G0782500 |
| *10* | Zm00001eb311990 | SORBI_3004G321000 | BGIOSGA009154, LOC_Os02g54140, Os02g0782500 |
| *11* | Zm00001eb337690 | SORBI_3003G039400, Sb03g003530 | BGIOSGA002242, LOC_Os01g08860, Os01g0184100 |
| *12* | Zm00001eb395330 | SORBI_3003G039400 | BGIOSGA002242, LOC_Os01g08860, Os01g0184100 |
| *13* | Zm00001eb395360 | SORBI_3003G039400 | BGIOSGA002242, LOC_Os01g08860, Os01g0184100 |
| *14* | Zm00001eb395370 | SORBI_3003G039400 | LOC_Os01g01841, LOC_Os01g08860, Os01g0184100 |
| *15* | Zm00001eb081770 | SORBI_3006G093500, Sb06g017850 | BGIOSGA016481, LOC_Os04g36750, Os04g0445100 |
| *16* | Zm00001eb112330 | SORBI_3005G086400, Sb05g007030 | LOC_Os11g13980 |
| *17* | Zm00001eb125010 | SORBI_3003G082300, Sb03g006910 | BGIOSGA002722, LOC_Os01g04350 |
| *18* | Zm00001eb125030 | SORBI_3003G082500, Sb03g006920 | BGIOSGA002721, LOC_Os01g04340 |
| *19* | Zm00001eb208810 | SORBI_3004G026300, Sb04g002330 | BGIOSGA007446, LOC_Os02g03570 |
| *20* | Zm00001eb252460 | SORBI_3004G263100, Sb04g030135 | BGIOSGA008910, LOC_Os02g48140 |
| *21* | Zm00001eb291920 | SORBI_3009G187400, Sb09g024680 | BGIOSGA020122, LOC_Os05g42120, Os05g0500500 |
| *22* | Zm00001eb352720 | SORBI_3009G187400, Sb09g024680 | BGIOSGA020122, LOC_Os05g42120 |
| *23* | Zm00001eb371530 | SORBI_3010G104300, Sb10g009090 | BGIOSGA021524, LOC_Os06g14240, Os06g0253100 |
| *24* | Zm00001eb423300 | SORBI_3006G093500, Sb06g017850 | BGIOSGA016481, LOC_Os04g36750 |
| *25* | Zm00001eb010530 | SORBI_3001G438000, Sb01g041180 | BGIOSGA012224, LOC_Os03g14180, Os03g0245800, Os03g0835700 |
| *26* | Zm00001eb026540 | SORBI_3001G265800, Sb01g025960 | BGIOSGA030564, LOC_Os10g07210 |
| *27* | Zm00001eb026680 | SORBI_3001G264400, Sb01g025610 | BGIOSGA032267, LOC_Os10g07200 |
| *28* | Zm00001eb026690 | SORBI_3001G264300, Sb01g025600 | BGIOSGA032265, LOC_Os10g07210 |
| *29* | Zm00001eb032310 | SORBI_3008G178901, Sb08g022210 | LOC_Os12g42876, LOC_Os12g42884, Os03g0656100, Os12g0624100 |
| *30* | Zm00001eb034050 | SORBI_3007G217300, Sb07g028370 | BGIOSGA018362, LOC_Os03g14180 |
| *31* | Zm00001eb047490 | SORBI_3001G239400, Sb01g021180 | BGIOSGA033012, LOC_Os10g30162 |
| *32* | Zm00001eb052730 | SORBI_3001G148600, Sb01g012930 | BGIOSGA010121, LOC_Os03g45340 |
| *33* | Zm00001eb124950 | Sb03g006880 | LOC_Os01g04370, LOC_Os01g04380 |
| *34* | Zm00001eb193940 | SORBI_3004G228900, Sb04g027330 | BGIOSGA009084, LOC_Os02g52150, Os02g0758000 |
| *35* | Zm00001eb218280 | SORBI_3001G148600, Sb01g012930 | BGIOSGA010121, LOC_Os03g45340 |
| *36* | Zm00001eb218310 | SORBI_3001G148800, Sb01g012950 | BGIOSGA010122, LOC_Os03g45330 |
| *37* | Zm00001eb222770 | SORBI_3001G239200, Sb01g021170 | BGIOSGA033013, LOC_Os10g30180 |
| *38* | Zm00001eb222820 | SORBI_3001G239400, Sb01g021180 | BGIOSGA033012, LOC_Os10g30162, Os10g0437700 |
| *39* | Zm00001eb235800 | SORBI_3004G083600, Sb04g006890 | BGIOSGA006925, LOC_Os02g10710 |
| *40* | Zm00001eb297350 | SORBI_3009G255200, Sb09g030540, Sb09g030550 | LOC_Os01g40550, LOC_Os05g51440, LOC_Os05g51450 |
| *41* | Zm00001eb322740 | SORBI_3002G319100, Sb02g034760 | BGIOSGA025811, LOC_Os07g33350, Os07g0517100 |
| *42* | Zm00001eb372830 | SORBI_3010G087600, Sb10g007600 | BGIOSGA021653, LOC_Os06g11610 |
| *43* | Zm00001eb050280 | SORBI_3001G154100, Sb01g013390 | BGIOSGA014816, LOC_Os03g44620, Os03g0648400 |
| *44* | Zm00001eb060050 | SORBI_3001G064500, Sb01g005860 | BGIOSGA013692, LOC_Os03g57340, Os03g0787300 |
| *45* | Zm00001eb091890 | SORBI_3001G064500 | BGIOSGA013692, LOC_Os03g57340, Os03g0787300 |
| *46* | Zm00001eb103670 | SORBI_3001G064500 | BGIOSGA013692, LOC_Os03g57340, OS03G0787300 |
| *47* | Zm00001eb107580 | SORBI_3001G064500 | BGIOSGA013692, LOC_Os03g57340, Os03g0787300 |
| *48* | Zm00001eb172900 | SORBI_3007G005400 | BGIOSGA018283, LOC_Os05g26926 |
| *49* | Zm00001eb192080 | SORBI_3004G336000, Sb04g036400 | BGIOSGA005412, LOC_Os02g56040, Os02g0804500 |
| *50* | Zm00001eb213310 | SORBI_3001G064500, Sb01g005860 | BGIOSGA013692, LOC_Os03g57340, Os03g0787300 |
| *51* | Zm00001eb218560 | SORBI_3001G154100, Sb01g013390 | BGIOSGA014816, LOC_Os03g44620, Os03g0648400 |
| *52* | Zm00001eb249430 | SORBI_3004G295800, Sb04g032970 | BGIOSGA008745, LOC_Os02g43930 |
| *53* | Zm00001eb258200 | SORBI_3004G336000, Sb04g036400, Sb04g036413 | BGIOSGA005412, LOC_Os02g56040, LOC_Os03g29810, Os02g0804500 |
| *54* | Zm00001eb269190 | SORBI_3010G014100, Sb10g001410 | BGIOSGA022052, LOC_Os06g02620, Os06g0116800 |
| *55* | Zm00001eb294320 | SORBI_3007G005400 | BGIOSGA018283 |
| *56* | Zm00001eb379790 | SORBI_3001G064500, Sb10g004410 | BGIOSGA013692, LOC_Os03g57340, LOC_Os06g06770, Os03g0787300 |
| *57* | Zm00001eb392350 | SORBI_3007G005400 | BGIOSGA018283, LOC_Os05g26926 |
| *58* | Zm00001eb404330 | SORBI_3001G064500 | BGIOSGA013692, LOC_Os03g57340, Os03g0787300 |
| *59* | Zm00001eb407790 | SORBI_3008G051200, Sb08g004320 | BGIOSGA037058, LOC_Os12g07060, Os12g0168400 |
| *60* | Zm00001eb416450 | SORBI_3007G005400, | BGIOSGA018283, Os05g0334000, Os05g0333500, Os05g0334400 |
| *61* | Zm00001eb429820 | SORBI_3001G064500 | BGIOSGA013692, Os03g0787300 |
| *62* | Zm00001eb009060 | SORBI_3001G452000, Sb01g042488 | BGIOSGA012143, LOC_Os03g12236 |
| *63* | Zm00001eb042680 | SORBI_3007G104600, Sb07g014620 | BGIOSGA027061, LOC_Os08g28700, Os08g0374400 |
| *64* | Zm00001eb118880 | SORBI_3003G002400, Sb03g000380 | BGIOSGA003104, LOC_Os01g13760, Os01g0239100 |
| *65* | Zm00001eb208880 | SORBI_3004G027200, Sb04g002410 | BGIOSGA025789, LOC_Os02g03600 |
| *66* | Zm00001eb240640 | - | BGIOSGA008036, LOC_Os02g20394 |
| *67* | Zm00001eb282660 | SORBI_3009G051700, Sb09g004380 | BGIOSGA019215, LOC_Os05g06440, Os05g0156500 |
| *68* | Zm00001eb288550 | SORBI_3009G146400, Sb09g021110 | BGIOSGA018015, Os05g0427900 |
| *69* | Zm00001eb295770 | SORBI_3009G231400, Sb09g028410 | BGIOSGA017566, LOC_Os05g48810 |
| *70* | Zm00001eb339480 | SORBI_3003G002400, Sb03g000380 | BGIOSGA003104, LOC_Os01g13760, Os01g0239100 |
| *71* | Zm00001eb340880 | SORBI_3003G155800, Sb03g013590 | LOC_Os05g06440 |
| *72* | Zm00001eb343630 | SORBI_3009G231400, Sb09g028410 | BGIOSGA017566, LOC_Os05g48810 |
| *73* | Zm00001eb345630 | SORBI_3009G051700 | BGIOSGA019215, LOC_Os05g06440, Os05g0156500 |
| *74* | Zm00001eb354940 | SORBI_3009G028100, Sb09g002340 | BGIOSGA019109, LOC_Os05g03630 |
| *75* | Zm00001eb366410 | SORBI_3003G374200, Sb03g041460 | BGIOSGA004908, LOC_Os01g65480, Os01g0875700 |
| *76* | Zm00001eb415590 | SORBI_3007G051300, Sb07g004160 | BGIOSGA027605, LOC_Os08g06460 |
| *77* | Zm00001eb035420 | SORBI_3007G205800, Sb07g027320 | BGIOSGA029020, LOC_Os08g41110, Os08g0522600 |
| *78* | Zm00001eb243690 | SORBI_3004G176800, Sb04g022680 | BGIOSGA006262, LOC_Os02g35000, Os02g0555700 |
| *79* | Zm00001eb294570 | SORBI_3009G217000, Sb09g027210 | LOC_Os05g46620 |
| *80* | Zm00001eb317080 | SORBI_3002G251900, Sb02g028760 | BGIOSGA029517, LOC_Os09g32050, Os09g0493800 |
| *81* | Zm00001eb342590 | SORBI_3009G217000, Sb09g027210 | LOC_Os05g46620 |
| *82* | Zm00001eb361030 | SORBI_3003G270100, Sb03g032250 | BGIOSGA004279, LOC_Os01g50700, Os01g0702450 |
| *83* | Zm00001eb409280 | SORBI_3008G104400, Sb08g015620 | BGIOSGA037443, LOC_Os12g31840, Os12g0502700 |
| *84* | Zm00001eb409300 | SORBI_3008G104400, Sb08g015620 | BGIOSGA037443, LOC_Os12g31840, Os12g0502700 |
| *85* | Zm00001eb409350 | SORBI_3008G104400, Sb08g015620 | BGIOSGA037443, LOC_Os12g31840, Os12g0502700 |
| *86* | Zm00001eb409390 | SORBI_3008G104400, Sb08g015620 | BGIOSGA037443, LOC_Os12g31840, Os12g0502700 |
| *87* | Zm00001eb137150 | SORBI_3008G124900, Sb08g017710 | BGIOSGA003624, LOC_Os01g32870, Os01g0512100 |
| *88* | Zm00001eb146070 | SORBI_3003G374200, Sb03g041460 | LOC_Os01g65480, Os01g0875700 |
| *89* | Zm00001eb162560 | SORBI_3003G185200, Sb03g024860 | BGIOSGA003752, Os01g0556400 |
| *90* | Zm00001eb205970 | SORBI_3004G079200, Sb04g006540 | BGIOSGA007728, Os02g0195800 |
| *91* | Zm00001eb236340 | SORBI_3004G079200, Sb04g006540 | BGIOSGA007728, LOC_Os02g10220, Os02g0195800 |
| *92* | Zm00001eb254160 | SORBI_3004G242700, Sb04g028350 | BGIOSGA009026, LOC_Os02g50760, Os02g0741100 |
| *93* | Zm00001eb279930 | SORBI_3010G098600 | BGIOSGA021571 |
| *94* | Zm00001eb342290 | SORBI_3003G185200, Sb03g024860 | BGIOSGA003752, LOC_Os01g37560, Os01g0556400 |
| *95* | Zm00001eb346620 | SORBI_3009G113000, Sb09g018100 | BGIOSGA018199, LOC_Os05g30130, Os05g0364500 |
| *96* | Zm00001eb010540 | SORBI_3001G437801, Sb01g041170 | BGIOSGA034997, Os04g0443900 |
| *97* | Zm00001eb046580 | Sb01g020010 | BGIOSGA033085, LOC_Os10g32550 |
| *98* | Zm00001eb065160 | SORBI_3001G003100, Sb01g000380 | BGIOSGA009394, LOC_Os03g64210, Os03g0859600, Os03g0859700 |
| *99* | Zm00001eb209960 | SORBI_3004G002200, Sb04g000370 | BGIOSGA007332, LOC_Os02g01280, Os02g0102900 |
| *100* | Zm00001eb222300 | SORBI_3001G228200, Sb01g020010 | BGIOSGA033085, LOC_Os10g32550, Os10g0462900, Os10g0462900 |
| *101* | Zm00001eb268910 | SORBI_3010G011200, Sb10g001120 | BGIOSGA022066, LOC_Os06g02380, Os06g0114000 |
| *102* | Zm00001eb286730 | SORBI_3009G098100, Sb09g014430 | BGIOSGA037230, LOC_Os12g17910, Os12g0277500 |
| *103* | Zm00001eb294310 | SORBI_3009G214100, Sb09g026970 | BGIOSGA017649, LOC_Os05g46290, Os05g0540300 |
| *104* | Zm00001eb378930 | SORBI_3010G011200, Sb10g001120 | BGIOSGA022066, LOC_Os06g02380, Os06g0114000 |
| *105* | Zm00001eb001240 | SORBI_3001G530800, Sb01g049370 | BGIOSGA028596, LOC_Os02g22780, Os02g0332200, Os10g0514600 |
| *106* | Zm00001eb008400 | SORBI_3001G460500, Sb01g043220 | BGIOSGA024073, LOC_Os06g34690, Os06g0538000 |
| *107* | Zm00001eb111720 | SORBI_3002G423500, Sb02g043440 | BGIOSGA018808, Os05g0147400 |
| *108* | Zm00001eb114320 | SORBI_3005G163700, Sb05g022470 | BGIOSGA013764, LOC_Os11g36950, Os03g0804800 |
| *109* | Zm00001eb118030 | SORBI_3002G014400, Sb02g001450 | BGIOSGA021032, Os06g0562600 |
| *110* | Zm00001eb127430 | SORBI_3003G060500, SORBI_3003G112800 | BGIOSGA013131, LOC_Os03g42220 |
| *111* | Zm00001eb189870 | SORBI_3001G530800 | BGIOSGA028596, LOC_Os02g22780, Os10g0514600 Os02g0332200 |
| *112* | Zm00001eb192630 | SORBI_3004G326600, Sb04g035610 | BGIOSGA016889, LOC_Os04g46620, Os04g0551800 |
| *113* | Zm00001eb200400 | SORBI_3005G163700, Sb05g022470 | BGIOSGA013764, LOC_Os03g59020, LOC_Os11g36950, Os03g0804800 |
| *114* | Zm00001eb204950 | SORBI_3001G460500 | BGIOSGA024073, LOC_Os02g14929, LOC_Os06g34690, Os06g0538000 |
| *115* | Zm00001eb207780 | SORBI_3004G047200, Sb04g004030 | BGIOSGA020645, LOC_Os02g06350, LOC_Os06g47320, Os06g0687700 |
| *116* | Zm00001eb257580 | SORBI_3004G326600, Sb04g035610 | BGIOSGA016889, LOC_Os04g46620, Os04g0551800 |
| *117* | Zm00001eb298950 | SORBI_3002G014400, Sb02g001450 | BGIOSGA021032, LOC_Os06g36700, Os06g0562600 |
| *118* | Zm00001eb333890 | SORBI_3003G060500, SORBI_3003G112800, Sb03g009490 | BGIOSGA013131 |
| *119* | Zm00001eb017830 | SORBI_3001G361600, Sb01g034530 | BGIOSGA010676, LOC_Os03g25050 |
| *120* | Zm00001eb024030 | SORBI_3001G293300, Sb01g028650 | BGIOSGA033465, LOC_Os10g41710, Os10g0566700 |
| *121* | Zm00001eb071630 | SORBI_3001G293300 | BGIOSGA033465, Os10g0566700 |
| *122* | Zm00001eb110270 | SORBI_3002G393400, Sb02g040870 | BGIOSGA026240, LOC_Os07g44740, Os07g0641700 |
| *123* | Zm00001eb290400 | SORBI_3002G393400 | LOC_Os08g43130, Os08g0544500 |
| *124* | Zm00001eb329030 | SORBI_3002G393400, Sb02g040870 | BGIOSGA026240, LOC_Os07g44740, Os07g0641700 |
| *125* | Zm00001eb390720 | SORBI_3001G293300 | BGIOSGA033465, Os10g0566700 |
| *126* | Zm00001eb100100 | SORBI_3002G214600, Sb02g025710 | BGIOSGA029691, LOC_Os09g26760, Os09g0439000 |
| *127* | Zm00001eb193040 | SORBI_3004G319800, Sb04g035040 | BGIOSGA005485, LOC_Os02g54060, LOC_Os06g09679, Os02g0781400 |
| *128* | Zm00001eb373960 | SORBI_3010G073700, Sb10g006450 | BGIOSGA022469, LOC_Os06g09679, Os06g0196900, Os06g0197575 |
| *129* | Zm00001eb012470 | SORBI_3001G420100, Sb01g039530 | BGIOSGA012333, LOC_Os03g16860, Os03g0276500 |
| *130* | Zm00001eb012510 | SORBI_3001G419100, Sb01g039440 | BGIOSGA034961, LOC_Os03g16880, LOC_Os11g08460 |
| *131* | Zm00001eb029200 | SORBI_3008G136000, Sb08g018750 | BGIOSGA035736, LOC_Os12g38180, LOC_Os11g47760, Os11g0703900 |
| *132* | Zm00001eb043470 | SORBI_3001G193500 | BGIOSGA011582 |
| *133* | Zm00001eb053750 | SORBI_3001G129000, Sb01g011310 | BGIOSGA019575, LOC_Os05g23740 |
| *134* | Zm00001eb101990 | SORBI_3002G249800, Sb02g028570 | BGIOSGA029532, LOC_Os09g31486 |
| *135* | Zm00001eb133480 | SORBI_3008G088200 | BGIOSGA037214, LOC_Os12g14070, Os12g0244100 |
| *136* | Zm00001eb136490 | SORBI_3008G136000, Sb08g018750 | BGIOSGA035736, LOC_Os12g38180, LOC_Os11g47760, Os11g0703900 |
| *137* | Zm00001eb148420 | SORBI_3003G350700, Sb03g039360 | LOC_Os01g62290, LOC_Os05g38530 |
| *138* | Zm00001eb165400 | - | BGIOSGA010330 |
| *139* | Zm00001eb209550 | SORBI_3004G011700, Sb04g001140 | BGIOSGA007251, LOC_Os02g02410, Os02g0115900 |
| *140* | Zm00001eb214940 | SORBI_3001G118600 | BGIOSGA013428, LOC_Os03g50250 |
| *141* | Zm00001eb217010 | SORBI_3001G129000, Sb01g011310 | BGIOSGA019575, LOC_Os05g23740 |
| *142* | Zm00001eb220140 | SORBI_3001G193500, Sb01g017050 | BGIOSGA011582, LOC_Os02g53420, Os03g0113700, Os02g0774300 |
| *143* | Zm00001eb229930 | SORBI_3004G011700, Sb04g001140 | BGIOSGA007251, LOC_Os02g02410, Os02g0115900 |
| *144* | Zm00001eb351340 | SORBI_3009G163900, Sb09g022580 | LOC_Os05g38530, Os05g0460000 |
| *145* | Zm00001eb368000 | SORBI_3003G350700, Sb03g039360 | LOC_Os01g62290, LOC_Os05g38530 |
| *146* | Zm00001eb397600 | SORBI_3001G418600, Sb01g039390 | BGIOSGA011002, LOC_Os03g16920 |
| *147* | Zm00001eb400020 | SORBI_3001G454000, Sb01g042680 | BGIOSGA011204, LOC_Os03g11910, Os03g0218500 |
| *148* | Zm00001eb409950 | SORBI_3008G088200 | BGIOSGA037214, LOC_Os12g14070, Os12g0244100 |
| *149* | Zm00001eb115870 | - | BGIOSGA010330 |
| *150* | Zm00001eb188700 | SORBI_3004G263500, Sb04g030160 | BGIOSGA008908, LOC_Os02g48110, Os02g0710900 |
| *151* | Zm00001eb252410 | SORBI_3004G263500, Sb04g030160 | BGIOSGA008908, LOC_Os02g48110, Os02g0710900 |
| *152* | Zm00001eb224550 | SORBI_3010G230600, Sb10g027320 | BGIOSGA023469, LOC_Os06g46600, Os06g0679800 |
| *153* | Zm00001eb273690 | SORBI_3010G230600, Sb10g027320 | BGIOSGA023469, LOC_Os06g46600, Os06g0679800 |
| *154* | Zm00001eb283640 | SORBI_3009G067000, Sb09g005580 | LOC_Os01g08560 |
| *155* | Zm00001eb283800 | SORBI_3009G066900, Sb09g005570 | LOC_Os01g08560 |
| *156* | Zm00001eb346940 | SORBI_3009G067000 | - |
| *157* | Zm00001eb346960 | SORBI_3009G066900 | LOC_Os05g08840, LOC_Os01g08560 |
| *158* | Zm00001eb101750 | SORBI_3002G243500, Sb02g028050 | LOC_Os09g30412 |
| *159* | Zm00001eb137900 | SORBI_3008G111600, Sb08g016560 | BGIOSGA036094, LOC_Os12g32986, Os12g0514500 |
| *160* | Zm00001eb199230 | SORBI_3007G224100, Sb07g028940 | BGIOSGA026764, LOC_Os08g38086, Os08g0487800 |
| *161* | Zm00001eb199590 | SORBI_3007G216300, Sb07g028270 | BGIOSGA031149, LOC_Os08g39140, LOC_Os09g30412 |
| *162* | Zm00001eb228120 | Sb10g030240 | BGIOSGA020527, LOC_Os06g50300 |
| *163* | Zm00001eb264470 | SORBI_3008G111600 | BGIOSGA036094, LOC_Os12g32986, Os12g0514500 |
| *164* | Zm00001eb272290 | SORBI_3010G267400, Sb10g030240 | BGIOSGA020527, LOC_Os06g50300, Os06g0716700 |
| *165* | Zm00001eb315880 | - | BGIOSGA029594, LOC_Os09g29840 |
| *166* | Zm00001eb316410 | Sb02g028050 | LOC_Os09g30412, LOC_Os08g39140 |
| *167* | Zm00001eb418610 | SORBI_3006G005600, Sb06g000660 | BGIOSGA015767, LOC_Os04g01740, Os04g0445100 |
| *168* | Zm00001eb034130 | Sb07g028270 | LOC_Os08g39140 |
| *169* | Zm00001eb020330 | SORBI_3001G333500, Sb01g032210 | BGIOSGA012917, Os03g0426900 |
| *170* | Zm00001eb084420 | SORBI_3006G065100, Sb06g015220 | BGIOSGA016335, LOC_Os04g33210 |
| *171* | Zm00001eb084890 | SORBI_3006G058100, Sb06g014590 | BGIOSGA015181, LOC_Os04g32560, Os04g0397100 |
| *172* | Zm00001eb234160 | SORBI_3004G066500, Sb04g005570 | BGIOSGA007016, LOC_Os02g08490, Os02g0181900 |
| *173* | Zm00001eb242420 | SORBI_3004G162400, Sb04g021410 | BGIOSGA008338, LOC_Os02g32520, Os02g0526400 |
| *174* | Zm00001eb293780 | SORBI_3009G201500, Sb09g025900 | BGIOSGA017714, LOC_Os05g44340 |
| *175* | Zm00001eb393250 | SORBI_3001G333500, Sb01g032210 | BGIOSGA012917, LOC_Os03g31300, Os03g0426900 |
| *176* | Zm00001eb411490 | SORBI_3008G081900, Sb08g007750 | BGIOSGA036427, LOC_Os12g12850 |
| *177* | Zm00001eb421360 | Sb06g014590 | BGIOSGA015181, LOC_Os04g32560 |
| *178* | Zm00001eb086360 | - | BGIOSGA015394, LOC_Os04g23220 |
| *179* | Zm00001eb178400 | Sb07g008090 | BGIOSGA027355, LOC_Os08g15230 |
| *180* | Zm00001eb197530 | SORBI_3005G043000, Sb05g003570 | BGIOSGA034838, LOC_Os11g05820, Os11g0156800 |
| *181* | Zm00001eb412800 | SORBI_3007G090900, Sb07g008090 | BGIOSGA027355, LOC_Os08g15230, Os08g0250900 |
| *182* | Zm00001eb420410 | - | BGIOSGA015394, LOC_Os04g23220 |

**Table S7** List of the orthologous genes to those coding for the 182 maize HSPs, identified in the millet, brachypodium and arabidopsis genomes:

| **#** | **Zm-B73-NAM-5.0** | ***Setaria italica*** | ***Brachypodium distachyon*** | ***Arabidopsis thaliana*** |
| --- | --- | --- | --- | --- |
| *1* | Zm00001eb011880 | SETIT_038751mg, Si038751m.g | BRADI_1g67080v3 | AT1G53540 |
| *2* | Zm00001eb011900 | SETIT_038751mg, Si038751m.g | BRADI_1g67080v3 | AT1G53540 |
| *3* | Zm00001eb011930 | Si037867m.g | BRADI_1g67040v3 | AT1G53540 |
| *4* | Zm00001eb124940 | SETIT_002731mg | BRADI_2g02410v3 | AT1G53540 |
| *5* | Zm00001eb124960 | Si002731m.g | BRADI_2g02410v3 | AT1G53540 |
| *6* | Zm00001eb335430 | Si002731m.g | BRADI_2g02410v3 | AT1G53540 |
| *7* | Zm00001eb397940 | SETIT_037867mg, Si037867m.g | BRADI_1g67080v3 | AT1G53540 |
| *8* | Zm00001eb121900 | SETIT_004066mg, Si004066m.g, Si005062m.g | BRADI_2g05374v3, BRADI2G05380 | AT5G12020, AT5G12030 |
| *9* | Zm00001eb257130 | SETIT_018495mg, Si018495m.g | BRADI_3g60100v3 | AT1G54050 |
| *10* | Zm00001eb311990 | SETIT_018495mg, Si018495m.g | BRADI_3g60100v3 | AT1G54050 |
| *11* | Zm00001eb337690 | SETIT_004066mg, Si005062m.g, Si004066m.g | BRADI_2g05374v3 | AT5G12030 |
| *12* | Zm00001eb395330 | SETIT_004066mg, Si004066m.g, Si005062m.g | BRADI_2g05374v3 | AT5G12020, AT5G12030 |
| *13* | Zm00001eb395360 | SETIT_004066mg, Si005062m.g, Si004066m.g | BRADI_2G05380v3, BRADI_2g05374v3 | AT5G12020 |
| *14* | Zm00001eb395370 | SETIT_004066mg, Si005062m.g, Si004066m.g | BRADI_2g05374v3 | AT5G12020 |
| *15* | Zm00001eb081770 | SETIT_011036mg, Si011036m.g | BRADI_5g11110v3 | AT4G10250 |
| *16* | Zm00001eb112330 | SETIT_026764mg, Si026764m.g | BRADI_4g21070v3 | AT4G10250 |
| *17* | Zm00001eb125010 | SETIT_003160mg, Si003160m.g | BRADI_2g02350v3 | AT1G07400 |
| *18* | Zm00001eb125030 | SETIT_003245mg, Si003245m.g | BRADI_2g02400v3 | AT1G53540 |
| *19* | Zm00001eb208810 | SETIT_018487mg, Si018487m.g | BRADI_3g02710v3 | AT1G53540 |
| *20* | Zm00001eb252460 | SETIT_018542mg, Si018542m.g | - | AT1G53540 |
| *21* | Zm00001eb291920 | SETIT_023317mg, Si023317m.g | BRADI_2g20767v3 | AT5G54660 |
| *22* | Zm00001eb352720 | SETIT_023317mg, Si023317m.g | BRADI_2g20767v3 | AT5G54660 |
| *23* | Zm00001eb371530 | SETIT_007440mg, Si007440m.g | BRADI_1g44230v3 | AT5G37670 |
| *24* | Zm00001eb423300 | SETIT_011036mg, Si011036m.g | BRADI_5g11110v3 | AT4G10250 |
| *25* | Zm00001eb010530 | SETIT_037240mg, Si037240m.g | BRADI_1g68440v3 | AT4G27670 |
| *26* | Zm00001eb026540 | SETIT_037723mg, Si037723m.g | - | - |
| *27* | Zm00001eb026680 | SETIT_037486mg, Si037486m.g | BRADI_3g20840v3 | AT4G27670 |
| *28* | Zm00001eb026690 | SETIT_037306mg, Si037306m.g | BRADI_3g20830v3 | AT4G27670 |
| *29* | Zm00001eb032310 | SETIT_024825mg, Si024825m.g | BRADI_4g01190v3 | AT1G54400, AT2G27140, AT5G17920, AT5G20970 |
| *30* | Zm00001eb034050 | SETIT_014289mg | - | AT4G27670 |
| *31* | Zm00001eb047490 | SETIT_037243mg, Si037243m.g | BRADI_3g27350v3 | AT4G21870, AT5G04890 |
| *32* | Zm00001eb052730 | SETIT_039412mg, Si039412m.g | BRADI_1g13261v3 | AT2G27140 |
| *33* | Zm00001eb124950 | Si002731m.g | BRADI_2g02410v3 | AT5G59720, AT1G53540 |
| *34* | Zm00001eb193940 | SETIT_018171mg, Si018171m.g | BRADI_3g58590v3 | AT5G51440 |
| *35* | Zm00001eb218280 | SETIT_039412mg, Si039412m.g | BRADI_1g13261v3 | AT2G27140 |
| *36* | Zm00001eb218310 | SETIT_038571mg, Si038571m.g | BRADI_1g13280v3 | AT1G76770, AT5G20970 |
| *37* | Zm00001eb222770 | SETIT_038857mg, Si038857m.g | BRADI_3g27360v3 | AT2G27140 |
| *38* | Zm00001eb222820 | SETIT_037243mg, Si037243m.g | BRADI_3g27350v3 | AT1G54400, AT2G27140, AT4G21870, AT5G20970, AT5G04890 |
| *39* | Zm00001eb235800 | SETIT_018295mg, Si018295m.g | BRADI_3g07421v3 | AT1G52560 |
| *40* | Zm00001eb297350 | Si022291m.g, Si022520m.g | BRADI_2g14460v3 | AT1G49970 |
| *41* | Zm00001eb322740 | SETIT_031104mg, Si031104m.g | BRADI_1g26190v3 | AT4G21870 |
| *42* | Zm00001eb372830 | SETIT_007151mg, Si007053m.g | - | AT4G25200, AT5G51440 |
| *43* | Zm00001eb050280 | SETIT_035859mg, Si035859m.g | BRADI_1g13640v3 | AT3G44110, AT5G22060 |
| *44* | Zm00001eb060050 | SETIT_035864mg, Si035864m.g | BRADI_1g06340v3 | AT5G22060, AT3G44110 |
| *45* | Zm00001eb091890 | SETIT_035864mg, Si035864m.g | BRADI_1g06340v3 | AT3G44110, AT5G22060 |
| *46* | Zm00001eb103670 | SETIT_035864mg, Si035864m.g | BRADI_1g06340v3 | AT5G22060, AT3G44110 |
| *47* | Zm00001eb107580 | SETIT_035864mg, Si035864m.g | BRADI_1g06340v3 | AT5G22060, AT3G44110 |
| *48* | Zm00001eb172900 | SETIT_013730mg, Si013730m.g | BRADI_4g41060v3 | AT2G22360, AT4G39960 |
| *49* | Zm00001eb192080 | SETIT_017013mg, Si017013m.g | BRADI_3g54550v3 | AT1G80030 |
| *50* | Zm00001eb213310 | SETIT_035864mg, Si035864m.g | BRADI_1g06340v3 | AT5G22060, AT3G44110 |
| *51* | Zm00001eb218560 | SETIT_035859mg, Si035859m.g | BRADI_1g13640v3 | AT3G44110, AT5G22060 |
| *52* | Zm00001eb249430 | SETIT_017322mg, Si017322m.g | BRADI_3g50610v3 | AT5G22060 |
| *53* | Zm00001eb258200 | SETIT_017013mg, Si017013m.g | BRADI_3g54550v3 | AT1G80030 |
| *54* | Zm00001eb269190 | SETIT_006434mg, Si006434m.g | BRADI_1g01507v3 | AT5G48030 |
| *55* | Zm00001eb294320 | SETIT_013730mg | BRADI_4g41060v3 | AT4G39960 |
| *56* | Zm00001eb379790 | SETIT_035864mg, Si035864m.g | BRADI_1g06340v3 | AT5G22060, AT3G44110 |
| *57* | Zm00001eb392350 | SETIT_013730mg | BRADI_4g41060v3 | AT2G22360, AT4G39960 |
| *58* | Zm00001eb404330 | SETIT_035864mg, Si035864m.g | BRADI_1g06340v3 | AT5G22060, AT3G44110 |
| *59* | Zm00001eb407790 | SETIT_010208mg, Si010208m.g | BRADI_4g41597v3 | AT5G48030 |
| *60* | Zm00001eb416450 | SETIT_013730mg, Si013730m.g | BRADI_4g41060v3 | AT2G22360, AT4G39960 |
| *61* | Zm00001eb429820 | SETIT_035864mg, Si035864m.g | BRADI_1g06340v3 | AT3G44110, AT5G22060 |
| *62* | Zm00001eb009060 | SETIT_035409mg, Si035409m.g | BRADI_1g69564v3 | AT3G17830 |
| *63* | Zm00001eb042680 | SETIT_015787mg, Si015787m.g | BRADI_3g35190v3 | AT5G25530 |
| *64* | Zm00001eb118880 | SETIT_002113mg, Si002113m.g | BRADI_2g08330v3 | AT2G20560, AT4G28480 |
| *65* | Zm00001eb208880 | SETIT_017724mg, Si017724m.g | BRADI_3g02750v3 | AT5G25530 |
| *66* | Zm00001eb240640 | SETIT_018622mg, Si018369m.g | BRADI_3g11390v3 | AT2G20560 |
| *67* | Zm00001eb282660 | SETIT_010075mg, Si010075m.g | BRADI_2g34950v3 | AT3G62600 |
| *68* | Zm00001eb288550 | SETIT_022594mg, Si022594m.g | BRADI_2g24680v3 | AT1G11040, AT1G44160 |
| *69* | Zm00001eb295770 | SETIT_022498mg, Si022498m.g | BRADI_2g16660v3 | AT2G20560 |
| *70* | Zm00001eb339480 | SETIT_002113mg, Si002113m.g | BRADI_2g08330v3 | AT4G28480, AT2G20560 |
| *71* | Zm00001eb340880 | SETIT_004748mg, Si004748m.g | - | AT3G62600 |
| *72* | Zm00001eb343630 | SETIT_022498mg, Si022498m.g | BRADI_2g16660v3 | AT2G20560 |
| *73* | Zm00001eb345630 | SETIT_010075mg, Si010075m.g | BRADI_2g34950v3 | AT3G62600 |
| *74* | Zm00001eb354940 | SETIT_022453mg, Si022453m.g | BRADI_2g38210v3 | AT3G08910 |
| *75* | Zm00001eb366410 | SETIT_001806mg, Si001806m.g | BRADI_2g56620v3 | AT1G11040, AT1G44160 |
| *76* | Zm00001eb415590 | SETIT_014023mg, Si014023m.g | BRADI_3g16270v3 | AT5G25530 |
| *77* | Zm00001eb035420 | SETIT_013893mg, Si013893m.g | BRADI_3g40307v3 | AT1G76700 |
| *78* | Zm00001eb243690 | SETIT_019379mg, Si019379m.g | BRADI_3g45780v3 | AT1G77020 |
| *79* | Zm00001eb294570 | SETIT_022590mg, Si022590m.g | BRADI_2g17974v3 | AT4G39150 |
| *80* | Zm00001eb317080 | SETIT_030100mg, Si030100m.g | BRADI_4g34030v3 | AT1G76700, AT1G21080 |
| *81* | Zm00001eb342590 | SETIT_022590mg, Si022590m.g | BRADI_2g17974v3 | AT4G39150 |
| *82* | Zm00001eb361030 | SETIT_000953mg, Si000953m.g | BRADI_2g47575v3 | AT4G39150, AT2G21510 |
| *83* | Zm00001eb409280 | Si021519m.g | BRADI_4g06790v3 | AT1G74250 |
| *84* | Zm00001eb409300 | Si021519m.g | BRADI_4g06790v3 | AT1G74250 |
| *85* | Zm00001eb409350 | Si021519m.g | BRADI_4g06790v3 | AT1G74250 |
| *86* | Zm00001eb409390 | Si021519m.g | BRADI_4g06790v3 | AT1G74250 |
| *87* | Zm00001eb137150 | SETIT_003003mg, Si003003m.g | BRADI_2g13810v3 | AT1G68370 |
| *88* | Zm00001eb146070 | Si001806m.g | BRADI_2g56620v3 | AT1G11040, AT1G44160 |
| *89* | Zm00001eb162560 | SETIT_001487mg, Si001487m.g | BRADI_2g41050v3 | AT5G05750, AT3G57340 |
| *90* | Zm00001eb205970 | SETIT_017983mg, Si017983m.g | BRADI_3g07070v3 | AT3G12170, AT5G06910 |
| *91* | Zm00001eb236340 | SETIT_017983mg, Si017983m.g | BRADI_3g07070v3 | AT3G12170, AT5G06910 |
| *92* | Zm00001eb254160 | SETIT_018322mg, Si018322m.g, Si019930m.g | BRADI_3g59670v3 | AT1G24120 |
| *93* | Zm00001eb279930 | SETIT_006439mg | BRADI_1g44760v3 | AT1G24120 |
| *94* | Zm00001eb342290 | SETIT_001487mg, Si001487m.g | BRADI_2g41050v3 | AT5G05750, AT3G57340 |
| *95* | Zm00001eb346620 | SETIT_024091mg, Si024091m.g | BRADI_2g27830v3 | AT3G57340, AT5G49060 |
| *96* | Zm00001eb010540 | SETIT_012802mg, Si012802m.g | BRADI_5g11240v3 | AT1G26230 |
| *97* | Zm00001eb046580 | SETIT_034887mg, Si034887m.g | BRADI_3g28070v3 | AT3G23990 |
| *98* | Zm00001eb065160 | SETIT_034840mg, Si034840m.g | BRADI_1g00730v3 | AT2G28000 |
| *99* | Zm00001eb209960 | SETIT_016700mg, Si016700m.g | BRADI_3g00480v3 | AT1G55490, AT3G13470, AT5G56500 |
| *100* | Zm00001eb222300 | SETIT_034887mg, Si034887m.g | BRADI_3g28070v3 | AT2G33210, AT3G23990 |
| *101* | Zm00001eb268910 | SETIT_006135mg, Si006135m.g | BRADI_1g50270v3 | AT3G13470, AT5G56500, AT1G55490 |
| *102* | Zm00001eb286730 | SETIT_021598mg, Si021598m.g | BRADI_5g02890v3 | AT2G28000 |
| *103* | Zm00001eb294310 | SETIT_021450mg, Si021450m.g | BRADI_2g18260v3 | AT3G13860 |
| *104* | Zm00001eb378930 | SETIT_006135mg, Si006135m.g | BRADI_1g50270v3 | AT3G13470, AT5G56500, AT1G55490 |
| *105* | Zm00001eb001240 | SETIT_035102mg, Si035102m.g | BRADI_3g11280v3 | AT3G18190 |
| *106* | Zm00001eb008400 | SETIT_006022mg, Si006022m.g | BRADI_1g39090v3, BRADI_2g26420v3 | AT5G26360 |
| *107* | Zm00001eb111720 | SETIT_021480mg, Si021480m.g | BRADI_3g04130v3 | AT3G02530, AT5G16070 |
| *108* | Zm00001eb114320 | SETIT_026187mg, Si026187m.g | BRADI_4g15450v3 | AT3G03960 |
| *109* | Zm00001eb118030 | SETIT_029447mg, Si029447m.g | BRADI_1g37790v3, BRADI_3g33860v3 | AT1G24510 |
| *110* | Zm00001eb127430 | - | BRADI_2g16500v3 | AT5G20890 |
| *111* | Zm00001eb189870 | SETIT_035102mg, Si035102m.g | BRADI_3g11280v3 | AT3G18190 |
| *112* | Zm00001eb192630 | SETIT_009761mg, Si006193m.g, Si009761m.g | BRADI_5g17750v3, BRADI_1g49760v3 | AT3G20050 |
| *113* | Zm00001eb200400 | SETIT_026187mg, Si026187m.g | BRADI_4g15450v3 | AT3G03960 |
| *114* | Zm00001eb204950 | SETIT_006022mg, Si006022m.g | BRADI_2g26420v3 | AT5G26360 |
| *115* | Zm00001eb207780 | SETIT_016800mg, Si016800m.g | BRADI_3g04430v3 | AT3G11830 |
| *116* | Zm00001eb257580 | SETIT_009761mg, Si006193m.g, Si009761m.g | BRADI_1g49760v3 | AT3G20050 |
| *117* | Zm00001eb298950 | SETIT_029447mg, Si029447m.g | BRADI_3g33860v3 | AT1G24510 |
| *118* | Zm00001eb333890 | Si001003m.g | BRADI_2g16500v3 | AT5G20890 |
| *119* | Zm00001eb017830 | SETIT_038207mg, Si038207m.g | - | AT1G23100 |
| *120* | Zm00001eb024030 | SETIT_037997mg, Si037997m.g | BRADI_3g33770v3 | AT2G44650, AT3G60210 |
| *121* | Zm00001eb071630 | SETIT_037997mg | BRADI_3g33770v3 | AT2G44650, AT3G60210 |
| *122* | Zm00001eb110270 | SETIT_031250mg, Si031250m.g | BRADI_1g19860v3 | AT1G23100, AT1G14980 |
| *123* | Zm00001eb290400 | SETIT_031250mg, Si013122m.g | BRADI_3G42020v3 | AT2G35110 |
| *124* | Zm00001eb329030 | SETIT_031250mg, Si031250m.g | BRADI_1g19860v3 | AT1G23100, AT1G14980 |
| *125* | Zm00001eb390720 | SETIT_037997mg, Si037997m.g | BRADI_3g33770v3 | AT2G44650, AT3G60210 |
| *126* | Zm00001eb100100 | SETIT_0309102mg, Si028830m.g | BRADI_4g31220v3 | AT5G20720 |
| *127* | Zm00001eb193040 | SETIT_018154mg, Si018154m.g | BRADI_3g60027v3 | AT5G20720 |
| *128* | Zm00001eb373960 | SETIT_009064mg, Si009064m.g | BRADI_1g46596v3 | AT5G20720 |
| *129* | Zm00001eb012470 | SETIT_034604mg, Si034604m.g | BRADI_1g66590v3 | AT3G12580 |
| *130* | Zm00001eb012510 | SETIT_008444mg, Si034600m.g | BRADI_1g66550v3 | AT1G16030 |
| *131* | Zm00001eb029200 | SETIT_021433mg, Si021433m.g | BRADI_4g04220v3 | AT3G12580 |
| *132* | Zm00001eb043470 | SETIT_034537mg | BRADI_3g57450v3 | AT4G37910 |
| *133* | Zm00001eb053750 | SETIT_021389mg | BRADI_2g30560v3 | AT5G49910 |
| *134* | Zm00001eb101990 | SETIT_029041mg, Si029041m.g | BRADI_4g33878v3 | AT5G09590 |
| *135* | Zm00001eb133480 | SETIT_021359mg, Si021359m.g | BRADI_4g39470v3 | AT4G24280, AT5G49910 |
| *136* | Zm00001eb136490 | SETIT_021433mg, Si021433m.g | BRADI_4g04220v3 | AT3G12580 |
| *137* | Zm00001eb148420 | SETIT_000619mg, Si000619m.g | BRADI_2g54570v3 | AT3G12580 |
| *138* | Zm00001eb165400 | SETIT_028356mg, Si028356m.g | - | - |
| *139* | Zm00001eb209550 | SETIT_016582mg, Si016582m.g | BRADI_3g01477v3 | AT5G42020, AT5G28540 |
| *140* | Zm00001eb214940 | SETIT_034533mg | - | AT1G09080, AT5G28540 |
| *141* | Zm00001eb217010 | SETIT_021389mg, | BRADI_2g30560v3 | AT5G49910 |
| *142* | Zm00001eb220140 | SETIT_034537mg, Si034537m.g | BRADI_3g57450v3, BRADI_1g77637v3 | AT5G09590 |
| *143* | Zm00001eb229930 | SETIT_016582mg, Si016582m.g | BRADI_3g01477v3 | AT5G42020, AT5G28540 |
| *144* | Zm00001eb351340 | SETIT_021434mg, Si021434m.g | BRADI_2g54570v3, BRADI_2g23250v3 | AT3G12580 |
| *145* | Zm00001eb368000 | SETIT_000619mg, Si000619m.g | BRADI_2g54570v3 | AT3G12580 |
| *146* | Zm00001eb397600 | SETIT_034600mg, Si034600m.g | BRADI_1g66470v3 | AT5G02500 |
| *147* | Zm00001eb400020 | SETIT_034355mg, Si034355m.g | BRADI_1g69700v3 | AT2G32120 |
| *148* | Zm00001eb409950 | SETIT_021359mg, Si021359m.g | BRADI_4g39470v3 | AT4G24280, AT5G49910 |
| *149* | Zm00001eb115870 | Si028356m.g | - | - |
| *150* | Zm00001eb188700 | SETIT_016270mg, Si016270m.g | BRADI_3g53100v3 | AT4G16660 |
| *151* | Zm00001eb252410 | SETIT_016270mg, Si016270m.g | BRADI_3g53100v3 | AT4G16660 |
| *152* | Zm00001eb224550 | SETIT_005920mg, Si005920m.g | BRADI_1g32770v3 | AT1G11660 |
| *153* | Zm00001eb273690 | SETIT_005920mg, Si005920m.g | BRADI_1g32770v3 | AT1G11660 |
| *154* | Zm00001eb283640 | Si021202m.g | BRADI_2g33682v3 | AT1G79920 |
| *155* | Zm00001eb283800 | SETIT_021202mg, Si021202m.g | BRADI_2g33682v3 | AT1G79920 |
| *156* | Zm00001eb346940 | - | - | AT1G79920 |
| *157* | Zm00001eb346960 | SETIT_021202mg | - | AT1G79920, AT1G79930 |
| *158* | Zm00001eb101750 | - | - | AT5G56000 |
| *159* | Zm00001eb137900 | SETIT_021237mg, Si021237m.g | BRADI_4g06370v3 | AT3G07770 |
| *160* | Zm00001eb199230 | SETIT_013283mg, Si013283m.g | BRADI_3g38897v3 | AT2G04030 |
| *161* | Zm00001eb199590 | SETIT_013347mg, Si013346m.g | BRADI_3g39630v3 | AT5G56000 |
| *162* | Zm00001eb228120 | SETIT_005877mg, Si005877m.g | BRADI_1g30130v3 | AT4G24190 |
| *163* | Zm00001eb264470 | SETIT_021237mg, Si021237m.g | BRADI_4g06370v3 | AT3G07770 |
| *164* | Zm00001eb272290 | SETIT_005877mg, Si005877m.g | BRADI_1g30130v3 | AT4G24190 |
| *165* | Zm00001eb315880 | SETIT_028990mg, Si028990m.g | BRADI_4g32941v3 | AT2G04030 |
| *166* | Zm00001eb316410 | - | - | AT5G56000, AT5G56030 |
| *167* | Zm00001eb418610 | SETIT_009497mg, Si009497m.g | BRADI_5g02037v3 | AT5G52640 |
| *168* | Zm00001eb034130 | Si013347m.g | BRADI_3g39590v3 | AT5G56000 |
| *169* | Zm00001eb020330 | SETIT_034086mg, Si034086m.g | BRADI_1g16190v3 | AT5G15450 |
| *170* | Zm00001eb084420 | SETIT_009283mg, Si009283m.g | BRADI_5g08920v3 | AT5G51070 |
| *171* | Zm00001eb084890 | SETIT_009304mg, Si009304m.g, Si009306m.g | BRADI_3g44335v3 | AT3G45450, AT5G50920 |
| *172* | Zm00001eb234160 | SETIT_016214mg, Si016214m.g | BRADI_3g06107v3 | AT2G25140 |
| *173* | Zm00001eb242420 | SETIT_016236mg, Si016236m.g | BRADI_3g44640v3 | AT5G51070 |
| *174* | Zm00001eb293780 | SETIT_021144mg, Si021144m.g | BRADI_2g19540v3 | AT1G74310 |
| *175* | Zm00001eb393250 | SETIT_034086mg, Si034086m.g | BRADI_1g16190v3 | AT5G15450 |
| *176* | Zm00001eb411490 | SETIT_021139mg, Si021139m.g | BRADI_4g39880v3 | AT5G50920 |
| *177* | Zm00001eb421360 | SETIT_009304mg, Si009304m.g | BRADI_3g44335v3 | AT5G50920 |
| *178* | Zm00001eb086360 | SETIT_012582mg, Si012582m.g | - | AT4G29920 |
| *179* | Zm00001eb178400 | SETIT_013176mg, Si013176m.g | BRADI_3g19077v3 | AT4G30350, AT5G57710 |
| *180* | Zm00001eb197530 | SETIT_025977mg, Si025977m.g | - | AT3G52490 |
| *181* | Zm00001eb412800 | SETIT_013176mg, Si013176m.g | BRADI_3g19077v3 | AT4G30350, AT5G57710 |
| *182* | Zm00001eb420410 | SETIT_012582mg, Si012582m.g | - | AT5G57130 |

**Table S8** List of motifs identified in each ZmHSP family. Colors are related to motifs illustrated in Figures S1 to S7:

| **Family** | **#** | **Motif amino acid sequence** |
| --- | --- | --- |
| **Zm**  **HSP100s**  **(CLPs)** | 1 | \|HKRVIGQDEAVKAISRAIRRSRVGLSDPNRPIASFLFMGPTGVGKTELAKALAAYYFGSEEAMIRJDMSEYMERHTVSKLIGSPPGYVGYEEGGQLTEAVRRRPYTVVLFDEIEKAHPDVFNILLQILEDGRLTDSQGRTVSFKNTLIIMTSNVGSSVI\| |
|  | 2 | \|MDAANLLKPALARGELQCIGATTLDEYRKHIEKDPALERRFQPVKVPEPSVEDTISILRGLRERYELHHKVRYTDEALVAAAQLSDRYISDRFLPDKAIDLIDEAGSRVRMEITSKP\| |
|  | 3 | \|GKLDPVIGRDDEIERVVQILSRRTKNNPVLIGEPGVGKTAIAEGLAQRIVNGDVPETLEGKRLISLDMGLLIAGAKYRGEFEERLKAVLKEVKES\| |
|  | 4 | \|YERIKSLVMEELKQYFRPEFLNRLDEMIVFRPLEKEZIKEIADJQLKEVQDRLKAKKINLZVTEKAKELIVSEGYDPSYGARPLRRAIQQLVEDELAEGILAGEFKEGDSILV\| |
|  | 5 | \|AVQZPFTERAAKVJVLAQEEARRLGHNQVTPEHLLLGLL\| |
| **Zm**  **HSP90s** | 1 | \|KEDYNKFWEAFGKNJKLGIIEDSQNRKRJAELLRFHSTKSGDELTSLDEYVTRMKEGQKDIYYITGESKKAVENSPFLEKLTKKGYEVLYMVDPIDEYAIQNLKEYEDKKFVDISKEGLKL\| |
|  | 2 | \|KEVLGDKVEKVVISBRLVDSPCVLVTGKYGWSANMERJMKAQTLGDSSMLAYMRSKKVMEINPEHPIIKELRKRAENDPEDKEVKDAVMLLFETALJTSGFSLDDPN\| |
|  | 3 | \|EKFEFQAEVNRLMDLIINSLYSNKEIFLRELISNASDALDKJRFLSLTDKSKLDAG\| |
|  | 4 | \|DVSMIGQFGVGFYSAYLVADRVVVTTKHNDDEQYVWESQAGGSFTVTRDTSGEQLGRGTKITLFLKDDQLEYLEERRLKDLVKKHSEFISYPISLWTEKTTEKEISDDEDE\| |
|  | 5 | \|DEKEEKEKKKKTIKEVSHEWELVNEQKPIWMRKPKEITKEEYAAFYKSLTNDWEDHLAV\| |
|  | 6 | \|NDEELIPRYLSFVKGIVDSNDLPLNVSREILQZNKILKIIRKRLVRKAFDMIFEIAE\| |
|  | 7 | \|IRIVPDKANNTJTIIDSGIGMTKSDLVBNLGTIARSGTKEF\| |
|  | 8 | \|HFTVEGZVEFKAVLFVPKRAPFDLFDTRK\| |
| **Zm**  **HSP70s** | 1 | \|DLGGGTFDVSVLTIEEGVFEVLATAGDTHLGGEDFDNRLVNHFVQEFKRKYKIDISKBPRALRRLRTACEKAKRTLSSTAZTTIEIESLYEGIDFYKTI\| |
|  | 2 | \|FSAEEISAMVLTKMKEIAEAYLGKTVKDAVITVPAYFNDSQRQATKDAGVIAGLNVLRIINEPTAAAJAYGLD\| |
|  | 3 | \|VMTVLIPRNTTIPTKKSQVFSTYADNQTGVLIQVYZGERERTKDNNLLGKFELSGIPPAPRGVPQIEVTFDIDANGILNVSA\| |
|  | 4 | \|GEGPAIGIDLGTTYSCVGVWQHDRVEIIANDQGNRTTPSYVAFTDSERLIGDAAKNQVAMNPTNTVFDAKRLIGRRFSDASVQSDMKLWPFKVI\| |
|  | 5 | \|FNGKEPCKTINPDEAVAYGAAVQAAILSG\| |
|  | 6 | \|TRARFEELNMDLFRKCMEPVEKCLRDAKL\| |
|  | 7 | \|VHEVVLVGGSTRIPKVQQLLQ\| |
| **Zm**  **CPN60s** | 1 | \|AISAGNEYEIGNMIAEAMEKVGREGVVTIEEGKSLENELEVVEGMQLDRGYISPYFVTBSEKMTVEYENCKILJVDKKITNARELIPVLEEAIKKRYPLLIIAEDVEGEALATLVINKLRGGLKVAAIKAPGFGERRKQYLDDIAILTGGEVIREELGL\| |
|  | 2 | \|ENPAAKLLVZLARAQBDEAGDGTTTVVVLAGELLKEAZKLIAAGIHPVSIIRGYEKA\| |
|  | 3 | \|ATIVLRGANDHVLDEAERSLHDALCVARNTLRBNRVVPGGGATEIELSKYLEZLARKLPGVEQYAIAAFAEALEAIPRTLAENAGLDAIDIITQLRAEHAK\| |
|  | 4 | \|SLEKADKEVLGTAKKVTVSKDSTTIVGDGSTKDEIEERVAQIKNEIEASESEYDKEKLNERIAKLSGGVAVIKVGAATETELKEKKLRVEDALNATKAAVEEGIVPGGGCALL\| |
|  | 5 | \|ADIVRTTLGPKGMBKMLESKYGGPKVTND\| |
|  | 6 | \|YGYNAATGEYEDLMAAGIIDPTKVVRCALZHAASVAKTFLTTEAI\| |
| **Zm**  **CPN10s** | 1 | \|DAKRLKPSNBRVLVEKLEAPEKTAGGILLPETAKQLEAAKV\| |
|  | 2 | \|RDLAGNLIPVEVEEGDTVLLSEYGGTEVKLA\| |
|  | 3 | \|DKEHLLFREDDILGV\| |
| **Zm**  **HSP40s**  **(DNAJs)** | 1 | \|VEKGMKKGQKITFPGEGBEAPBTVPGDJVFVJQZKPHPKFKRDGBDLFVTHTJTL\| |
|  | 2 | \|AGRDYYEVLGVSKDASQDEJKKAYRKLAL\| |
|  | 3 | \|DKNPDKPEAEEKFKEJSZAYEVLSDPEKRAIYD\| |
|  | 4 | \|HRSQIIFSDPASAGAKSASTVPDLFAFFSSSAFSGFSDTGRGFYKVYGDVFDRVFAQELAYARRMGVPEPAAPPVIGNLDSPYAQVTAFYSYWLGFGSGMDFGWAAAWDAARGESRRVRRLMEEDNKKAMRKARREYNDAVRGLAAFCKKRDKRVVDMVLKKKLEEEKRKAEEKERRKEEDKRKKERAMAYQEHEWARAE |
|  | 5 | \|IYQRPFMKGDLYIHFSVEFPKRLSSEQKK\| |
|  | 6 | \|GGGSSRGRRQRKGDDVEYPLKVSLEDLYNGTTKKIKJSRNV\| |
|  | 7 | \|QQMQHPCNECKGSGETISDKDRCPQCKGDKVVPEKKVLEVV\| |
|  | 8 | \|NEQEARAKLQEKIKEVQREREZKLAQSLKDRLQPYVDGRKDEFVSYASAEARRLSEAAFGEAMLHTIGYIYVRQAAKELGKSRIYLGVPFIAEWVRDKGHHIKSQVTAASGAISLIQLQE\| |
| **Zm**  **Small**  **HSPs** | 1 | \|RRFRLPENADVDGVRAALEBGVLTVTVPK\| |
|  | 2 | \|AHVFRVDVPGLRKEDVKVZVE\| |
|  | 3 | \|NVLVISGERRREEEEKGDKWH\| |
|  | 4 | \|TYASDAAAFAAARIDWKETPE\| |
|  | 5 | \|MSLIRRSNVFDPFSLDLW\| |
|  | 6 | \|MFGLETPLVAALHHLLDVPDGDAAAGGDK\| |
|  | 7 | \|KKPEVKEIKIS\| |
|  | 8 | \|VERSSGKF\| |

**Table S9** Codes of the protein families related to the conserved domains (CD) identified in each ZmHSP family, according to data from the PFAM and PROSITE platforms:

**ZmHSP100s/CLPs**: **ATPases AAA+** (PF00004, PF07724, PF07728, PF13173, PF13191 and PF13401); **AAA** (PF17871 and PF07724); **CLP-A/Bs chaperones** (PS00870); **CLP-B** (PF10431); **CLP-R** (PS51903); **CLP-N** (PF02861).

**ZmHSP90s**: **HSP90s** (PF00183 and PS00298); **PGbinding-1** (PF15871); **ATPases HSP90s-like** (PF02518 and PF13589); **C-domain in ABC-3C systems** (PF20283).

**ZmHSP70s**: **HSP70s** (PF00012, PS00297 and PS01036); **MreB/Mbl** (PF06723); **GLE1** (PF07817).

**ZmCPN60s**: **TCP-1/CPN60** (PF00118, PS00296 and PS00750); **GroEL/TCP-**1 (IPR002423); **TOPRIM** (PF01751); **RFC** (PF08542); **AbiEi** (PF13338); **FLGM** (PF04316).

**ZmCPN10s**: **10 kDa chaperonin subunits** (PF00166).

**ZmHSP40s/DNAJs**: **J-domain N-terminal** (PF00226 and PS50076); **J-domain C-terminal** (PF01556); **J-domain X-region** (PF14308); **J-domain central-region** (PF00684); **Anti-TRAP** (PF15777); **HypA** (PF01155); **IDO** (PF01231).

**ZmSmallHSPs**: **HSP20** (PF00011, PS01031 and PF17886); **CS** (PF04969); **GRAS** (PF03514); **L6** (PF00347); **BOM** (PF04972); **FKBP26** (PF18046); **GvpH** (PF05455).

**Table S10** List of ZmHSPs that had their expression profiles significantly altered after exposure to certain types of abiotic stresses:

ZmHSPs that had their expression levels multiplied hundreds or thousands of times after exposure to **excessive heat on maize seedlings**: **sHSPs** (Zm00001eb011930, Zm00001eb124940, Zm00001eb124960, Zm00001eb335430, Zm00001eb397940, Zm00001eb121900, Zm00001eb257130, Zm00001eb337690, Zm00001eb395330, Zm00001eb395360, Zm00001eb395370, Zm00001eb081770, Zm00001eb112330, Zm00001eb125030, Zm00001eb252460, Zm00001eb371530, Zm00001eb423300, Zm00001eb010530, Zm00001eb034050, Zm00001eb124950, Zm00001eb193940 and Zm00001eb235800); **HSP70s** (Zm00001eb148420, Zm00001eb217010, Zm00001eb351340, Zm00001eb368000, Zm00001eb397600 and Zm00001eb400020); **HSP90s** (Zm00001eb418610); **HSP100s** (Zm00001eb293780).

ZmHSPs that had their expression levels multiplied dozens of times after exposure to **cold or salinity on maize seedlings**: **sHSPs** (Zm00001eb011930, Zm00001eb124940, Zm00001eb124960, Zm00001eb335430, Zm00001eb397940, Zm00001eb121900, Zm00001eb257130, Zm00001eb337690, Zm00001eb395330, Zm00001eb395360, Zm00001eb395370, Zm00001eb081770, Zm00001eb112330, Zm00001eb125030, Zm00001eb252460, Zm00001eb371530, Zm00001eb423300, Zm00001eb010530, Zm00001eb034050, Zm00001eb124950, Zm00001eb193940 and Zm00001eb235800); **HSP70s** (Zm00001eb148420, Zm00001eb217010, Zm00001eb351340, Zm00001eb368000, Zm00001eb397600 and Zm00001eb400020); **HSP90s** (Zm00001eb418610); **HSP100s** (Zm00001eb293780).

ZmHSPs that had their expression levels increased between 5 to 10 times on **leaves and/or leaf meristems after exposure to drought**: **sHSPs** (Zm00001eb011930, Zm00001eb124940, Zm00001eb124960, Zm00001eb335430, Zm00001eb337690, Zm00001eb112330, Zm00001eb010530, Zm00001eb026690, Zm00001eb124950 and Zm00001eb235800); **HSP70s** (Zm00001eb397600 and Zm00001eb400020).

ZmHSPs that had their expression levels increased between 18 to 50 times on **fertilized ovaries after exposure to drought**: **sHSPs** (Zm00001eb335430 and Zm00001eb352720); **HSP70** (Zm00001eb397600); **HSP90** (Zm00001eb418610).

ZmHSPs that were significantly upregulated in tolerant inbred lines of tropical maize, after their **seeds had been dried at high** temperatures: **sHSPs** (Zm00001eb124940); **HSP90s** (Zm00001eb418610); **HSP100s** (Zm00001eb293780).

ZmHSPs without any kind of information, on RNA-Seq libraries and such, for variations in expression levels after exposure to abiotic stresses: (Zm00001eb043470, Zm00001eb214940 and Zm00001eb294320).

**Table S11** Percent identity matrix of three ZmHSPs groups, where the occurrence of gene duplication was conjectured:

| *on chromosome 01* | | | | *on chromosome 09* | | | | *on chromosome 10* | | | | |
| --- | --- | --- | --- | --- | --- | --- | --- | --- | --- | --- | --- | --- |
|  | **sHSP-A01** | **sHSP-A02** | **sHSP-A03** |  | **sHSP-B05** | **sHSP-B06** | **sHSP-B07** |  | **HSP40-D01** | **HSP40-D02** | **HSP40-D03** | **HSP40-D04** |
| **sHSP-A01** |  | 99,0 | 66,5 | **sHSP-B05** | #### | 98,2 | 98,3 | **HSP40-D01** | #### | 59,2 | 79,3 | 78,8 |
| **sHSP-A02** | 99,0 |  | 66,2 | **sHSP-B06** | 98,2 | #### | 99,7 | **HSP40-D02** | 59,2 | #### | 99,2 | 99,0 |
| **sHSP-A03** | 66,5 | 66,2 |  | **sHSP-B07** | 98,3 | 99,7 | #### | **HSP40-D03** | 79,3 | 99,2 | #### | 96,4 |
|  |  |  |  |  |  |  |  | **HSP40-D04** | 78,8 | 99,0 | 96,4 | #### |

**Table S12** List of target and normalizers genes, as well as their respective primers, used for the expression analyses on seeds from four tropical maize inbred lines, previously submitted to two drying temperatures (35 and 50°C):

| **Gene Name** | **Zm-B73-REF. NAM-5.0** | **Primer Forward (5'→3')** | **Primer Reverse (5'→3')** |
| --- | --- | --- | --- |
| sHSP-A04(Chr3-) | Zm00001eb124940 | ACTTCGCAGTTGGCAGACTC | GAAGAGGTCCACGGAGAATG |
| sHSP-D01(Chr1+) | Zm00001eb010530 | GTCTCCAACCAGGAAAGCAA | CTGGGGCTATGTGAATCTGG |
| sHSP-D10(Chr4-) | Zm00001eb193940 | CGCTTCAGTTTTCGTGTCAA | GGAAACCAAACACGGCATAG |
| HSP70-A8(Chr3- | Zm00001eb136490 | ACCACTTCGTCCAGGAGTTCAA | GCCCTCATACAGCGAGTCAATCT |
| HSP70-A18(Chr9+) | Zm00001eb397600 | GCAGGGTGTTCTCTGTTTCC | CGTGCATAGCGAGAAAAACA |
| HSP90-A10(Chr10+) | Zm00001eb418610 | TGTCGTGGAATCCTGTGAAA | TTTTCTCCCACCTTTTGTGG |
| HSP100-A06(Chr6+) | Zm00001eb293780 | TGAGGATCATGGAGGAGGAC | CCACATTCACGGGCTTATCT |
| Ubiquitin-60S Ribosomal | Zm00001eb009900 | AAGGCCAAGATCCAGGACAA | TTGCTTTCCAGCGAAGATGA |
| ADH 1 | Zm00001eb056510 | AGGACGCTGAGTTAAGACC | CACATTTGGCAGATCAGTGC |
| β-Actin 7 | Zm00001eb222460 | TGTCCATCACTTGTGAAGCCTCCT | ACGACCTTAGCCAATATCGCACCA |


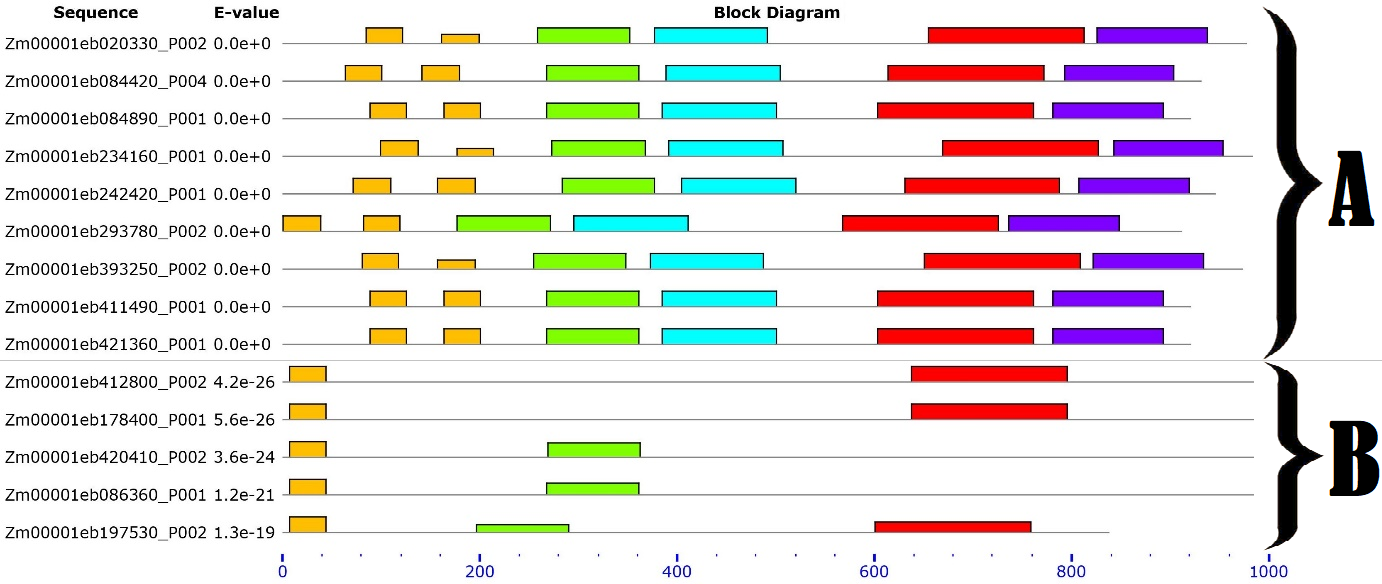


**Figure S1** Alignment of the fourteen validated ZmHSP100s/CLPs. ***Annotations:*** five motifs were identified during the search for possible conserved domains. Details about the amino acid sequence of these motifs are provided at the Table S8 (also placed in this Additional File 01). Based on these conserved regions, two ZmHSP100 classes/subfamilies were defined (A and B), among which we judge to have significant structural differences.


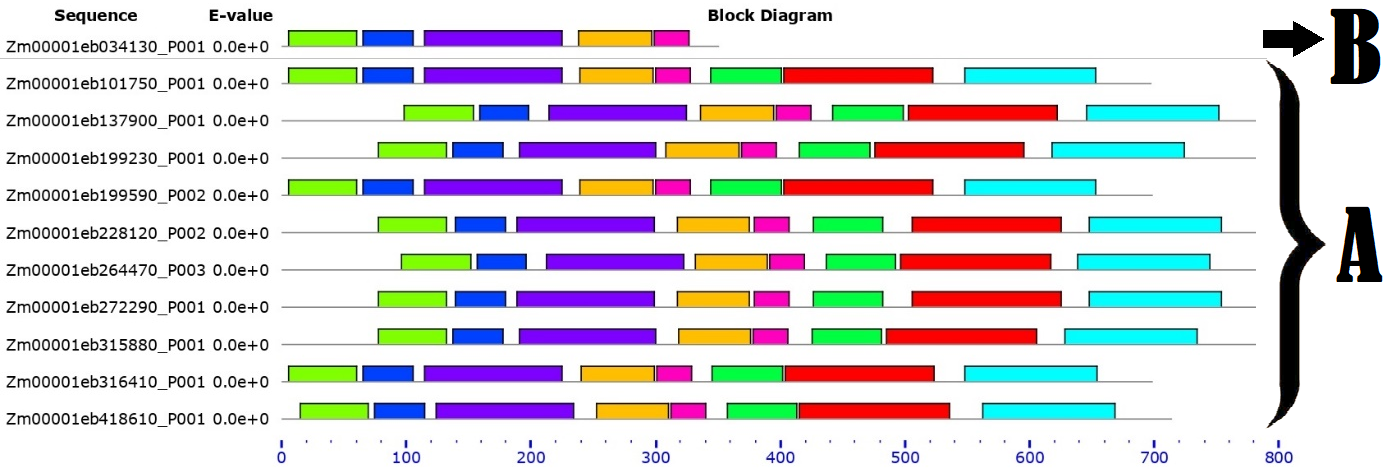


**Figure S2** Alignment of the eleven validated ZmHSP90s. ***Annotations:*** eight motifs were identified during the search for possible conserved domains. Details about the amino acid sequence of these motifs are provided at the Table S8 (also placed in this Additional File 01). Based on these conserved regions, two ZmHSP90 classes/subfamilies were defined (A and B), among which we judge to have significant structural differences.


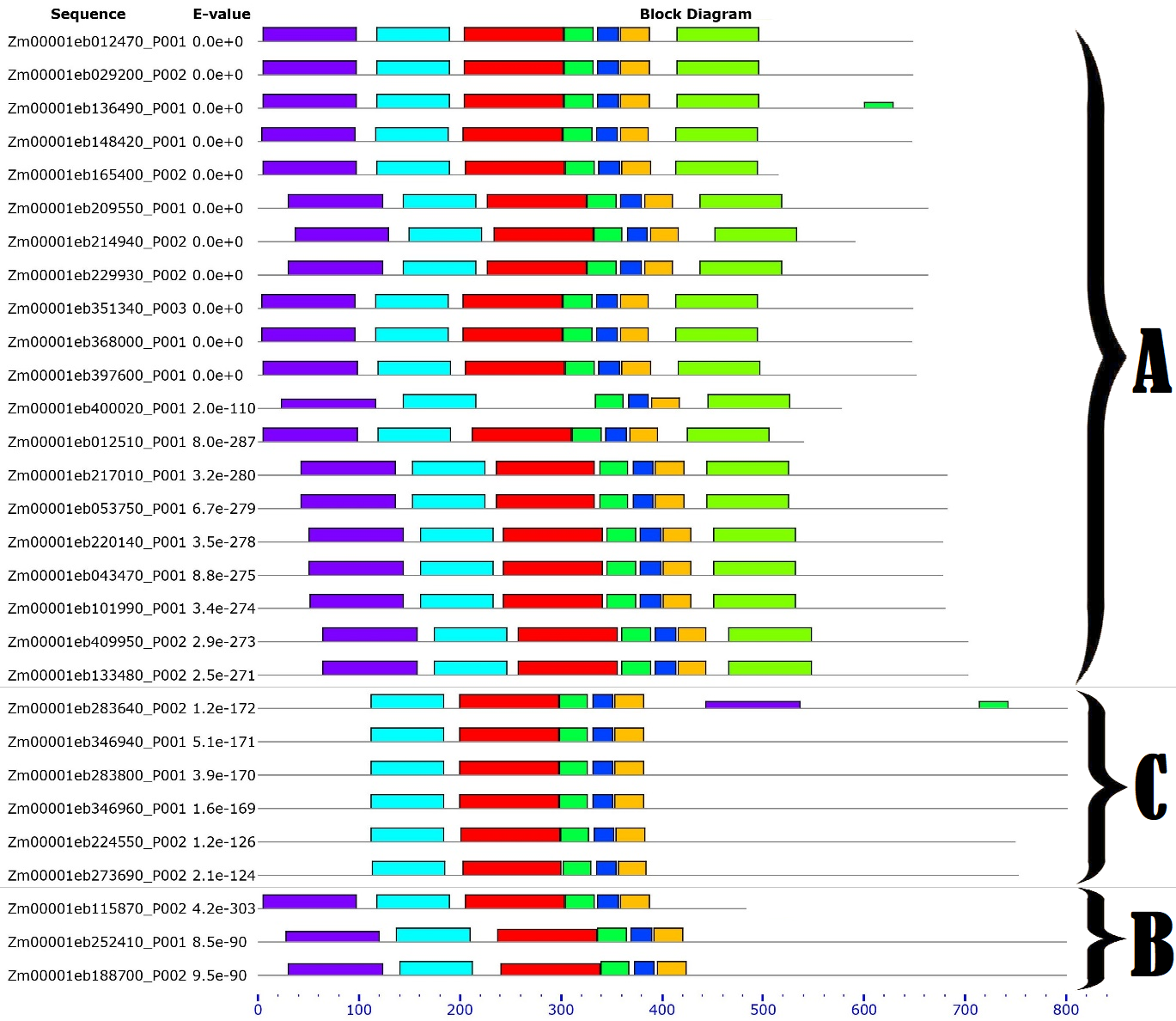


**Figure S3** Alignment of the twenty-nine validated ZmHSP70s. ***Annotations:*** seven motifs were identified during the search for possible conserved domains. Details about the amino acid sequence of these motifs are provided at the Table S8 (also placed in this Additional File 01). Based on these conserved regions, three ZmHSP70 classes/subfamilies were defined (A, B and C), among which we judge to have significant structural differences.


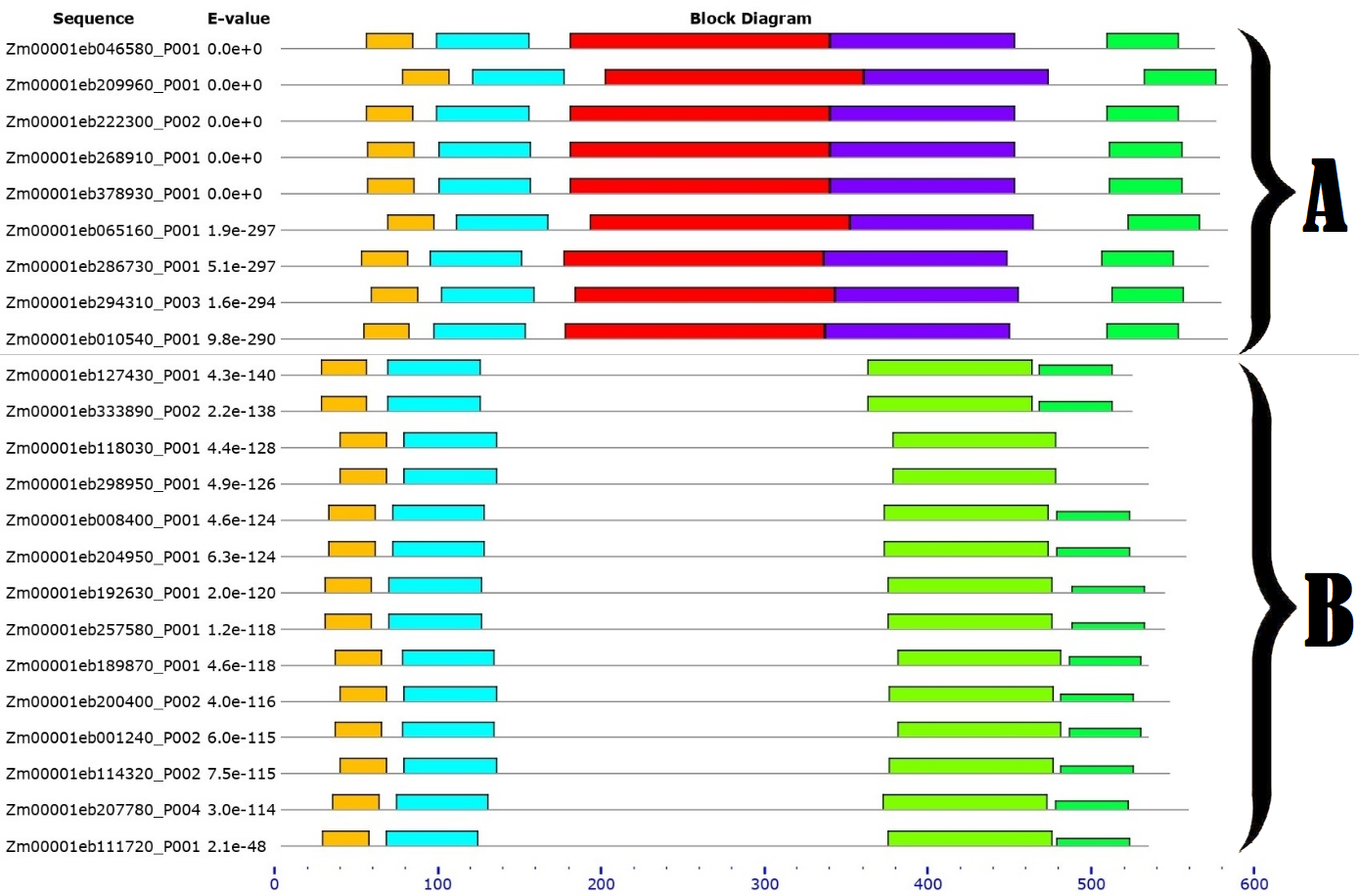


**Figure S4** Alignment of the twenty-three validated ZmCPN60s. ***Annotations:*** six motifs were identified during the search for possible conserved domains. Details about the amino acid sequence of these motifs are provided at the Table S8 (also placed in this Additional File 01). Based on these conserved regions, two ZmCPN60 classes/subfamilies were defined (A and B), among which we judge to have significant structural differences.


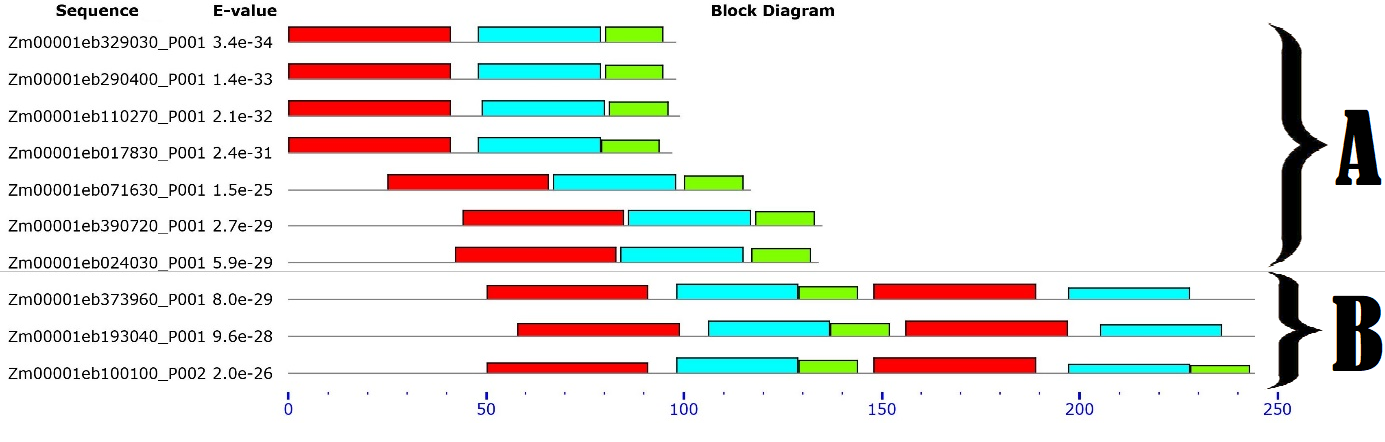


**Figure S5** Alignment of the ten validated ZmCPN10s. ***Annotations:*** three motifs were identified during the search for possible conserved domains. Details about the amino acid sequence of these motifs are provided at the Table S8 (also placed in this Additional File 01). Based on these conserved regions, two ZmCPN10 classes/subfamilies were defined (A and B), among which we judge to have significant structural differences.


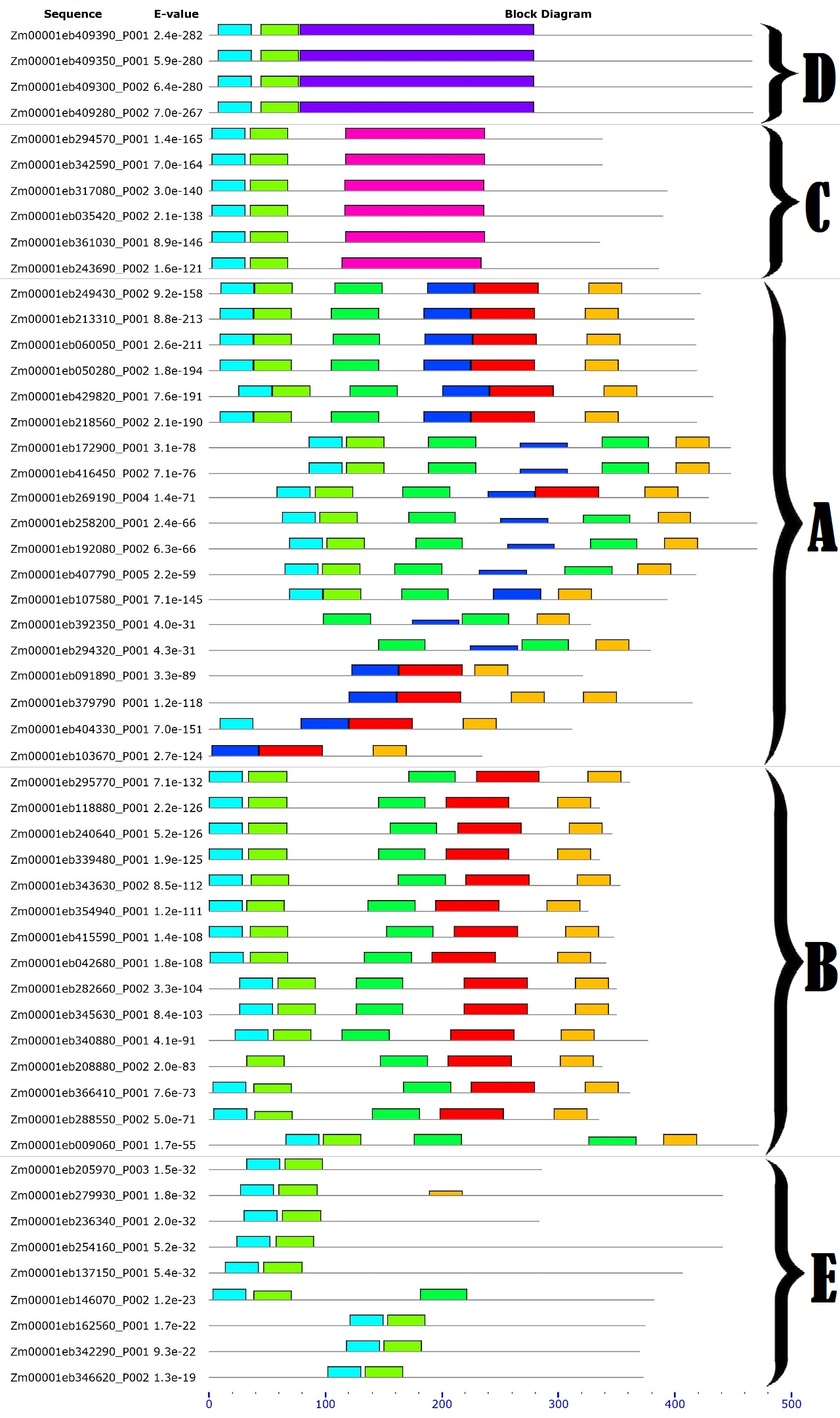


**Figure S6** Alignment of the fifty-three validated ZmHSP40s/DNAJs. ***Annotations:*** eight motifs were identified during the search for possible conserved domains. Details about the amino acid sequence of these motifs are provided at the Table S8 (also placed in this Additional File 01). Based on these conserved regions, five ZmHSP40 classes/subfamilies were defined (A, B, C, D and E), among which we judge to have significant structural differences.


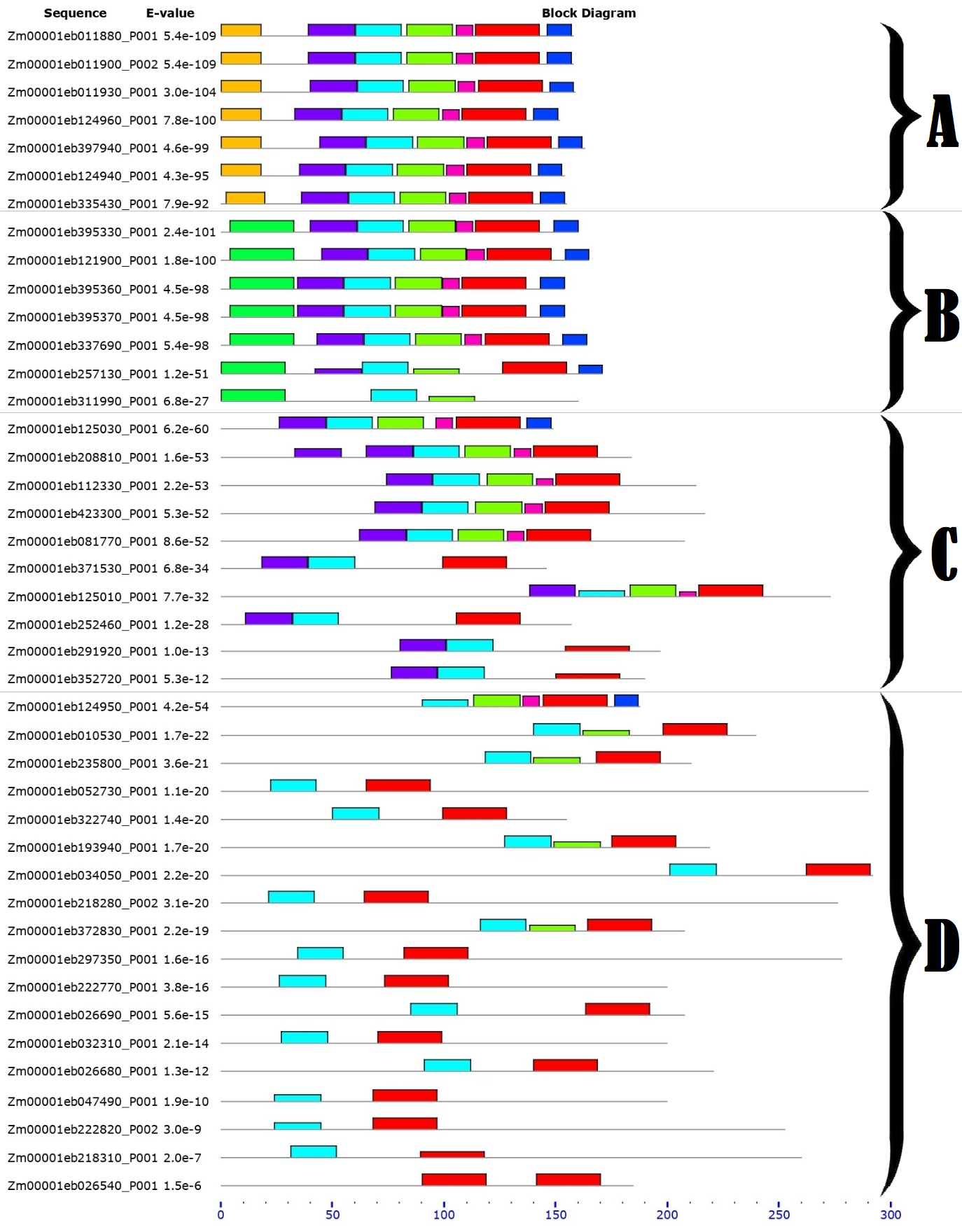


**Figure S7** Alignment of the forty-two validated ZmSmallHSPs. ***Annotations:*** eight motifs were identified during the search for possible conserved domains. Details about the amino acid sequence of these motifs are provided at the Table S8 (also placed in this Additional File 01). Based on these conserved regions, four ZmSmallHSP classes/subfamilies were defined (A, B, C and D), among which we judge to have significant structural differences.


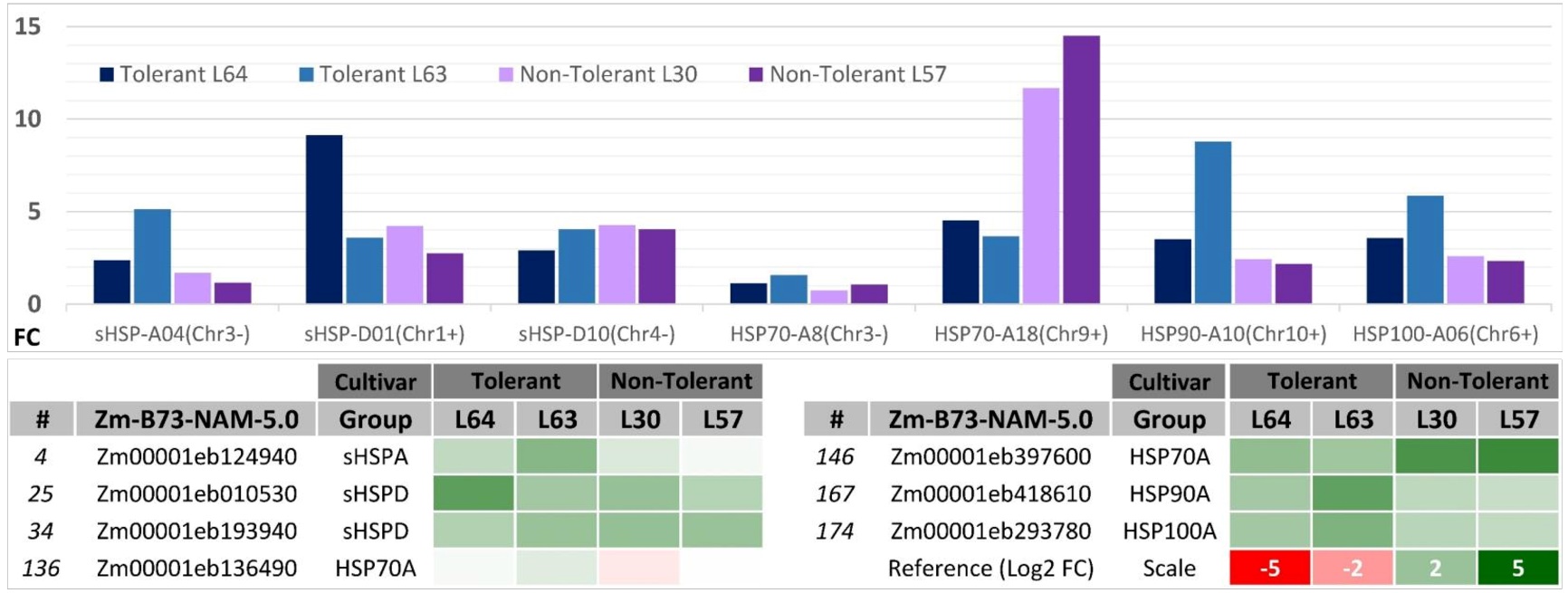


**Figure S8** Changes in the expression levels of seven ZmHSPs, measured after the seeds of four tropical maize inbred lines had been subjected to two different drying temperatures. ***Annotations:*** the seed drying temperatures used were: 35°C (considered the normal one) or 50°C (a stressful temperature). The maize inbred lines were previously classified according to their degree of tolerance to high drying temperatures: in two of them (L64 and L63), the results of the germination and seed vigor tests remained satisfactory even after the stress application (so, they were considered tolerant); in the other two inbred lines (L30 and L57), the seeds lost vigor significantly, in addition to have showed low germination rates after drying at high temperatures (therefore, they were considered non-tolerant). At the figure top (bar graph), the changes in the expression profiles are illustrated on a fold change scale: to obtain such values, we have just divided the relative expression obtained from seeds subjected to stress (seed drying temperature equal to 50°C) by the relative expression under normal conditions (seeds dried at 35°C). At the figure bottom, we see the heat map: these values, in turn, were plotted on a Log2 FC ratio, i.e. a binary logarithm scale to those fold change values; the heatmap caption (for a red-to-green scale) is located at the bottom right. The data that gave rise to both (bar graph and heatmap) are detailed in the Additional File 03.


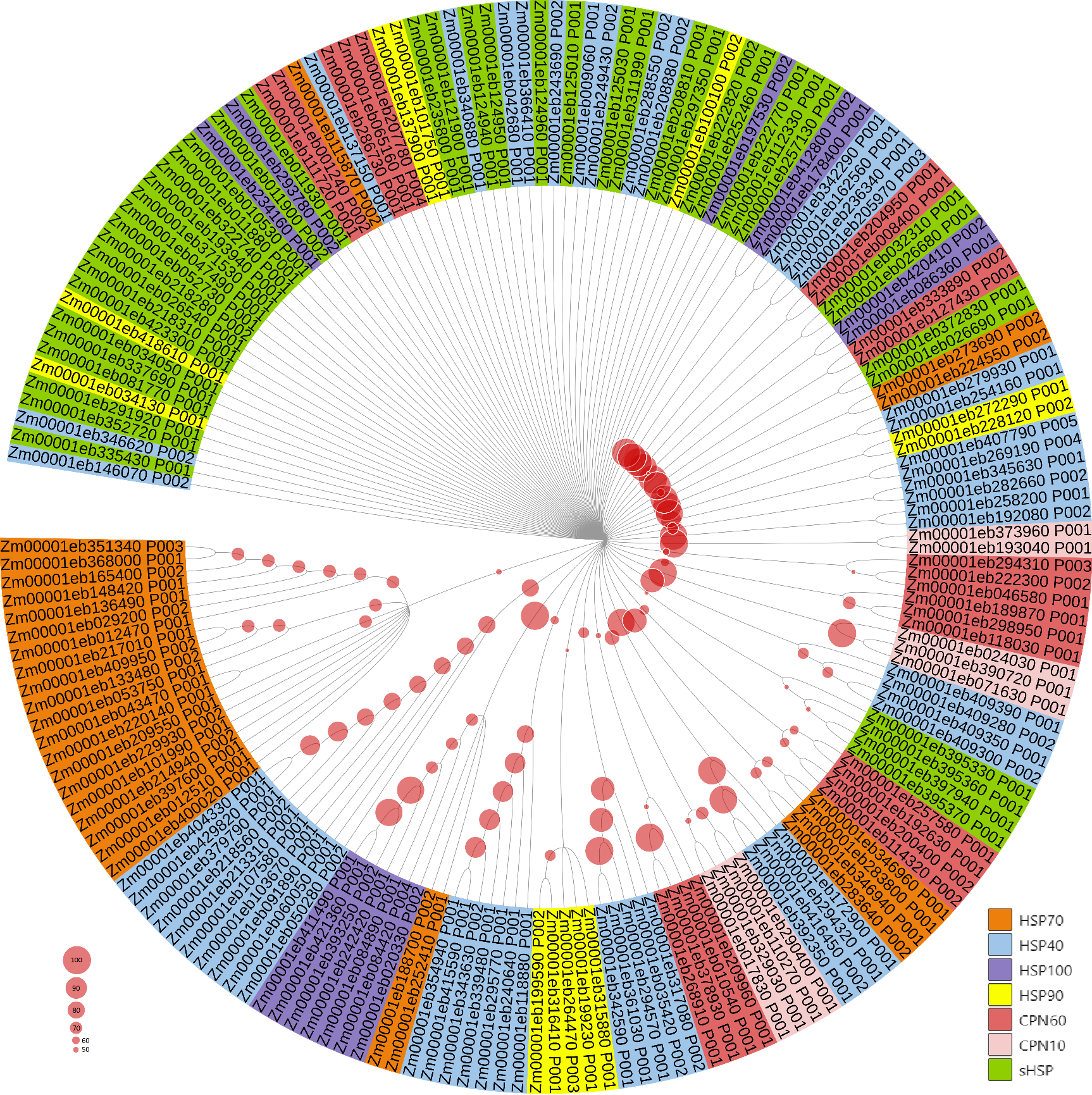


**Figure S9** Phylogenetic tree with the 182 validated ZmHSPs. ***Annotations:*** the inferred evolutionary relationships were based on their amino acid sequences. We have used the neighbor join method, with 1000 bootstrap replicas. The red spheres, with different sizes (50 to 100, caption at bottom left), represent the bootstrap values, indicating the percentage of times that the same branch is observed when the tree generation is repeated in a resampled data set. The colors that highlight the name of each ZmHSP refer to their respective families (caption at bottom right). The nomenclature is according to the most recent assembly version of the B73 maize genome (Zm-B73-REF.-NAM-5.0).
